# Single dose primaquine to reduce gametocyte carriage and *Plasmodium falciparum* transmission in Cambodia: An open-label randomized trial

**DOI:** 10.1371/journal.pone.0168702

**Published:** 2017-06-07

**Authors:** Jessica T. Lin, Chanthap Lon, Michele D. Spring, Somethy Sok, Soklyda Chann, Mali Ittiverakul, Worachet Kuntawunginn, Mok My, Kheangheng Thay, Rifat Rahman, Sujata Balasubramanian, Mengchuor Char, Charlotte A. Lanteri, Panita Gosi, Ratawan Ubalee, Steven R. Meshnick, David L. Saunders

**Affiliations:** 1 Division of Infectious Diseases, University of North Carolina School of Medicine, Chapel Hill, North Carolina, United States of America; 2 Armed Forces Research Institute of Medical Sciences, Phnom Penh, Cambodia; 3 Department of Immunology and Medicine, Armed Forces Research Institute of Medical Sciences, Bangkok, Thailand; 4 Royal Cambodian Armed Forces, Phnom Penh, Cambodia; 5 National Center for Parasitology, Entomology and Malaria Control, Phnom Penh, Cambodia; 6 Department of Entomology, Armed Forces Research Institute of Medical Sciences, Bangkok, Thailand; 7 Department of Epidemiology, Gillings School of Global Public Health, University of North Carolina, Chapel Hill, North Carolina, United States of America; University of California Los Angeles, UNITED STATES

## Abstract

**Background:**

Single low dose primaquine (SLD PQ, 0.25mg/kg) is recommended in combination with artemisinin-based combination therapy (ACT) as a gametocytocide to prevent *Plasmodium falciparum* transmission in areas threatened by artemisinin resistance. To date, no randomized controlled trials have measured primaquine’s effect on infectiousness to Anopheline mosquitoes in Southeast Asia.

**Methods:**

Cambodian adults with uncomplicated falciparum malaria were randomized to receive a single 45mg dose of primaquine (equivalent to three SLD PQ) or no primaquine after the third dose of dihydroartemisin-piperaquine (DHP) therapy. A membrane-feeding assay measured infectiousness to *Anopheles dirus* on days 0, 3, 7, and 14 of blood-stage therapy. Gametocytemia was evaluated by microscopy and reverse-transcriptase PCR.

**Results:**

Prior to trial halt for poor DHP treatment efficacy, 101 participants were randomized and 50 received primaquine. Overall microscopic gametocyte prevalence was low (9%), but gametocytemic subjects given primaquine were gametocyte-free by day 14, and significantly less likely to harbor gametocytes by day 7 compared to those treated with DHP-alone, who remained gametocytemic for a median of two weeks. Only one infectious subject was randomized to the primaquine group, precluding assessment of transmission-blocking efficacy. However, he showed a two-fold reduction in oocyst density of infected mosquitoes less than 24 hours after primaquine dosing. In the DHP-alone group, four subjects remained infectious through day 14, infecting roughly the same number of mosquitoes pre and post-treatment. Overall, microscopic gametocytemia was an excellent predictor of infectiousness, and performed better than submicroscopic gametocytemia post-treatment, with none of 474 mosquitoes infected post-treatment arising from submicroscopic gametocytes.

**Conclusions:**

In a setting of established ACT resistance, a single dose of 45mg primaquine added to DHP rapidly and significantly reduced gametocytemia, while DHP-alone failed to reduce gametocytemia and prevent malaria transmission to mosquitoes. Continued efforts to make single dose primaquine widely available are needed to help achieve malaria elimination.

## Introduction

Malaria containment efforts launched in western Cambodia over the last 7–8 years to curb artemisinin-resistant malaria have contributed to a marked decline in *Plasmodium falciparum* cases in the region, but have failed to prevent the emergence of slow-clearing parasites throughout Southeast Asia [1–3]. Cambodia adopted dihydroartemisinin-piperaquine (DHP) nationally as its first-line ACT in 2012, though significant clinical failures were reported the following year [4–6]. As few therapeutic options remain in the Mekong Subregion, it is even more important to pursue transmission-blocking interventions to help achieve regional malaria elimination.

In 2010, the WHO recommended that single dose primaquine (45mg or 0.75 mg/kg, later revised to 0.25mg/kg in 2012) be used as a gametocytocide in combination with ACT to prevent transmission of *P*. *falciparum* to mosquitoes in areas threatened by artemisinin resistance [7]. At the time, there was good clinical evidence that primaquine added to ACTs substantially reduced gametocytes, the parasite stages responsible for transmission to mosquitoes [8–11]. However, actual entomological evidence of transmission-blocking to mosquitoes was limited to small non-randomized case series [12,13]. Earlier this year, a randomized controlled trial in Mali was the first to show that single low dose primaquine, given alongside DHP, was indeed efficacious for preventing malaria transmission to *Anopheles gambiae* mosquitoes in West Africa [14].

In 2012–2014, we similarly undertook an open label randomized trial to determine the transmission-blocking efficacy of primaquine added to DHP in western Cambodia. We used membrane feeding assays to measure infectiousness to *Anopheles dirus* mosquitoes, the major malaria vector in the region. Unfortunately, DHP failure in the cohort was unexpectedly high indicating rapid progression of clinical resistance, and the trial had to be halted early, as previously reported [5]. Here we report on the mosquito infectivity and gametocyte outcomes of the 101 participants randomized before the trial was halted. The overall rate of infectiousness in the cohort was low and precluded an ability to establish definitive transmission-blocking efficacy, but we saw a significant reduction in gametocytes post-treatment in those given the 45mg dose of primaquine. In contrast, subjects treated with DHP-alone without primaquine were slow to clear gametocytes, and individuals continued to infect mosquitoes two weeks post-treatment.

## Methods

### Study design and participants

This study was carried out between 10 December 2012–24 February 2014 in Oddar Meanchey Province, northwestern Cambodia. Malaria transmission is low, heterogeneous, and seasonal with entomological inoculation rates generally below one/person/year. The majority of clinical cases occur during the rainy season between May and December [2]. This was an open-label randomized clinical trial that enrolled adults aged 18–65 years presenting or referred to Anlong Veng District Hospital with uncomplicated *P*. *falciparum* or mixed P. *falciparum/P*. *vivax* (Pf/Pv) infection diagnosed with microscopy and confirmed with real-time polymerase chain reaction (PCR). Testing for glucose-6-phosphate dehydrogenase (G6PD) deficiency was carried out using both the fluorescent spot test (R&D Diagnostics Ltd, Greece) and quantitative testing of enzyme activity (Trinity Biotech, Ireland). Participants with severe deficiency (WHO Class I or II) defined as 10% or less of the lower limit of normal activity were excluded, while those with mild or moderate G6PD deficiency were included in the trial. More details of the study design have been previously presented [5]. Participants provided written informed consent after referral from various medical facilities in Oddar Meanchey Province. Ethical approval for the study was obtained from the Walter Reed Army Institute of Research (14 September 2012), National Ethics Committee for Health Research in Cambodia (21 October 2011), and University of North Carolina (25 September 2012). The trial was registered with ClinicalTrials.gov, number NCT01849640.

### Procedures

All participants received three doses of dihydroartemisinin-piperaquine (40mg and 320mg per tablet) as directly observed therapy over 3 days (0, 24, and 48h from baseline). After the third dose, subjects were randomly allocated 1:1 to receive 45mg of primaquine or no primaquine. This dose typically occurred 52 hours from baseline (on day 2). Participants were released to outpatient follow-up on day 3 (72 hours) or once afebrile with two consecutive negative smears, returning for weekly follow-up visits until day 42.

Giemsa-stained thick and thin blood smears collected at baseline, every 8 hours during treatment, and at weekly follow-up visits were examined by two microscopists blinded to each other’s results, with gametocytes counted per 2000 white blood cells [15]. Molecular detection of gametocytes at baseline, day 7, and day 14 was carried out using reverse-transcriptase PCR (RT-PCR) of Pfs25 [16–18]. This assay has a reported detection limit of 1–2 gametocytes/μL when applied to blood spots [16]. Hemoglobin was measured at baseline and daily until discharge on day 3. It was also measured in G6PD-deficient subjects at their follow-up visit on days 7 and 14, but not routinely checked for G6PD-normal subjects after day 3.

Mosquito infectivity was measured in all study participants, regardless of gametocyte status, via membrane feeding experiments at baseline (day 0); and days 3 (72 hours from baseline), 7, and 14 post-treatment. For each experiment, 2mL of fresh venous blood was fed to ~300 colony-reared female *Anopheles dirus* mosquitoes, with the goal of yielding approximately 200 engorged (fed) mosquitoes. Further details on the membrane feeding assay have been previously published [18]. Nine days after feeding, mosquito midguts from 50 mosquitoes were examined for the presence of oocysts following dissection in 2% mercurochrome, with oocyst counts confirmed by two independent readers. Another 50 mosquitoes at day 9 were saved for molecular detection to confirm the presence of oocysts and perform speciation of parasites. At day 16 post-feeding, the remaining mosquitoes were collected for molecular detection of sporozoites [18]. In particular, 18s rRNA PCR performed on DNA extracted from individual mosquitoes was used to help confirm the presence of *P*. *falciparum* infection in mosquitoes fed on subjects with mixed Pf/Pv infection.

### Outcomes

We assessed the transmission-blocking efficacy of a 3-day dose of DHP with or without a single oral 45 mg dose of primaquine by comparing the proportion of individuals infecting at least one mosquito out of fifty, at one and two weeks post-treatment in the two arms. We also assessed the effect of the two treatment regimens on the risk of gametocyte carriage as measured by microscopy and RT-PCR. This was done by comparing gametocyte prevalence at weekly timepoints post-treatment and the time to gametocyte clearance in the two arms. We also analyzed the number of infected mosquitos per treatment arm, the relationship of gametocytemia to mosquito infectivity, and within-person changes in hemoglobin four days post-primaquine treatment among volunteers with G6PD-deficiency.

### Statistical analysis

The study was not powered for transmission-blocking efficacy, but for the main primary objective of measuring the therapeutic efficacy of DHP with and without primaquine [5]. A sample size of 150 evaluable subjects was chosen to yield a 95% CI of 89–97% around a true efficacy estimate of 94%. We entered data into a Microsoft Access 2007 database, with 100% clinical source data verification by the study monitor. Statistical analyses were performed using Stata version 12.1. In this analysis, we included all subjects that were randomized to primaquine on day 2. Differences in the proportion of infectious individuals and gametocyte prevalence were compared using χ2 test or Fisher’s exact test as appropriate. Unadjusted Kaplan-Meier survival analysis was used to measure the risk of persistence of gametocytes in those gametocytemic on day 2, with log-rank testing used for comparison and hazard ratio generated using a Cox proportional hazards model. Other comparisons used Fisher’s exact test for categorical variables and Student’s t test or Wilcoxon rank-sum test (for non-normally distributed data) for continuous variables. We regarded a one-sided p value of less than 0.05 significant when evaluating the effect of primaquine on infectiousness and gametocyte carriage; otherwise a two-sided p-value was used.

## Results

### Study population

Out of 107 subjects who were enrolled and given dihydroartemisinin-piperaquine (DHP) therapy, six withdrew from the study before being randomized, leaving 101 who were randomized to primaquine (PQ) or no primaquine on Day 2; 50 received DHP+PQ and 51 DHP-only [5]. There were no differences in baseline demographics between primaquine and non-primaquine groups (Table 1). Most participants were male (98/101) and either farmers (83/101) or military personnel (11/101). Roughly sixty percent (60/101) were febrile at presentation, with a median reported duration of fever of 2 days (IQR 2). While none reported antimalarial use in the previous 28 days, 35/101 (35%) had detectable levels of piperaquine in the blood, which was not associated with baseline gametocyte carriage (p = 0.27) [5]. The therapeutic efficacy of DHP was poor in both groups, leading to voluntary study halt prior to reaching target enrollment [5].

**Table 1:** Baseline characteristics of study subjects.

	DHA-PPQ + Primaquine (n = 50)	DHA-PPQ No Primaquine (n = 51)	p-value
Male, no. (%)	48 (96)	50 (98)	0.49
Weight, kg, mean (SD)	57 (8)	58 (6)	0.55
Age, y, median (IQR)	25 (10)	25 (13)	0.49
Occupation, No. (%)			0.86
Farmer	40 (80)	43 (84)	
Forest worker	1 (2)	0	
Military	5 (10)	6 (12)	
Other	4 (8)	2 (4)	
History of malaria in previous year, no. (%)	22 (44)	21 (41)	0.47
Antimalarial use in past 28 days	0	0	—
Fever (≥38.0°C) at presentation, no. (%)	29 (58)	31 (61)	0.78
Duration of fever, days, median (IQR)	2.5 (1.5)	2 (2)	0.46
Hemoglobin, mg/dL, mean (SD)	13.4 (1.8)	13.6 (1.5)	0.46
Parasite density, per μL, mean (95% CI)	14,962 (10,185–21,978)	17,218 (6,389–43,451)	0.60
Gametocyte prevalence by microscopy, no. (%)	4 (8)	5 (10)	0.75
Gametocyte prevalence by RT-PCR*, no. (%)	24 (49)	22 (44)	0.62
Mixed Pf/Pv infection, no. (%)	4 (8)	4 (8)	0.63
G6PD activity**, no. (%)			0.11
Normal	43 (86)	49 (96)	
Partial deficiency	1 (2)	0	
Deficiency	6 (12)	2 (4)	

*denominators are 49 and 50 in the primaquine and no primaquine arms, respectively

**as determined by weak-moderate fluorescence on fluorescent spot testing, indicating intermediate G6PD enzyme activity.

### Infectiousness to mosquitoes

A total of 387 membrane feeding assays were successfully conducted in the 101 subjects pre and post-treatment (n = 101 day 0, n = 100 day 3, n = 96 day 7, n = 90 day 14). Feeding assays were performed a median of 1.0 hours (interquartile range 28 min to 2.2 hours) after venous blood draw. In every experiment, 50 mosquito midguts were dissected 9 days after initial membrane feeding and examined for oocysts. Positive mosquito infection (at least 1/50 with midgut oocysts) was confirmed in all cases by real-time PCR of mosquitoes also saved at day 9 post-feeding.

**Fig 1:**
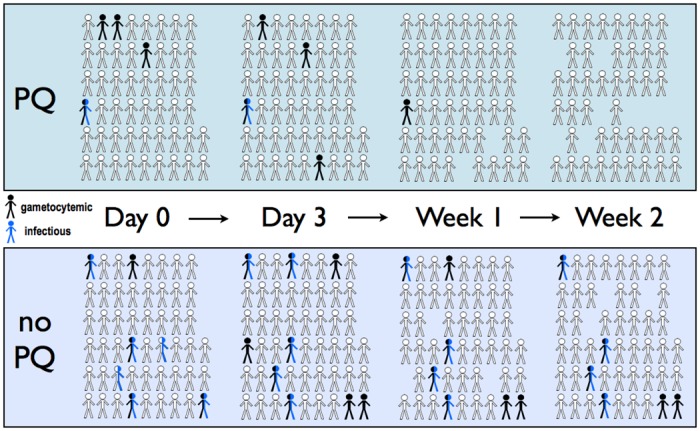
Schematic of gametocyte and mosquito infectivity status through treatment in 101 randomized participants. Participants in the primaquine (PQ) and non-primaquine arms are depicted in the same ordered configuration from Day 0 pre-treatment through Week 2 post-treatment. Subjects with patent gametocytes detected by microscopy are colored black, while subjects who infected at least one mosquito on membrane feeding are colored blue. Persons that were both gametocytemic and infectious are colored half black-half blue. Persons who missed follow-up are shown as missing.

Only 7/101 (6.9%) participants were infectious to mosquitoes pre-treatment—6 in the DHP-only arm and 1 in the DHP+PQ arm [18]. Of the 6 infectious subjects in the DHP-only arm, 4 remained infectious through day 14 post-treatment, and an additional subject who was non-infectious at presentation became infectious on day 3, ~20hrs after primaquine dosing. In contrast, in the DHP+PQ arm, the 1 infectious subject at presentation was rendered non-infectious by day 7, and no others became infectious (Fig 1, Table 2). These small numbers precluded the ability to show a reduction in individual infectiousness due to primaquine. Also, mosquito-feeding assays were not performed on day 2, just prior to PQ dosing, to more directly measure the transmission-blocking effect of primaquine separate from DHP. However, subjects in the non-primaquine arm continued to infect significant numbers of mosquitoes post-treatment (165/2400 or 6.9% at day 7 and 106/2100 or 5.0% at day 14), whereas no mosquito infections occurred in the primaquine arm one and two weeks post-treatment (Table 2).

**Table 2:** Infectiousness to mosquitoes during follow-up.

	DHP + Primaquine			DHP No Primaquine		
	no. of infectious participants	no. of mosquitoes infected/dissected	median no. of oocysts/mosquito (IQR)	no. of infectious participants	no. of mosquitoes infected/dissected	median no. of oocysts/mosquito (IQR)
Day 0	1/50 (2.0%)	35/2500 (1.4%)	42 (26 to 78)	6/51 (12%)	135/2550 (5.3%)	94* (3 to 166)
Day 2	—	—	—	—	—	—
Day 3	1/50 (2.0%)	35/2500 (1.4%)	22 (5 to 36)	5/51 (9.8%)	168/2550 (6.6%)	26 (6 to 68)
Day 7	0/48 (0)	0/2400 (0)	-	4/48 (8.3%)	165/2400 (6.9%)	39 (15 to 93)
Day 14	0/48 (0)	0/2400 (0)	-	4/42 (9.5%)	106/2100 (5.0%)	27 (6 to 57)

*data restricted to 3 of the 6 participants as 1 subject (SN-119) had a mixed Pf/Pv infection and in 2 subjects (SN-063 and SN-086), oocysts were not visualized though multiple PCR-positive mosquito pools suggested these individuals were infectious [18].

Of note, the one gametocytemic and infectious subject in the primaquine group (SN-060) remained infectious at the day 3 membrane feeding, which occurred just ~20hrs after primaquine dosing, but displayed a two-fold reduction in oocyst density in the infected mosquitoes (Fig 1, Table 3). At this 72-hour timepoint, the gametocyte density of 772 gametocytes/μL was comparable to that at baseline (728 gametocytes/μL), having decreased from a peak of 967 gametocytes/μL the first day. The majority of mosquitoes still became infected on day 3 (35/50), but the median oocyst density was 42 (IQR 26 to 78) at day 0 vs. 21 (IQR 5 to 36) at day 3. By day 7, or 5 days post-primaquine, gametocytes were greatly reduced but still present at 71 gametocytes/μL, but these were not infective to mosquitoes.

**Table 3:** Gametocytemia and mosquito infection among infectious patients.

Treatment Arm	Subject ID	Day of followup	Gametocytes/μL	Pfs25 RT-PCR result	Mosquitoes infected	Oocysts/mosquito, median (range)
**DHP + PQ**	SN-060	0	728	+	35/50 (70%)	42 (1–231)
		2	670			
		3	772		35/50 (70%)	21(1–56)
		7	71	**+**	0/50	
		14	0	**-**	0/50	
**DHP only**	SN-002	0	705	**+**	13/50 (26%)	1 (1–12)
		2	569			
		3	427		22/50 (44%)	59 (1–147)
		7	247	**+**	37/50 (74%)	33 (1–120)
		14	161	**+**	6/50 (12%)	5 (1–15)
	SN-010	0	16	**+**	0/50	
		2	87			
		3	71		22/50 (44%)	3 (1–8)
		7	70	**+**	0/50	
		14	0	**-**	0/50	
	SN-063 (M*)	0	690	**+**	26/30 (87%)	
		2	652			
		3	860		26/30 (87%)	
		7	844	**+**	27/30 (90%)	
		14	250	**+**	19/30 (63%)	
	SN-070	0	0	**+**	4/50 (8%)	1 (1–2)
		2	0			
		3	0		0/50	
		7	0	**-**	0/50	
		14	0	**-**	0/50	
	SN-086	0	0	**+**	?**	
		2	47			
		3	77		32/50 (64%)	6 (1–34)
		7	55	**+**	33/50 (66%)	9 (1–27)
		14	54	**+**	40/50 (80%)	12 (1–42)
	SN-108	0	342	**+**	49/50 (98%)	149 (48–398)
		2	412			
		3	375		49/50 (98%)	78 (7–191)
		7	293	**+**	50/50 (100%)	111(32–147)
		14	80	**+**	49/50 (98%)	58 (14–132)
	SN-119 (M*)	0	118	**+**	3/30 (10%)	
		2	145			
		3	96		0/30	
		7	42	**+**	0/30	
		14	28	**-**	0/30	

*Note that for subjects with mixed Pf/Pv infection(M), the percentage of infected mosquitoes was determined by species-specific PCR of individual mosquitoes, and oocyst counts are not reported because of inability to distinguish falciparum vs. vivax oocysts [18].

** Two of 5 and 5 of 5 pools of mosquitoes were real-time PCR positive at days 9 and 16 after feeding, respectively, but oocysts were not seen.

### Gametocyte prevalence and clearance

**Fig 2:**
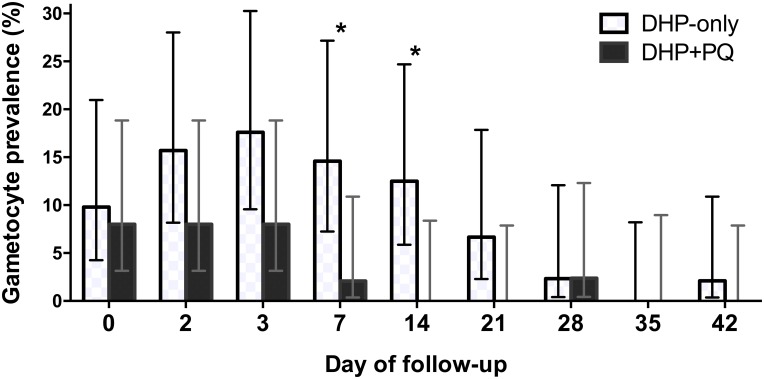
Gametocyte prevalence during 42-day follow-up. Gametocyte prevalence for each regimen, as measured by microscopy. Dihydroartemisinin-piperaquine (DHP) was dosed on days 0–2. Primaquine (PQ) was dosed on day 2. Error bars indicate the upper and lower limits of the 95% CI. *Indicates a statistically significant difference between groups based on a one-tailed Fisher’s exact test.

At baseline, 9/101 (9%) participants were gametocyte positive by microscopy, while 46/101 (46%) were gametocyte positive by Pfs25 RT-PCR. Just prior to primaquine dosing on day 2, the proportion of participants with microscopic gametocytemia increased to 12/101 (12%). By one week post follow-up, 5 days after primaquine dosing, those given primaquine had a lower rate of gametocyte carriage, with 2.1% of subjects in the DHP + PQ group displaying patent gametocytes vs. 15% of those in the DHP-only arm (prevalence ratio 0.12, 95% CI 0.02–0.9, p = 0.03) (Table 4, Fig 2). In the primaquine group, only 1/4 subjects with patent gametocytemia at day 2 remained gametocytemic at day 7, and all were gametocyte-free at day 14. In contrast, the number of subjects with patent gametocytes declined gradually in the non-primaquine group, and gametocyte prevalence did not fall below 10% until 3 weeks post-treatment (Table 4, Fig 2).

**Table 4:** Gametocyte carriage during follow-up.

	DHP + Primaquine		DHP No Primaquine		p-value*	
	by microscopy	by RT-PCR	by microscopy	by RT-PCR	microscopy	RT-PCR
Day 0	4/50 (8.0%)	24/49 (49%)	5/51 (9.8%)	22/50 (44%)		
Day 2	4/50 (8.0%)		8/51 (16%)			
Day 3	4/50 (8.0%)		9/51 (18%)			
Day 7	1/48 (2.1%)	2/47 (4.3%)	7/48 (15%)	11/42 (26%)	0.03	0.003
Day 14	0/42 (0)	0/42 (0)	6/48 (13%)	6/47 (13%)	0.02	0.02

*p-value compares gametocyte prevalence in the two arms post-treatment using one-tailed Fisher's exact test

**Fig 3:**
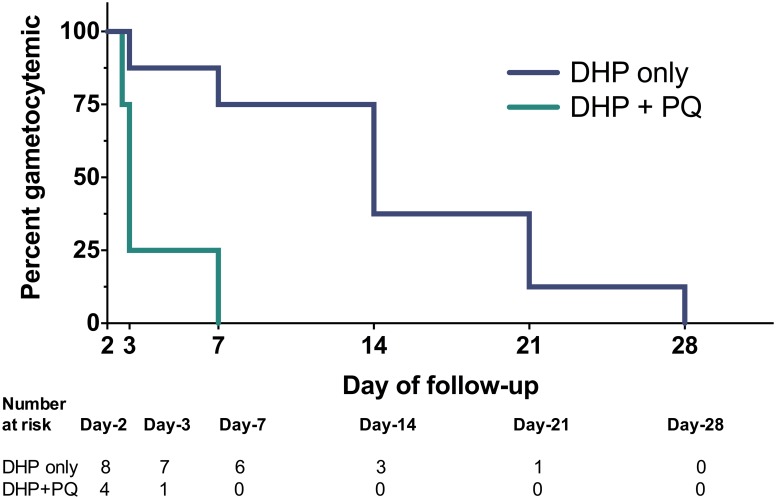
Kaplan-Meier survival curves of gametocyte clearance by treatment regimen. Twelve subjects that were gametocytemic at day 2, just prior to primaquine (PQ) dosing as measured by microscopy, are included. 95% confidence bands are shown.

Accordingly, gametocyte clearance was faster in the primaquine group. Among the 12 participants who were gametocytemic by microscopy at day 2, the median time to clearance was 1 day in the primaquine group vs. 12 days in the non-primaquine group (hazard ratio 7.3 (95% CI 1.3–42), logrank p = 0.01) (Fig 3). The detection of submicroscopic gametocytes further corroborated these findings: gametocytes detected by RT-PCR dropped approximately 10-fold from baseline by day 7 due to the combined effects of DHP and primaquine (49% to 4.3%), but only dropped two-fold from baseline in the non-primaquine group (44% to 26%) (Table 4, S1 Fig).

### Relationship of gametocytemia to infectiousness

**Fig 4:**
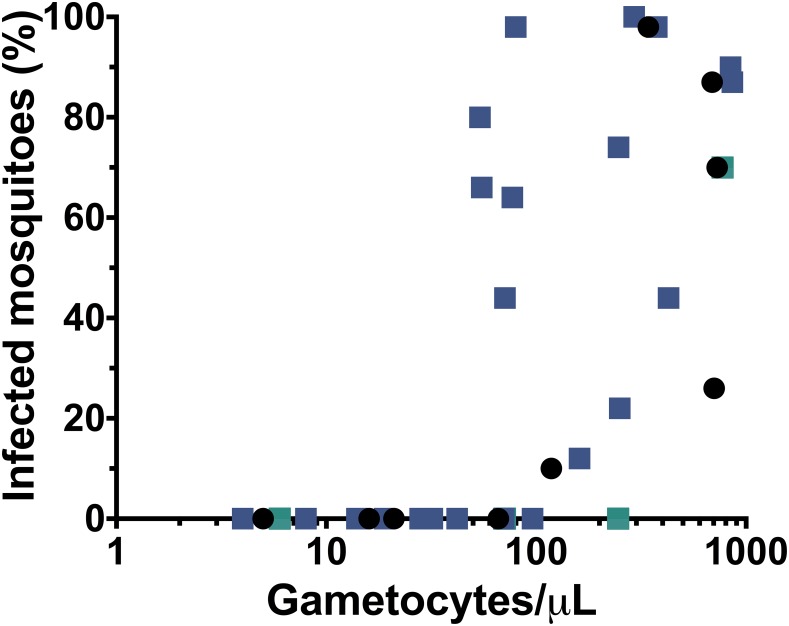
Relationship of microscopic gametocytemia to prevalence of mosquito infection. The results of 35 membrane feeding assays performed on gametocytemic blood from 14 subjects pre and post-treatment. Black circles denote assays performed pre-treatment (Day 0), while colored squares denote assays performed post-treatment (Days 3, 7, 14) on subjects in the DHP-only group (indigo) and subjects in the DHP+PQ group (green). Additionally, the only two mosquito infections observed to arise from submicroscopic gametocytemia (pre-treatment) are depicted on the y-axis. Raw data for infected mosquitoes is available in Table 3. Note pre-treatment data (black circles) were previously presented in Ref 18.

We previously showed a close relationship between microscopic gametocytemia and infectiousness to mosquitoes at baseline pre-treatment in this cohort [18]. Using all the data from serial membrane feedings conducted both pre and post-treatment (N = 387 or roughly 4 feeding assays per subject), microscopic gametocytemia was a good predictor of infectiousness: 19/36, or roughly half, of patent gametocyte episodes led to mosquito infection, while successful transmission was only observed in 2/351 instances when gametocytes were not visible by smear (p<0.001). These two instances of transmission arising from submicroscopic gametocytes caused low-level mosquito infection and occurred pre-treatment (Table 3). Post-treatment, none of the 474 infected mosquitoes found out of 14,350 dissected arose from submicroscopic gametocytes. Despite this difference pre and post-treatment in the infectiousness of submicroscopic gametocytes, we observed no difference in the apparent threshold of microscopic gametocytes (~100 gametocytes/mL) that rendered persons infectious to mosquitoes pre and post-treatment in the absence of primaquine (Fig 4).

### G6PD status and hemolysis

G6PD screening by both qualitative and quantitative tests led to the exclusion of 5 persons with severe G6PD deficiency (WHO Class I or II, less than 10% of normal G6PD activity) prior to enrollment. Of 101 participants enrolled in the study, 8 had mild or moderate G6PD deficiency, with activity ranging from 0.6 to 3.2 U/Hgb (median 1.2 U/Hgb). Among these 8 subjects, a greater drop in hemoglobin at day 7 was seen among those treated with primaquine but was not statistically significant: median fractional reduction in hemoglobin was 24.8% (range 0.9% to 29.8%) in the six subjects given primaquine vs. 7.1% (-4.4% and 18.7%) in the two subjects not given primaquine (p = 0.14). The largest drop was to 9.9 g/dL from a baseline Hgb of 14.1; all Hgb values were >11.0 g/dL by day 14 (S2 Fig). In the G6PD normal subjects, hemoglobin was only measured through day 3, at which time there was no change in the fractional drop in hemoglobin between treatment arms (9.8% vs. 9.3% in the PQ and non-PQ groups, respectively, p = 0.79).

## Discussion

Our study is one of now three randomized control trials using a mosquito infection endpoint to provide evidence for the WHO’s 2010 recommendation to add single dose primaquine to ACT as a transmission-blocking intervention in areas nearing elimination or threatened by artemisinin resistance [14,19]. It is the only trial conducted in Asia and used *Anopheles dirus*, one of the predominant outdoor biting malaria vectors residing in the forest and forest fringes throughout Southeast Asia [20]. It was also conducted in an area of ACT-resistant malaria, where containment has been a global priority. Unfortunately, despite even distribution of microscopic gametocytes between arms and a high rate of submicroscopic gametocytemia overall (~50%), the low rate of infectiousness in the cohort and its uneven distribution between arms led to only one infectious subject in the primaquine arm at baseline vs. six in the non-primaquine arm. Thus, though there was no transmission observed by day 7 among those treated with primaquine, reduction in human to mosquito transmission could not be adequately assessed in the primaquine-treated arm. Despite this limitation, there was a statistically significant reduction in gametocyte carriage due to a 45mg dose of primaquine that paralleled the lack of infectious subjects in the primaquine arm post-treatment.

This study used the single 45mg dose (0.75 mg/kg) of primaquine recommended by WHO at the time it was conducted. The WHO revised their recommendation to use a lower dose of 0.25mg/kg or 15mg in 2012 [21]. The switch to a lower dose was motivated by pooled analysis of prior studies suggesting that the 0.25mg/kg dose would retain transmission-blocking efficacy while avoiding clinically significant hemolysis observed in G6PD-deficient persons at the higher dose, albeit rarely [22–25]. Low dose primaquine offers a significant strategic advantage in most malaria endemic settings where G6PD screening prior to treatment remains impracticable. Given the higher 0.75mg/g dose, we performed universal G6PD screening and excluded 5 volunteers with severe G6PD deficiency (<10% of normal activity). While the higher dose ultimately proved to be safe in 6 subjects with mild to moderate (WHO Class III) G6PD deficiency, prior to recovery at day 14, 3 of the 6 subjects experienced a >25% fractional drop in hemoglobin at day 7 compared to just 2/124, or 1.6% of G6PD-deficient volunteers given the lower 15mg dose in a recent study on the Thai-Myanmar border [26]. Part of this difference may be attributable to the hemolytic effects of symptomatic malaria in our study, absent in the Thai study of healthy asymptomatic volunteers as part of a mass drug administration effort. Still, the results of the Thai-Myanmar study are encouraging. Roughly 1.2% (15/1226) of G6PD-normal volunteers in that study also experienced an asymptomatic >25% reduction in hemoglobin. Another study from Tanzania found mean hemoglobin reductions of roughly 8% among both G6PD deficient and normal volunteers with smear-positive malaria treated with artemether-lumefantrine (AL) and low dose primaquine. Together, this emerging evidence suggests the 025.mg/kg primaquine dose does not increase the risk for clinically significant hemolysis and can be safely given without prior G6PD screening. Ongoing pharmacovigilance studies in Burkina Faso, Mali, and the Gambia are expected to augment this evidence base.

From an efficacy standpoint, dose-finding transmission-blocking studies recently completed in Mali and Burkina Faso now support adequate transmission-blocking efficacy of the lower 0.25mg/kg dose recommended by the WHO [14,19,27] in combination with DHP and AL. It will be useful to replicate these studies in regions outside Africa with different vectors and newer ACT regimens.

As we have previously noted, microscopic gametocytemia was an excellent predictor of infectiousness in our cohort. Of 21 episodes of human to mosquito transmission in the cohort (7 pre-treatment and 14 post-treatment), all but two arose from subjects with microscopic gametocytemia. The two that arose from submicroscopic gametocytes occurred pre-treatment and resulted in low-level mosquito infection (Table 3). Our molecular detection of gametocytes relied on a nested PCR assay that was qualitative, limiting a more quantitative analysis of the gametocyte infectivity relationship at low levels of gametocytes. We also did not attempt to determine the limit of detection of our molecular procedures for oocyst and sporozoite detection. However, they showed good concordance with the results of midgut dissection, suggesting similar levels of detection by these two methods (S1 Table). This is similar to a prior study which used phenol-chloroform DNA extraction from single mosquitoes experimentally infected with low intensity infection and applied a nested PCR also targeting 18srRNA [28].

Perhaps the most unsettling finding here was how much post-treatment gametocytemia still remained in the ACT-alone “control” arm in this setting of prevalent artemisinin and piperaquine resistance. In areas where ACTs have not been compromised, artemisinin-based therapies rapidly clear asexual parasite stages and are thought be effective against immature gametocytes as well, resulting in low rates of post-treatment gametocytemia compared to non-artemisinin drugs [10,11,29]. In a recent meta-analysis of nearly 49,000 patients treated with ACTs, only 1.9% of patients without gametocytemia at enrollment developed patent gametocytemia within 28 days following treatment [30]. In contrast, microscopic gametocytes appeared in 4 patients (approximately 8%) in our ACT-only arm within the first week of therapy. Two of these four patients later developed recrudescent parasitemia. With regards to gametocyte clearance, the same meta-analysis found that over half with patent gametocytes at enrollment were gametocyte-free by day 7, while the median time to clearance in our DHP-only arm was 14 days. These comparisons suggest that as artemisinin-resistant slow-clearing parasites proliferate, gametocyte carriage will increase, as has been noted in some, though not all studies [3,17,31,32]. In our cohort, gametocyte prevalence actually increased post-treatment in the non-primaquine arm, a finding that was not present in a large study in Myanmar in 2008–9 with >800 patients treated with various ACTs, nor in Indonesian patients treated with DHP in 2008–10 [8,33]. We further demonstrate that gametocytes persisting post-treatment remained infectious to mosquitoes, contributing to the reservoir of multidrug resistant malaria (Table 3, S1 Fig). The relative contribution of these previously treated patients to the infectious reservoir (including those receiving suboptimal therapy either in the private sector or through self-treatment) versus the larger pool of untreated asymptomatic persons with submicroscopic malaria remains unknown, yet critically important to shaping malaria elimination strategies [34–36].

Not all ACTs are equal in their ability to reduce gametocytemia. While DHP offers advantages of once daily dosing and a long elimination half-life, its Achilles heel may be more post-treatment gametocyte carriage compared to other ACTs. Two large studies both found that gametocyte carriage rates following DHP treatment were 2–3 fold higher than those following artesunate-mefloquine (ASMQ), the other ACT frequently used in Southeast Asia [8,37], a finding confirmed by meta-analysis [30]. An African study showed that infectivity to mosquitoes was greater in patients treated with DHP vs. artemether-lumefantrine [38]. This may be due to a lower dose of artemisinin in the combination pill compared to other ACTs or a partner drug effect [30]. No matter the reason, the recent policy recommendation to revert to ASMQ in western Cambodia due to escalating DHP failure [39] may prove advantageous from a transmission standpoint. Increasing the number of days that an artemisinin is given, perhaps in a directly observed therapy setting, would also likely have a greater inhibitory effect on developing gametocytes and thus transmission-blocking effect [3,5,40].

The weakness of DHP as an anti-gametocyte ACT is overcome when given with single dose primaquine (8,14,24). Our study shows that this is true even in the setting of high-grade DHP failure, at least with the higher 0.75mg/kg dosing [5]. In our study, a single dose of 45mg primaquine effectively killed pre-existing gametocytes and prevented the development of new gametocytes. Submicroscopic gametocytemia also fell dramatically within 4 days of primaquine administration. The submicroscopic gametocytemia that remains is much less infectious, due to sterilization of gametocytes before they are cleared, as well as a possible bias towards measurement of the more abundant female gametocytes, whereas transmission requires the presence of both male and female gametocytes [12,14,41–43]. Our infectivity data bears this out, as all episodes of mosquito infection after administration of primaquine arose from microscopic gametocytemia.

Overall, our findings, though limited by small numbers and a higher dose of primaquine than currently recommended, lend support to the WHO recommendation to give single dose primaquine to reduce *P*. *falciparum* transmission. They highlight the importance of adding primaquine as a transmission-blocking intervention in areas where ACTs are failing, as we found that Cambodian patients treated with dihydroartemisinin-piperaquine cleared gametocytes slowly and still contributed substantially to the infectious reservoir after treatment. Fortunately, most infectious subjects were readily identified by the presence of patent gametocytes. Despite adoption of primaquine into the official drug policy of many countries, a large gap remains between policy and real-world availability [44]. Overcoming these practice barriers needs to be a priority if primaquine is to be deployed effectively for regional malaria elimination in the Mekong and to mitigate the global spread of multidrug resistant malaria.

## Supporting Information

S1 Fig Schematic of submicroscopic gametocytemia and mosquito infectivity through treatment. This figure is similar to Fig 1, but also shows participants who were gametocyte positive by RT-PCR (in gray). Participants in the primaquine and non-primaquine arms are depicted in the same ordered configuration from Day 0 pre-treatment through Week 2 post-treatment. Subjects with patent gametocytes detected by microscopy are colored black, while those with submicroscopic gametocytes are colored gray, and subjects who infected at least one mosquito on membrane feeding are colored blue. Persons that were both gametocytemic and infectious are colored half black/gray and half blue. Persons who missed follow-up are shown as missing.
(TIFF)

S2 Fig Trends in hemoglobin during follow-up in participants with non-severe G6PD deficiency. Hemoglobin values during follow-up for the 8 participants with G6PD deficiency Class III (>10% enzyme activity) (A) and the calculated fractional change compared to day 0 pre-treatment in those same subjects (B). Trend lines for the subjects in the primaquine group are red (for those with >25% drop in Hgb at day 7) and green (all others). Trend lines for the 2 subjects in the DHP-alone group are black.
(TIFF)

S1 Table Concordance of patient-level infectiousness based on detection of mosquito *P*. *falciparum* infection by microscopy vs. PCR. The tables include membrane-feeding assays conducted on subjects pre and post-treatment. In the upper table, PCR positivity at both Day 9 (oocyst stage) and Day 16 (sporozoite stage) was required for an overall positive determination. In the lower table, only PCR positivity at day 9 was used. In 4 of the 9 discordant results, PCR was positive in 1/5 pools of 10 mosquitoes. In the remaining 5 time points, 2/5 pools were PCR-positive. Subjects with mixed Pf/Pv infections are excluded from this table because of different mosquito processing procedures.
(PDF)

S1 File Study Protocol WRAIR #1877. (PDF)

S2 File Consort 2010 Checklist. (DOCX)

S3 File Study Data. (XLS)
