# Supplementary figures and images for: Single dose primaquine to reduce gametocyte carriage and Plasmodium falciparum transmission in Cambodia: An open-label randomized trial
Source: PLoS One. 2017 Jun 7;12(6):e0168702. doi: 10.1371/journal.pone.0168702 (PMC5462369; doi:10.1371/journal.pone.0168702)

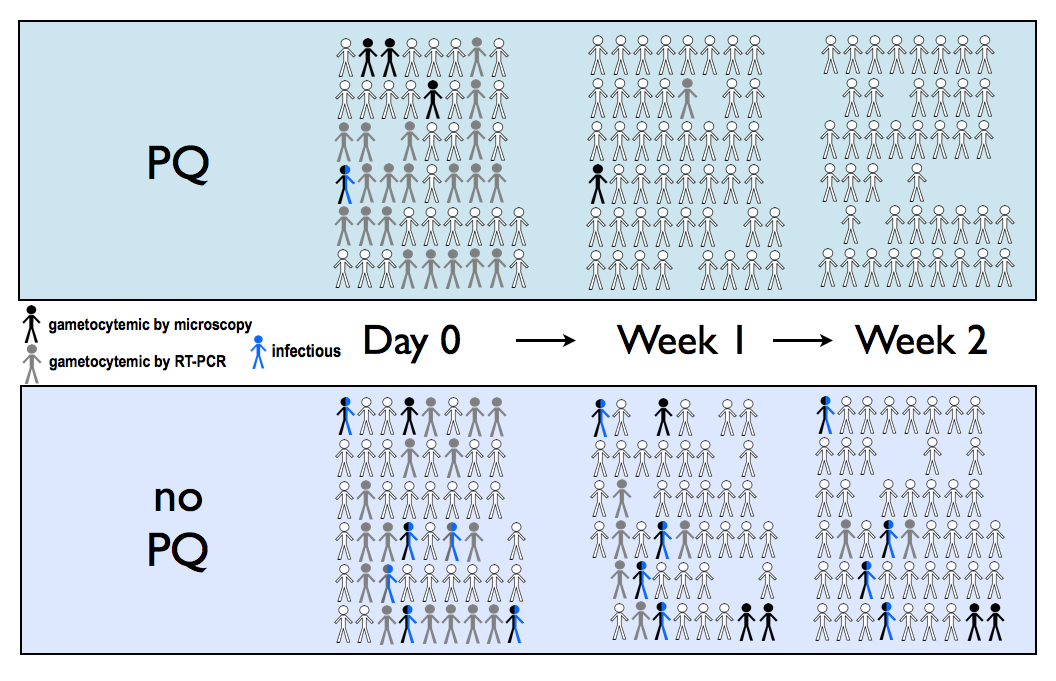

Supplement: S1 Fig — This figure is similar to Fig 1, but also shows participants who were gametocyte positive by RT-PCR (in gray). Participants in the primaquine and non-primaquine arms are depicted in the same ordered configuration from Day 0 pre-treatment through Week 2 post-treatment. Subjects with patent gametocytes detected by microscopy are colored black, while those with submicroscopic gametocytes are colored gray, and subjects who infected at least one mosquito on membrane feeding are colored blue. Persons that were both gametocytemic and infectious are colored half black/gray and half blue. Persons who missed follow-up are shown as missing. (TIFF) [file pone.0168702.s001.tiff]

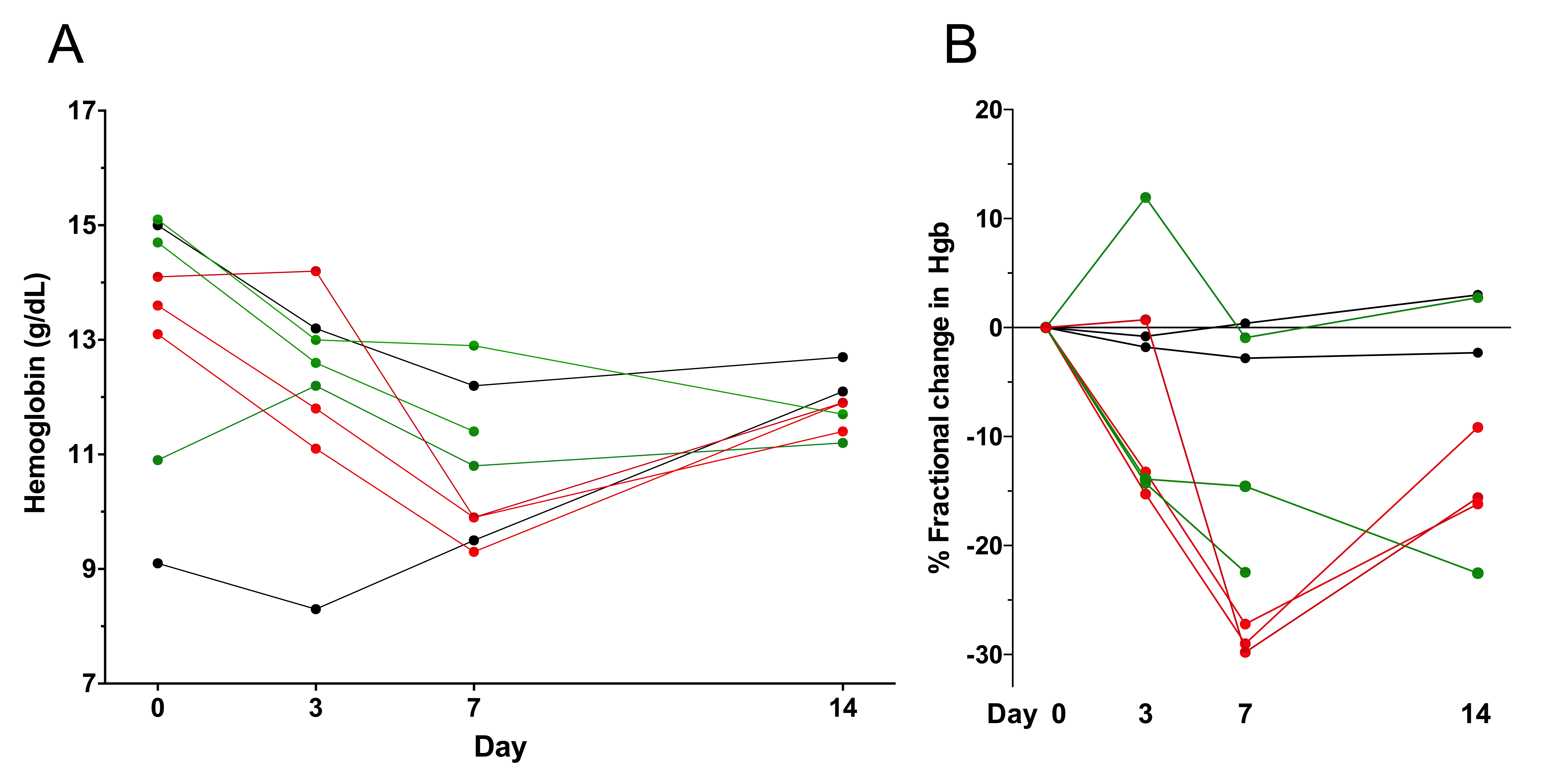

Supplement: S2 Fig — Hemoglobin values during follow-up for the 8 participants with G6PD deficiency Class III (>10% enzyme activity) (A) and the calculated fractional change compared to day 0 pre-treatment in those same subjects (B). Trend lines for the subjects in the primaquine group are red (for those with >25% drop in Hgb at day 7) and green (all others). Trend lines for the 2 subjects in the DHP-alone group are black. (TIFF) [file pone.0168702.s002.tiff]
