# Supplementary material for: Single dose primaquine to reduce gametocyte carriage and Plasmodium falciparum transmission in Cambodia: An open-label randomized trial
Source: PLoS One. 2017 Jun 7;12(6):e0168702. doi: 10.1371/journal.pone.0168702 (PMC5462369; doi:10.1371/journal.pone.0168702)
Supplement: S1 Table — The tables include membrane-feeding assays conducted on subjects pre and post-treatment. In the upper table, PCR positivity at both Day 9 (oocyst stage) and Day 16 (sporozoite stage) was required for an overall positive determination. In the lower table, only PCR positivity at day 9 was used. In 4 of the 9 discordant results, PCR was positive in 1/5 pools of 10 mosquitoes. In the remaining 5 time points, 2/5 pools were PCR-positive. Subjects with mixed Pf/Pv infections are excluded from this table because of different mosquito processing procedures. (PDF) [file pone.0168702.s003.pdf]

		real-time PCR of pooled mosquitoes at day 9 & day 16	
		positive	negative
**oocyst detection in mosquito midgut**	positive	14	0
	negative	2	252

		real-time PCR of pooled mosquitoes at day 9	
		positive	negative
**oocyst detection in mosquito midgut**	positive	14	0
	negative	9	245

Table S1. Concordance of patient-level infectiousness based on detection of mosquito *P. falciparum* infection by microscopy vs. PCR. The tables include membrane-feeding assays conducted on subjects pre and post-treatment. In the upper table, PCR positivity at both Day 9 (oocyst stage) and Day 16 (sporozoite stage) was required for an overall positive determination. In the lower table, only PCR positivity at day 9 was used. In 4 of the 9 discordant results, PCR was positive in 1/5 pools of 10 mosquitoes. In the remaining 5 time points, 2/5 pools were PCR-positive. Mixed Pf/Pv infections are excluded from these tables because of different mosquito processing procedures.
