# Supplementary material for: Single dose primaquine to reduce gametocyte carriage and Plasmodium falciparum transmission in Cambodia: An open-label randomized trial
Source: PLoS One. 2017 Jun 7;12(6):e0168702. doi: 10.1371/journal.pone.0168702 (PMC5462369; doi:10.1371/journal.pone.0168702)
Supplement: S1 File — (PDF) [file pone.0168702.s004.pdf]

Title: Active surveillance for *P. falciparum* drug resistance with assessment of transmission blocking activity of single dose primaquine in Cambodia

WRAIR Protocol Number:
WRAIR # 1877
HRPO Log Number A-17145

Principal Investigators:
LTC David Saunders, USAMC-AFRIMS
Dr. Youry Se, USAMC-AFRIMS

Version Number: *Version 2.3*
7 Sep 2012

CONFIDENTIAL

WRAIR Institutional Review Board (IRB)
Signature Page for Studies Not Exempt Under 32 CFR 219

Principal Investigator Agreement:

1. I agree to follow this protocol version as approved by the IRBs/ERCs.

2. I will conduct the study in accordance with applicable IRB/ERC requirements, Federal regulations, and state and local laws to maintain the protection of the rights and welfare of study participants.

3. I certify that I, and the study staff, have received the requisite training to conduct this research protocol.

4. I will not modify the protocol without first obtaining an IRB/ERC approved amendment and new protocol version unless it is necessary to protect the health and welfare of study participants.

5. (For Greater than Minimal Risk studies or studies of public interest) In accordance with Command Policy 2008-35, I will ensure that the Commanding General receives a pre-brief (or Executive Summary) and approves the study prior to execution.

6. I will ensure that the data (and/or specimens) are maintained in accordance with the data (and/or specimen) disposition outlined in the protocol. Any modifications to this plan should first be reviewed and approved by the applicable IRBs/ERCs.

7. I will promptly report changes to the research or unanticipated problems to the WRAIR IRB immediately via the WRAIR Division of Human Subjects Protection at (301) 319-9940 (during duty hours) or to the WRAIRHSPB@amedd.army.mil and submit a written report within 10 working days of knowledge of the event.

8. I will prepare continuing review reports at an interval established by the IRB/ERC, and a study closure report when all research activities are completed.

9. I will immediately report to the WRAIR Division of Human Subjects Protection knowledge of any pending compliance inspection by any outside governmental agency.

10. I agree to maintain adequate and accurate records in accordance with IRB policies, Federal, state and local laws and regulations.

Dr. Youry Se	Date
Dr. David Saunders	07-Sept-2012<br>Date

WRAIR Institutional Review Board (IRB)
Signature Page for Studies Not Exempt Under 32 CFR 219

Principal Investigator Agreement:

1. I agree to follow this protocol version as approved by the IRBs/ERCs.

2. I will conduct the study in accordance with applicable IRB/ERC requirements, Federal regulations, and state and local laws to maintain the protection of the rights and welfare of study participants.

3. I certify that I, and the study staff, have received the requisite training to conduct this research protocol.

4. I will not modify the protocol without first obtaining an IRB/ERC approved amendment and new protocol version unless it is necessary to protect the health and welfare of study participants.

5. (For Greater than Minimal Risk studies or studies of public interest) In accordance with Command Policy 2008-35, I will ensure that the Commanding General receives a pre-brief (or Executive Summary) and approves the study prior to execution.

6. I will ensure that the data (and/or specimens) are maintained in accordance with the data (and/or specimen) disposition outlined in the protocol. Any modifications to this plan should first be reviewed and approved by the applicable IRBs/ERCs.

7. I will promptly report changes to the research or unanticipated problems to the WRAIR IRB immediately via the WRAIR Division of Human Subjects Protection at (301) 319-9940 (during duty hours) or to the WRAIRHSPB@amedd.army.mil and submit a written report within 10 working days of knowledge of the event.

8. I will prepare continuing review reports at an interval established by the IRB/ERC, and a study closure report when all research activities are completed.

9. I will immediately report to the WRAIR Division of Human Subjects Protection knowledge of any pending compliance inspection by any outside governmental agency.

10. I agree to maintain adequate and accurate records in accordance with IRB policies, Federal, state and local laws and regulations.

Dr. Youry Se	07- SEP- 2012<br>Date
Dr. David Saunders	Date

CONFIDENTIAL

List of Abbreviations

A	Artesunate
ACPR	Adequate Clinical and Parasitologic Response
ACT	Artemisinin-based Combination Therapy
AE	Adverse event
AFRIMS	Armed Forces Research Institute of Medical Sciences
AI	Associate Investigator
CBC	Complete Blood Count
CFR	Common Federal Rule
CNM	National Center for Parasitology, Entomology and Malaria Control
CRADA	Cooperative Research and Development Agreement
CRF	Case Report Form
DHA	Dihydroartemisinin
DoD-GEIS	Department of Defense-Geographic Epidemiologic
DP	Dihydroartemisinin-piperaquine
DNA	Deoxyribonucleic Acid
DOT	Directly Observed Therapy
DP	Dihydroartemisinin-piperaquine
EDTA	Ethylenediaminetetraacetic acid
EKG	electrocardiogram
ELISA	Enzyme-Linked Immunosorbent Assay
EMEA	Europe Medicines Agency
ETF	Early Treatment Failure
FDA	Food and Drug Administration
FWA	Federal-Wide Assurance
GCP	Good Clinical Practice
G6PD	Glucose-6-phosphate dehydrogenase
GMP	Good Manufacturing Practice
GPO	Government Pharmaceutical Organization
HRP	Histidine Rich Protein
HRPO	Human Research Protection Office
HSPB	Human Subjects Protection Branch
IC	Inhibitory Concentration
ICF	Informed Consent Form
ICH	International Conference on Harmonization
IEC	Independent or Institutional Ethics Committee
IND	Investigational New Drug application
IRB	Institutional Review Board
LCF	Late Clinical Failure
LC-MS	Liquid Chromotography-Mass Spectrometry
LD	Linkage Disequilibrium
LPF	Late Parasitologic Failure
M	Mefloquine
MMV	Medicines for Malaria Venture
MR4	Malaria Research and Reference Reagent Resource Center
MSP	Merozoite Surface Protein
MTF	Malaria Treatment Facility
NADPH	Nicotinamide adenine dinucleotide phosphate

CONFIDENTIAL

NDA	New Drug Application
NEHCR	Cambodia National Ethics Committee for Health Research
ORP	Office of Research Protection
QA	Quality Assurance
QC	Quality control
QT_C, QT_F	Correction methods of EKG QT intervals using heart rate
RBC	Red Blood Cell
P.	*Plasmodium*
PCR	Polymerase chain reaction
PCT	Parasite Clearance Time
Pf	*Plasmodium falciparum*
PQ	Primaquine
Pv	*Plasmodium vivax*
RCAF	Royal Cambodian Armed Forces
RNA	Ribonucleic Acid
SAE	Serious Adverse Event
SOP	Standard Operating Procedure
SP	Sulfadoxine-pyrimethamine
SSP	Study Specific Procedure
UIC	Unique Identifier Code
UNC	University of North Carolina
USAMRMC	United States Army Medical Research and Materiel Command
USAMRU-K	United States Army Medical Research Unit-Kenya
US FDA	United State Food and Drug Administration
UV	Ultraviolet
WBC	White Blood Cell
WGA	Whole Genome Analysis
WHO	World Health Organization
WWARN	Worldwide Antimalarial Resistance Network

CONFIDENTIAL

Table of Contents

Appendices 7
1 PROJECT SUMMARY 8
2 KEY ROLES 12
3 BACKGROUND AND RATIONALE 19
3.1 Introduction 19
3.2 Malaria in Cambodia 20
3.2.1 Access, availability and cost of medical care in Cambodia 20
3.2.2 Malaria Epidemiology 20
3.2.3 Trends in artemisinin resistance in Cambodia 21
3.3 Dihydroartemisinin-piperaquine 22
3.4 Primaquine 25
3.4.1 Primaquine in G6PD deficiency 28
3.5 Mosquito infection by membrane feeding 30
4 STUDY OBJECTIVES 32
4.1 Primary Objectives 32
4.2 Secondary Objectives: 32
5 STUDY DESIGN 34
5.1 Overview 34
5.2 Endpoints 34
5.3 Sample Size 36
5.4 Duration of Volunteer Participation 36
5.5 Study Group Descriptions 37
5.6 Population to be Studied 37
5.7 Study Sites and Selection of the Study Population 37
5.7.1 Population Characteristics 37
5.8 Description of Test Article 38
5.8.1 Packaging and Labeling of the Test Articles 38
5.8.2 Storage of Test Articles 38
5.9 Monitoring of Clinical Subject Safety 39
6 METHODS 41
6.1 Recruitment of Study Volunteers 41
6.2 Informed Consent Process 41
6.3 Determination of Eligibility 42
6.3.1 Inclusion Criteria 42
6.3.2 Exclusion Criteria 43
6.4 Screening Procedures 45
6.5 Randomization and Volunteer Assignment 45
6.6 Blinding 46
6.7 Administration of Test Article 46
6.8 Concomitant Medications 47
6.9 Clinical Assessments 47
6.10 Specimen (or Data) Collection and Testing 52
6.10.1 Specimens to be Collected 52
6.10.2 Specimen Preparation, Processing, Handling, and Storage 53
6.10.3 Specimen Labeling and Shipment 57

CONFIDENTIAL

6.10.4 Specimen Storage and Donation for Future Use 57
6.11 Data Management 58
6.11.1 Source Documents 59
6.11.2 Overview of Case Report Forms 59
6.11.3 Data Compilation 60
6.11.4 Disposition of Data 60
6.12 Adverse Events 60
6.12.1 Collecting Adverse Events 61
6.12.2 Documenting Adverse Events 62
6.12.3 Expected Adverse Events 62
6.12.4 Serious Adverse Events and Unanticipated Problems 63
6.12.5 Adverse Event Reporting 64
6.12.6 Follow-up of Adverse Events 65
6.13 Criteria for Discontinuation or Withdrawal of a Subject 66
6.13.1 Halting Rules Criteria 67
6.14 Rescue Treatment for Malaria Infection 67
6.15 Criteria for Study Termination 68
6.16 Quality Control and Quality Assurance 68
7 STATISTICAL METHODS 69
7.1 Statistical Procedures 69
7.1.1 Sample Size Estimation 69
7.1.2 Randomization and Stratification 70
7.1.3 Populations for Analysis 70
7.1.4 Deviations from the Statistical Plan 70
8 DATA ANALYSIS 71
8.1 General Considerations – Data Analysis 71
8.2 Efficacy Study Analysis 71
8.3 Pharmacokinetic Analysis 72
8.4 Parasite drug resistance in vitro 73
8.5 Molecular Markers of Parasite Resistance 73
8.6 Safety Analysis 75
8.7 Interim Analysis 75
9 ETHICAL CONSIDERATIONS 76
9.1 Informed Consent 76
9.2 Volunteer Identification and Confidentiality 76
9.3 Data Management 77
9.4 Risk to Volunteers and Precautions to Minimize Risk 78
9.5 Alternatives to Test Article (or Research Treatment) 80
9.6 Benefits to Volunteers 80
9.7 Risks to Study Personnel and Precautions to Minimize Risk 80
9.8 Risks to the Environment 80
9.9 Financial Incentives to Volunteers 81
9.10 Medical Care for Injury or Illness 81
10 ADMINISTRATIVE PROCEDURES 82
10.1 Institutional Review Board 82
10.2 Protocol Amendments 82

CONFIDENTIAL

Continuing Review 82
10.3 Study Medication Accountability 83
10.4 Disposition of Data 83
10.5 Access to Source Data/Documents 83
10.6 Certification of Translation (where applicable) 83
10.7 Protocol Deviations 83
10.8 Compliance Inspections 84
10.9 Publication Policy 84
10.10 Responsibilities of Study Personnel 84
10.11 Responsibilities of the Research Monitor 86

Appendices

Appendix A: Cambodia National Malaria Program Treatment Guidelines (see attachment)

Appendix B: Dihydroartemisinin-piperaquine package insert (see attachment)

Appendix C: Primaquine package insert (see attachment)

Appendix D: Worldwide Antimalarial Resistance Network (WWARN) Data Management and Statistical Analysis Plan (see attachment)

Appendix E: Recent publicly available information from Sigma-tau pharmaceuticals describing the cardiac safety issues with Eurartesim (DHA-piperaquine) (see attachment)

Appendix F: DSMB Charter

CONFIDENTIAL

1 PROTOCOL SUMMARY

Summary Statement

This is a two-arm, open label **Treatment Study** comparing the efficacy, safety, tolerability and pharmacokinetics of a three-day course of Dihydroartemisinin-Piperaquine (DP) with or without single-dose primaquine in patients with uncomplicated *Plasmodium falciparum* malaria at selected sites of malaria drug-resistance in Cambodia. DHA-piperaquine, soon to be adopted as the first line antimalarial agent by the National Malaria Control Program in Cambodia, will be given as a directly observed, standard three-day fixed dose combination treatment to all volunteers enrolled. The cardiac safety of piperaquine will be monitored with electrocardiograms during the treatment period. On the last day of DP therapy, volunteers will be randomized to receive either a single 45 mg dose of primaquine (PQ) or DP treatment only (no primaquine). Resistance to DP and DP-PQ will be assessed by a combination of clinical, pharmacologic, and parasitologic parameters including genomic signatures of selection during careful weekly follow-up visits for 42 days. Investigators will also be able to evaluate any possible effects of primaquine on the sexual stages of malaria (gametocytes) and potential transmissibility of infection to *Anopheles* mosquitoes as compared to those not treated with primaquine.

Background and Rationale

Plasmodium falciparum malaria (*Pf*) continues to be a major cause of global morbidity and mortality with 350-500 million cases per year, and over 1 million deaths. Despite containment and control efforts, *Pf* continues to be endemic in areas of Cambodia near the Thai, Lao and Vietnamese borders. Multi-drug resistant malaria has been reported recently along the Thai-Cambodian border and has emerged as a significant challenge to malaria control and containment in the region, and as such constitutes a substantial threat to the public health.

Currently the National Center for Parasitology, Entomology and Malaria Control (CNM) recommends a 3-day course of oral artesunate and mefloquine combination therapy for uncomplicated malaria infection caused by *P. falciparum*. However, recent results suggest the efficacy of this combination is declining (Wongsrichanalai, 2008), and that tolerance to the artemisinin component may be a factor (Noedl, 2008). DHA-piperaquine is a safe, well-tolerated drug for the treatment of drug resistant malaria, and has a well documented history of safety and effectiveness, particularly in Southeast Asia. For this reason, it has been adopted as the first line artemisinin-combination therapy (ACT) throughout Cambodia following two years of use as the first line agent in selected containment areas along the Thai border referred to as Zone 1. Monitoring for development of resistance to this combination

CONFIDENTIAL

therapy and loss of clinical or parasitologic effectiveness will be crucial in the assessment and potential adjustments in Cambodian national policy regarding first-line antimalarial usage. The study will be carried out over an estimated three year period, with a goal enrollment of approximately 150 subjects, in order to observe and document any trends in resistance patterns to either DP and/or DP-PQ therapy.

With the global push toward eradication of malaria, renewed focus on elimination of gametocytemia is a key intervention point for the interruption of transmission. The World Health Organization (WHO) currently recommends, for low to moderate transmission areas, a single dose of primaquine (0.75 mg/kg with maximum dose of 45 mg) at completion of therapy for blood stage infection. However, there are few evidenced-based studies assessing this practice. This study aims to evaluate a onetime dose of primaquine in a controlled clinical study to see if it is indeed effective in reducing or eliminating gametocytemia and/or transmissibility to *Anopheles* mosquitoes in Cambodia. This evaluation will be done by detection of circulating gametocytes both by microscopy and PCR at defined time points pre- and post-treatment, as well by assessment of transmissibility of infection using female *Anopheles* mosquitoes. If an additional one time dose is found to be effective, this primaquine “transmission-blocking” therapy will be a useful adjunct to the national malaria program to further reduce the burden of malaria disease.

Objectives

Primary:

1. To monitor therapeutic efficacy (based on rates of recurrence at 42 days) and search for evidence of drug resistance of a fixed-dose 3 day regimen of DHA-piperaquine (DP), with and without a dose of primaquine, in volunteers with uncomplicated *P. falciparum* infection in Cambodia over a 3-year observation period.

2. To establish the transmission blocking (sexual stage) efficacy of the prescribed drug regimen with or without a single oral 45 mg dose of primaquine.

Secondary:

1. To document the safety and tolerability of DHA-piperaquine, including the effect on the electrocardiogram (EKG), particularly the QTc interval, in patients taking 3 day treatment courses of DHA-piperaquine.

2. Assess the degree of antimalarial drug resistance in the parasite populations in Cambodia by correlating 42 day rates of malaria recurrence clinical and pharmacodynamic outcomes (parasite clearance) with pharmacokinetic drug levels, *in vitro* drug susceptibility testing, genomic studies and molecular markers of drug resistance.

3. To quantify the reduction in sexual stage parasites (gametocytes) of the two treatment regimens assessed by 3 methods on volunteers' samples – light microscopy, PCR and a mosquito membrane-feeding assay.

4. Assess the relative contributions of clinical history, baseline immunity levels and parasitologic parameters associated with prior infection on clinical outcome and parasitological responses *in vivo*.

5. To build host nation capacity, with emphasis on training laboratory, clinical and entomological scientists to conduct antimalarial therapeutic efficacy and drug resistance studies.

6. To cryopreserve parasite isolates to standardize antimalarial resistance surveillance monitoring methods *in vitro*.

7. To provide up-to-date antimalarial efficacy data to the Cambodian government, including CNM and Ministry of National Defense, to help determine the appropriate regimens of DHA-piperaquine and primaquine for the treatment of uncomplicated malaria.

8. To harmonize clinical and laboratory approaches to characterizing antimalarial drug resistance between DoD-GEIS overseas labs including, AFRIMS, USAMRU-K, and others.

9. To characterize *P. falciparum* population genetic structure and study the transmissibility of genetic variants to mosquitoes by membrane feeding.

Population

Adults (aged 18 – 65 years old) with uncomplicated *P. falciparum* malaria in the vicinity of sentinel sites along the Thai-Cambodia border.

Study Sites

One or more sites authorized by the Ministry of Health and/or the Ministry of National Defense determined to have high incidence rates of *P. falciparum* malaria based on current estimates by AFRIMS, CNM and the RCAF health services. The study team will be based at two medical treatment facilities (MTF) in Battambang Referral Hospital, Battambang Province, and Along Veng Referral Hospital, Anlong Veng District. Volunteers will be recruited from the surrounding communities.

CONFIDENTIAL

Design and Methodology

This is an active two arm, open-label **Treatment Study** of adults with acute, uncomplicated infection with *P. falciparum* comparing the efficacy (42 day PCR-corrected malaria recurrence rate), safety, tolerability and pharmacokinetics of a three day course of dihydroartemisinin-Piperaquine (DP) with or without a single dose of primaquine. Volunteers with uncomplicated *P. falciparum* malaria or mixed *P. falciparum/vivax* infection will be treated with of a three day course of DP under directly observed inpatient observation at the MTF. The cardiac safety of piperaquine will be monitored with electrocardiograms during the treatment period. On the last day of DP therapy, volunteers will be randomized to receive a single dose of 45mg of primaquine or no primaquine treatment.

Volunteers will be followed weekly thereafter on days 7, 14, 21, 28, 35 and 42 for a brief clinical evaluation and fingerstick for peripheral malaria smear (with PCR assays for genotyping and gametocyte detection) to assess for any development of malaria infection. On days 7 and 14, volunteers will also have blood drawn for piperaquine drug level (with *Pf* bioassay) and mosquito membrane feeding to assess the effect of DP +/- primaquine on gametocyte transmissibility. See Section 6 for detailed outline of blood draws.

Any volunteers with recurrent malaria symptoms during the 42-day follow-up period will be re-evaluated by microscopy, and if positive for malaria, will be treated under directly observed therapy based on current national malaria treatment guidelines for Cambodia. Parasites will be collected for *in vitro* drug susceptibility characterization and molecular markers of resistance, along with a piperaquine drug level.

Study Duration

Individual participation is expected to last 42 days from enrollment. Volunteers that have a malaria recurrence during the 42 day treatment follow-up period will be re-treated under national guidelines and be followed for the remainder of the 42-day period. If malaria infection develops after Day 35 of the study, the duration of participation for volunteers may be extended one to two weeks if necessary to ensure blood stage parasite clearance and clinical cure.

The study is expected to run for up to 3 years, to enroll at least 150 evaluable volunteers. Depending on observations made during this time, the study could be extended or ended early, in consultation with the responsible ethical review boards. In addition, it is possible that recommended drug regimens may change over time, depending on shifts in policy and/or drug availability in Cambodia.

CONFIDENTIAL

2 KEY ROLES

Principal Investigators:

Youry Se, M.D., M.P.H.M.
Armed Forces Research Institute of Medical Sciences (AFRIMS)
No. 18, Ste 118
LIM Apartment,
Phnom Penh, Cambodia
Tel: 855 23 881 845 or 855 012 992 029
Email: yourys@afrims.org

David Saunders, M.D., M.P.H.
LTC, MC, USA
Dept. of Immunology and Medicine
Armed Forces Research Institute of Medical Sciences (AFRIMS)
315/6 Rajvithi Road, Bangkok 10400, Thailand
Tel. 66-2-696-2798, Fax 66-2-644-4784
Email: david.saunders@afrims.org

Clinical Investigators:

Michele Spring, M.D., M.S.P.H
Dept. of Immunology and Medicine
Armed Forces Research Institute of Medical Sciences (AFRIMS)
315/6 Rajvithi Road, Bangkok 10400, Thailand
Tel. 66-2-696-2700 ext 4624, Fax 66-2-644-4784
Email: michele.spring@afrims.org

Prom Satharath, M.D.
Department of Health,
Ministry of National Defense
Russian Blvd, Phnom Penh, Cambodia
Tel: 855 12 835 565
Email: satharathprom@yahoo.com

So Mary, M.D.
Chief of Military Health Officer of Regional 4
Kandek commune, Srong Basat Bakorng,
Siam Reap Province, Cambodia
Tel: 855 97 966 6111
Email: mary_mr4@yahoo.com

Sok Somethy M.A.
Department of Health,
Ministry of National Defense
Russian Blvd, Phnom Penh, Cambodia
Tel: 855 12 977 501
Email: soksomethy@ymail.com

CONFIDENTIAL

Chanthap Lon, M.D., M.C.T.M.
Armed Forces Research Institute of Medical Sciences (AFRIMS)
No. 18.Ste 118
LIM Apartment
Phnom Penh, Cambodia
Tel: 855 23 881 845 or 855 12 976799
Email: chanthapl@afrims.org

Delia Bethell, B.M. BCh, M.A.
Dept. of Immunology and Medicine
Armed Forces Research Institute of Medical Sciences (AFRIMS)
315/6 Rajvithi Road, Bangkok 10400, Thailand
Tel. 66-2-696-2791, Fax 66-2-644-4784
Email: delia.bethell@afrims.org

Sut Thangphann, M.D.
National Center for Parasitology, Entomology and Malaria Control
Office: #372, Monivong Blvd.
Phnom Penh, Cambodia
Tel.: 855 23 211 926,
Fax: 855 23 211 926
Email: sut_thangphann@yahoo.com

Jongsakul Krisada, M.D.,
Dept. of Immunology and Medicine
Armed Forces Research Institute of Medical Sciences (AFRIMS)
315/6 Rajvithi Road, Bangkok 10400, Thailand
Tel. 66-2-696-2700, Fax 66-2-644-4784
Email: Krisadaj@afrims.org

Clinical Research Coordinator:

Mali Ittiverakul RN, Clinical Research Coordinator
Dept. of Immunology and Medicine
Armed Forces Research Institute of Medical Sciences (AFRIMS)
315/6 Rajvithi Road, Bangkok 10400, Thailand
Tel. 66-2-696-2791,   Fax 66-2-644-4784
Email: Malii@afrims.org

Soklyda Chann RN
Armed Forces Research Institute of Medical Sciences (AFRIMS)
#18 118St. Meataphea, Phnom Penh, Cambodia
Cell Phone: +855 12 662 751
Email: soklyda.chann@afrims.org

CONFIDENTIAL

Laboratory Investigators:

Charlotte Lanteri, PhD.
Dept. of Immunology and Medicine
Armed Forces Research Institute of Medical Sciences (AFRIMS)
315/6 Rajvithi Road, Bangkok 10400, Thailand
Tel. 66-2-696-2796, Fax 66-2-644-4784
Email: charlotte.lantieri@afrims.org

Paktiya Teja-isavadharm, PhD.
Dept. of Immunology and Medicine
Armed Forces Research Institute of Medical Sciences (AFRIMS)
315/6 Rajvithi Road, Bangkok 10400, Thailand
Tel. 66-2-696-2795, Fax 66-2-644-4784
Email: paktiyat@afrims.org

Pattaraporn, Vanachayangkul PhD
Dept. of Immunology and Medicine
Armed Forces Research Institute of Medical Sciences (AFRIMS)
315/6 Rajvithi Road, Bangkok 10400, Thailand
Tel. 66-2-696-2791, Fax 66-2-644-4784
Email: pattarapornv@afrims.org

Ratawan Ubalee, PhD
Chief, Malariology Section
Dept. of Entomology.
Armed Forces Research Institute of Medical Sciences (AFRIMS)
315/6 Rajvithi Road, Bangkok 10400, Thailand
Tel. 66-2-696-2818, Fax 66-2-354-7885
Email: ratawanu@afrims.org

Alongkot Ponlawat, PhD.
Chief, Vector Biology and Control Section
Dept. of Entomology.
Armed Forces Research Institute of Medical Sciences (AFRIMS)
315/6 Rajvithi Road, Bangkok 10400, Thailand
Tel. 66-2-696-2816, Fax 66-2-354-7885
Email: alongkotp@afrims.org

CONFIDENTIAL

Suwanna Chaorattanakawee, PhD
Dept. of Immunology and Medicine
Armed Forces Research Institute of Medical Sciences (AFRIMS)
315/6 Rajvithi Road, Bangkok 10400, Thailand
Tel. 66-2-696-2791, Fax 66-2-644-4784
Email: suwannac@afrims.org

Sathit Pichyangkul, PhD.
Dept. of Immunology and Medicine
Armed Forces Research Institute of Medical Sciences (AFRIMS)
315/6 Rajvithi Road, Bangkok 10400, Thailand
Tel. 66-2-696-2791, Fax 66-2-644-4784
Email: sathitp@afrims.org

Panita Gosi, PhD.
Dept. of Immunology and Medicine
Armed Forces Research Institute of Medical Sciences (AFRIMS)
315/6 Rajvithi Road, Bangkok 10400, Thailand
Tel. 66-2-696-2791, Fax 66-2-644-4784
Email: panitag@afrims.org

Steven R Meshnick, MD, PhD
Jonathan Juliano, MD
Jessica Lin, MD
Department Of Epidemiology, CB#7435
Gillings School of Global Public Health
UNC School of Medicine
Chapel Hill, NC 27599

Source of funding:

Global Emerging Infections Surveillance and Response System (GEIS)

Research Monitor:

Koy Lenin, M.D.
Battambang Referral Hospital
Emergency and ICU unit
Dongkorteap village, Tuol Ta Ek Commun,
Battambang District, Battambang Province
Tel: 855 53 730 100
Fax: 855 53 953 223

Kong Phan, M.A.
ICU, Anlong Veng Referral Hospital
Anlong Veng Commune, Anlong Veng District,
Oddormean Chey Province, Cambodia
Tel: 855 976 476 188

CONFIDENTIAL

Laboratory Coordinators:

Worachet Kuntawunginn
Laboratory Quality Assurance
Dept. of Immunology and Medicine
Armed Forces Research Institute of Medical Sciences (AFRIMS)
315/6 Rajvithi Road, Bangkok 10400, Thailand
Tel. 66-2-696-2791, Fax 66-2-644-4784
Email: worachetk@afrims.org

Montri Arsanok
Medical Technologist
Dept. of Immunology and Medicine
Armed Forces Research Institute of Medical Sciences (AFRIMS)
315/6 Rajvithi Road, Bangkok 10400, Thailand
Tel. 66-2-696-2791, Fax 66-2-644-4784
Email: montria@afrims.org

Consultants:

Char Meng Chuor, MD, MPH, Director
Keng Sim, MD, Deputy Director
National Center for Parasitology, Entomology and Malaria Control
Office: #372, Monivong Blvd. Corner St. 322
Phnom Penh, Cambodia
Tel.: 855 12 926 266, Fax: 855 12 841 168
Email: mengchuor@cnm.gov.kh

Lieutenant General Kong Saly
Major Deth Vantha
Department of Health
Ministry of National Defense
Russian Blvd, Phnom Penh, Cambodia
Tel: 855 12 835 565

Mark Fukuda, COL, MC
Armed Forces Health Surveillance Center
11800 Tech Rd
Silver Spring, MD 20904, USA
Tel.: 301-319-3289 Fax: 301-319-9213
Email: Mark.Fukuda@us.army.mil

Douglas Walsh, M.D.
Dept. of Dermatology
Charlie Norwood Veterans Affairs Medical Center
950 15th St Augusta, GA 30901
Email: dsw518@yahoo.com

CONFIDENTIAL

Institutions engaged in Human Subjects Research:

National Center for Parasitology, Entomology and Malaria Control
#372, Monivong Blvd., Phnom Penh, Cambodia
Tel.: 855 23 211 926; Fax 855 23 996 202
(FWA# 00010451)

Ministry of National Defense, Royal Cambodian Armed Forces
Department of Health,
Ministry of National Defense
Russian Blvd, Phnom Penh, Cambodia
Tel: 855 12 835 565
(FWA# 00014798)

Armed Forces Research Institute of Medical Sciences (AFRIMS)
Department of Immunology and Medicine
315/6 Rajvithi Road, Bangkok 10400, Thailand
Tel. 66-2-696-2791; Fax 66-2-644-4784
(FWA# 00000015)

Laboratories:

Armed Forces Research Institute of Medical Sciences (AFRIMS)
Dept. of Immunology and Medicine
315/6 Rajvithi Road, Bangkok 10400, Thailand
Tel. 66-2-696-2791; Fax 66-2-644-4784

AFRIMS/CNM Reference Laboratory
Battambang Referral Hospital
Dongkorteap Village, Tuol Ta Ek Commun,
Battambang District, Battambang Province, Cambodia
Tel: +855 92 666 959

AFRIMS/CNM Field Laboratory
Anlong Veng Referral Hospital
Anlong Veng, Oddormean Cheay Province, Cambodia
Tel: +855 99 230 488

University of North Carolina
c/o Steven Meshnick
3113 Hooker Research Building
135 Dauer Dr.
Chapel Hill, NC 27599

CONFIDENTIAL

Institutional Review Boards:

WRAIR IRB (FWA# 00000015, IRB#00000794)
Walter Reed Army Institute of Research
Human Subjects Protection Branch 503 Robert Grant Ave., Silver Spring, MD 20910-7500
Tel.: 301-319-9940
Fax: 301-319-9961
Email: wrairhspb@amedd.army.mil

National Ethics Committee for Health Research (NECHR)
Hlth IRB# 1 (FWA# 00010451, IRB # 00003143)
#2 Kim Il Sung Blvd, Khan Tuol Kok, Phnom Penh. Cambodia
Tel.: 855 23 880-345, Fax: 855 23 880-346
E-mail: research03@nchads.org.

University of North Carolina (NHSR Determination)
Office of Human Research Ethics (FWA# 00004801, IRB # 00000540)
Medical School Building 52
Mason Farm Road, CB 7097
Chapel Hill, NC 27599
Tel.: (919) 966-3113
ohre.unc.edu

CONFIDENTIAL

3 BACKGROUND AND RATIONALE

3.1 Introduction

The US military is charged with protecting personnel deployed to malarious areas, and must be prepared for potential world-wide deployments on short notice. In addition to public health measures to protect and sustain the force, the US military has invested substantially in products to protect the warfighter from malaria at considerable cost. Despite these investments, efficacy is threatened by multi-drug resistant malaria parasites with increasing resistance to remaining effective drug classes becoming more apparent. The development of a “next-generation” malaria chemoprophylaxis agent has long been an identified requirement for advanced clinical development by the US Army. However, any development candidate is threatened by the possibility of antimalarial drug resistance. Therefore, accurate, timely and relevant data on antimalarial drug resistance, in part as a predictor of resistance patterns likely to emerge in the near future are of critical importance to military planners.

Multidrug resistance is a significant problem in many regions of the world, where most strains of *P. falciparum* are no longer susceptible to the available anti-malarial compounds. This problem has been well documented in Southeast Asia, and it is predicted that a similar situation will occur in Africa (Wongsrichanalai, 2001 and 2002; Hyde, 2002). Chloroquine, once the first-line defense against malaria in Kenya, is no longer effective because of the evolution of multidrug resistant parasites (Price, 2001; Bloland, 1993; Shretta, 2000). Chloroquine replaced sulfadoxine-pyrimethamine as the drug of choice, but parasite resistance to this treatment developed quickly as well (Omar, 2001; Mberu, 2000; Khan, 1997). As a result, new candidates for antimalarial chemoprophylaxis and treatment to protect the deployed warfighter are being developed by the US Army with a view to preventing the development of resistance. Cambodia has been particularly hard-hit by drug resistance with many drugs having fallen to resistance over recent years including chloroquine, mefloquine, sulfadoxine-pyramethamine, and now some evidence of artemisinin resistance. AFRIMS has an existing research team on the ground which has been actively conducting malaria resistance research in Cambodia for the past 6 years in partnership with the Cambodian National Center for Parasitology, Entomology and Malaria Control (CNM) and the Royal Cambodian Armed Forces (RCAF). This study aims to monitor and gather information regarding the continued effectiveness of an artemisinin-based antimalarial regimen in areas of known drug resistance in Cambodia as well as assess the effectiveness of primaquine as a transmission blocking therapy.

CONFIDENTIAL

3.2 Malaria in Cambodia

3.2.1 Access, availability and cost of medical care in Cambodia

Overall, health outcomes have been gradually improving in Cambodia over the past 5 years. The infant mortality rate decreased from 95 per 1,000 live births in 2000 to 66 per 1,000 live births in 2005, and continues a steady decline. For pregnant women antenatal health checks, with tetanus immunization and iron supplements if indicated, are offered monthly free of charge from 7 months of pregnancy. In Battambang and neighboring provinces the proportion of women delivering at a health care facility is 36%. The government expenditure on healthcare per capita is roughly $4 (Ministry of Health, 2006). Despite significant progress, the health status of the Cambodian people is still among the lowest in the region.

Access to medical care has gradually improved for individuals at highest risk for malaria, including those who are living far from the health facility, such as traditional forest inhabitants, temporary forest migrants, and new forest settlers. In general, public health centers provide basic medical care for a small charge. There are small use charges - e.g., $0.12 charge per visit for the out-patient department and a $5 charge for in-patient care. Particularly poor patients may be treated free of charge based on means testing. Neither private nor government health insurance is generally available, particularly in rural areas.

3.2.2 Malaria Epidemiology

Malaria typically occurs in seasonal peaks in Cambodia. While malaria transmission is reported to have declined dramatically in Battambang Province (where AFRIMS has conducted field studies in the past), rates of uncomplicated malaria remain high in Oddor Meanchey. From September to December 2009, AFRIMS collected samples from 214 smear-positive malaria cases Oddor Meanchey Province under protocol WR 1576. The species distribution of these infections was 60% *P. falciparum*, 37% *P. vivax*, and 4% mixed infections. More than 50% of cases were detected through active surveillance of fever cases by community outreach. Of 531 cases of fever evaluated, 24.9% had malaria parasites with 51 *P. falciparum* cases and 78 *P. vivax* cases.

Data from the Anlong Veng health center in Oddor Meanchey Province in 2008 indicate two peak seasons: the first in June – August, with a second peak October – December. In 2008, there were 676 cases of uncomplicated malaria reported from this health center alone: 54% *P. falciparum*, 38% *P. vivax*, and 8% mixed infection. This suggests a population with mixed immunity levels and subgroups with high rates of subclinical infection, as well as a large number of symptomatic individuals presenting to healthcare facilities. In addition, there were 284 severe malaria cases requiring admission for parenteral therapy, and seven in-hospital deaths were reported, suggesting that interventions to improve access to early diagnosis and treatment of malaria remain priorities for this region. Several factors appear to have

CONFIDENTIAL

contributed to the relatively higher mortality rates among patients who were referred to government hospitals including delayed presentation, delayed admission referral, cultural beliefs, and difficulty accessing health care facilities in this austere setting. The key to preventing mortality remains early detection, appropriate and effective anti-malarial treatment and referral where necessary.

Little has been reported in the peer reviewed literature about the burden of severe malaria in Cambodia. Recently AFRIMS conducted a study of severe malaria at Battambang Referral Hospital 2006-2009 (unpublished). A total of 537 cases were discharged from BRH with a diagnosis of severe malaria infection over the 3.5 year period. Overall mortality was 14%. Two hundred thirty three cases (43.4%) were documented P. *falciparum* infection; 41 (7.6%) were P. *vivax*; 17 (3.2%) were mixed infection with P. *falciparum* and P. *vivax* and 246 (45.8%) were diagnosed as malaria infection but were slide-negative or a slide was not read and/or reported. Of the 246 smear negative clinical diagnoses, 126 (51.2%) were determined by the investigators to be otherwise compatible with a severe malaria diagnosis under national guidelines after reviewing co-morbidities and hospital course. Among the slide-negative patients, 106 (43.1%) were treated with anti-malarial drugs alone, and another 140 (56.9%) were given combined treatment with an anti-malarial drug and an antibiotic due to suspicion of bacterial co-infection.

3.2.3 Trends in artemisinin resistance in Cambodia

AFRIMS recent work in malaria drug resistance began on the Thai side of the border in Trat Province in 2006 when it was found that cure rate with the then standard 2-day artesunate plus mefloquine (A + M) course was only 78.6% (Vijaykadga *et al*., 2006). In follow-up to this work, AFRIMS designed a study to compare 2- vs. 3-day A+M in Trat, but due to declining transmission of *P. falciparum* in that area, investigators were able to enroll only 13 subjects. However, there were three treatment failures out of the six volunteers enrolled in the 2-day A + M treatment group (Bethell, unpublished) suggesting that resistance was indeed evolving.

This work was followed up in 2007 in the nearby border area of Tasahn, Cambodia. Ninety subjects with uncomplicated *P. falciparum* were enrolled and randomized in 2:1 allocation to 7 days of artesunate monotherapy (AS) at 4 mg/kg or 7 days of quinine and tetracycline (Q-T) with 28 day follow-up in a non-transmission area (Noedl, 2008). The adequate clinical and parasitological response (ACPR) was similar for both drugs: 94% for AS vs. 100% for Q-T. Artesunate did demonstrate statistically significant shorter median parasite clearance times (PCTs) (57.6 hours versus 77.5 hours, p=0.004) as well as better tolerability. There were two subjects receiving AS who had recrudescences despite what were considered to be adequate plasma drug levels with IC_{50} approximately four times higher against DHA than those volunteers who were cured. This study was followed up by a more detailed study in 2009 at the same site where 134 evaluable subjects with *P. falciparum* were enrolled to 2, 4 or 6 mg/kg AS monotherapy in 2:1:2 treatment allocation. Despite overall increased mean parasite clearance times compared to the 2007 study, there were

CONFIDENTIAL

no significant differences in ACPR or treatment failures among the three groups, nor were there clear correlations between clinical outcomes and plasma pharmacokinetic drug levels or in vitro parasite drug resistance profiles (Bethell 2011). Of note, the 6 mg/kg dosing arm had to be halted after five of the 25 enrolled subjects developed non-clinically significant neutropenia, an apparent dose-limiting toxicity. Despite great variability in drug resistance metrics, it was concluded that the increase in PCTs over 3 years, combined with elevated IC_{50}s against DHA, was a sign of emerging resistance, and that ongoing surveillance was required.

As of 2011, there are few if any agents to fill the gap should the current antimalarials in use in Cambodia fall to resistance, and a vaccine is not likely to be available in the clinic for at least several years. Therefore, a combination of rational drug use with ongoing monitoring for resistance patterns must be pursued. This latter point can be achieved by monitoring for any fall in efficacy below 90% using a standard 42 day efficacy protocol as recommended by WHO, and if such a decline is detected, this is generally considered to be indicative of a need to switch to a new agent. Detecting and quantifying drug resistance in its early stages requires a combined *in vivo - in vitro* strategy. The generally accepted approach is a careful analysis of clinical treatment response parameters combined with in vitro drug sensitivity data. In addition, this study hopes to gather evidence for the potential usefulness of adding a single dose regimen of primaquine to interrupt malaria transmission by eliminating circulating gametocytes. This then is the basis for the current proposal for a 42-day efficacy study to a standard 3-day course of DP or DP/PQ treatment for acute *P. falciparum* infection in Cambodia.

3.3 Dihydroartemisinin-piperaquine

Dihydroartemisinin-piperaquine (DP) is a combination of a potent, rapid acting artemisinin derivative, combined with a long-acting 4-aminoquinoline (bis-quinoline), similar to chloroquine. Dihydroartemisinin (DHA) is the active metabolite of artesunate and artemether. Piperaquine is highly active against chloroquine-resistant *Plasmodium falciparum*, and *vivax* (Hung, 2004) and has a terminal half-life of several weeks (Tarning, 2008). Between 2003 and 2006, clinical trials on the safety and efficacy of DP in against *P.falciparum* and *P. vivax* malaria were carried out in several countries: Thailand, Myanmar, Laos and Cambodia, Uganda, Rwanda (Zwang, 2009). In all trials, follow-up was at least 28 days and new infections were distinguished from recrudescences by PCR correction. In this pooled analysis of more than 3,547 uncomplicated malaria patients (1,814 on DP), DP was safe and highly effective. DP administered as treatment was well tolerated with less adverse events in children and adults compared to a 3-day regimen of mefloquine and artesunate with the exception of diarrhea. DP treatment resulted in a rapid clearance of fever and parasitemia with a cumulative PCR-corrected efficacy at Day 28 of 98.7% (95% CI 97.6–99.8). DP was superior to the comparator drugs in protecting against both *P. falciparum* recurrence and recrudescence. There was no difference between DP and Artesunate + Mefloquine for 3 days in treating *P. vivax* co-infections and in suppressing the first relapse. This suggests that DHA-

CONFIDENTIAL

piperaquine could serve as a highly effective antimalarial therapy, particularly in settings of drug resistance where combination therapy is desirable (Jansenns, 2007; Zwang, 2009).

DP has been studied in endemic areas of Cambodia. A randomized open-label non-inferiority study comparing the efficacy of 3 days of DP to 3 days of artesunate and mefloquine (A+M) in 464 Cambodian patients found that PCR-adjusted cure rates on day 63 were nearly identical at 97.5% for both DP and A+M (Janssens, 2007). DP was better tolerated; vomiting, dizziness, palpitations, and sleep disorders were all more commonly reported in the A+M group, consistent with the side-effect profile of mefloquine. In 2010, USAMC-AFRIMS, CNM and RCAF conducted a malaria treatment study (WR 1737) comparing 2 versus 3 days of DHA-piperaquine although administering the same cumulative treatment dose currently recommended by WHO (360mg/2880mg). A total of 80 subjects were enrolled. The study found that there were no differences in DHA-piperaquine efficacy with rates of malaria recurrence at 42 days being very similar in both groups: 89% per protocol efficacy for 2 days of DP (95% CI = 76-96%) and 92% for 3 days of DP (95% CI = 80-97%). Only 2 cases (2.5%) recurred within 30 days of treatment.

A formulation of DP known as Eurartesim® (dihydroartemisinin-piperaquine), has been submitted to the EMEA on 2 July 2009 for regulatory approval by Sigma-tau Italy and MMV. An NDA is also likely to be submitted to the U.S. FDA. DP has been found to be highly effective against *P. falciparum* malaria in adults and children, has a simple dosing regimen (only 3 administrations over 3 days) compared to artemether lumefantrine (Coartem) – the current global standard ACT. In addition, DP has been shown to offer greater protection against new infections than other ACTs, for at least 2 months after treatment. The regulatory dossier submitted comprises data from large clinical trials that involved over 2,700 patients in Africa and Asia of whom 1,600 were children under 5 (MMV press release, July 2009). The US Army has engaged in preliminary discussion with Sigma-tau regarding a possible development partnership, but no formal agreement exists at this time. While the Eurartesim product has not yet been granted EMEA licensing approval as of writing, several non-GMP forms of this combination product are available from manufacturers in China. Duo-cotecxin and Artekin are brand names for two of the products that are available in Cambodia, and this study will use the former for malaria treatment.

Confidential data from the Investigator's Brochure of DHA-piperaquine provided to the US Army by Sigma-Tau did reveal that QTc prolongation was seen at treatment doses in two large Phase 3 studies conducted in Asia and Africa although these increases were mild and transient. EKGs on days 0 (pre-dose), 2 and 7 were obtained on roughly 1000 subjects dosed either with DHA-piperaquine or a comparator ACT drug for treatment of uncomplicated malaria. In the Asian study (ST3073+ST3074 DM040010), there was a statistically significant increase in the proportion of patients with borderline and prolonged QTcB and QTcF values in the DP versus the Artesunate + Mefloquine group, but by Day 7, there was no difference

CONFIDENTIAL

between treatments. In the African Study (ST3073+ST3074 DM040011), there was a highly statistically significant difference on day 2 between treatments in QTcB but not QTcF prolongation, with a higher proportion of patients in the DP group having borderline or prolonged QTcB intervals than in the artemether-lumefantrine group. This had also resolved by Day 7.

Mytton et al (2007) published the results of two clinical trials evaluating QT prolongation following DHA-piperaquine therapy that found minimal QT prolongation indistinguishable from that attributable to malaria itself during and shortly after dosing over 48 hours. Further, the QT prolongations observed could not be distinguished from previously documented QT interval changes reported for other antimalarials without QT-prolonging properties. In the recently updated guidelines from WHO (2010) on treatment of malaria, DP was added to the list of first-line ACTs based on results from head-to-head drug studies conducted with alternative ACT regimens. Regarding potential cardiac adverse events, the guidelines state “There have been reports of…bradycardia and prolongation of the QT interval, although most studies have not found any electrocardiogrpahic abnormalities.”

In AFRIMS WR 1737 evaluating 2- vs. 3-day DP dosing, the effect on the cardiac QT interval was studied intensively. EKGs were obtained at screening, pre-dose, daily for 3 days, and then weekly for 4 weeks if prolongations were seen during the dosing period. Mean QTcB increased only 5-6%, or 6-7% by QTcF, over baseline following dosing, and the treatment groups were essentially indistinguishable in terms of adverse events. Only 2 out of 80 volunteers had a prolongation greater than 20% over baseline by QTcB or QTcF, and in both cases, this was observed on a single day during a 6 week follow-up period. Of note, one volunteer had greater than 500ms prolongation by QTcB and QTcF on one measurement, but this was transient and resolved within 24 hours. Further, in many cases, prolongation was clearly due at least in part to the confounding effects of fever, malaria and the increased heart rates associated with both. Overall, the drug effect was modest in this population, and similar to what has been seen in the other large phase 3 studies.

In 2012, AFRIMS conducted a randomized, double-blind clinical study WR 1849, “Malaria Prevention Cambodia”, comparing a 2 day course of DHA-piperaquine to placebo in healthy military volunteers in northern Cambodia. Intensive cardiac safety monitoring of the QTc interval was conducted, with oversight and expert review by an unblinded Data Safety Monitoring Board made up of 2 board certified cardiologists, and chaired by an experience clinical pharmacologist. Prespecified cohort safety rules included individual halts for sustained QTc interval prolongations greater than 500 ms (grade 3 adverse events), and an unblinded review of all volunteer cardiac safety data if/when 4 volunteers were halted. Unexpectedly, 4 healthy volunteers met individual halting criteria with transient QTc prolongations at peak expected piperaquine concentrations measured at 4 hours post dose. This occurred in the first or second month of dosing for all those halted. While QTc prolongations resolved in all cases and returned to baseline within 24 hours as

CONFIDENTIAL

expected based on the pharmacology of piperaquine, the study was halted based on the a priori halting rules with the following recommendations from the DSMB:

1. Discontinue enrollment in the study now and do not re-challenge previously "Halted" subjects.
2. Amend the protocol to intentionally reduce peak systemic exposure to piperaquine by either dosing over 3 days instead of 2 days AND/OR administer medication after a period of fasting.
3. Consider further targeted research to evaluate the 4 "Halted" subjects (regarding PK and electrocardiographic PD).

QTc prolongations seen in this low risk population were transient and clinically insignificant. However, it remains possible that the piperaquine may cause a clinically significant effect on the QTc interval following a single treatment course in high risk populations including as those with congenital long-QT syndrome, or acquired long-QT syndrome due to concomitant QT prolonging drug administration. Therefore, cardiac EKG monitoring will be performed in the present study, incorporating the lessons learned and essential safety monitoring features of WR 1737 and WR 1849. Because malaria can itself prolong the QTc interval due to fever and tachycardia, exclusion and follow-up criteria from WR 1737 (also a DHA-piperaquine treatment study) will be used.

This approach is in line with recent recommendations for the DHA-piperaquine product manufactured by Sigma-tau pharmaceuticals (Eurartesim). The packaging and labeling information of Eurartesim was recently made publicly available (see Appendix E). In addition to fasting where possible prior to administering the drug, the following recommendations were made:

"When clinically appropriate, consideration should be given to obtaining an ECG from all patients before the last of the three daily doses is taken and approximately 4-6 hours after the last dose, since the risk of QTc interval prolongation may be greatest during this period (see section 5.2). QTc intervals of more than 500 ms are associated with a pronounced risk for potentially life-threatening ventricular tachyarrhythmias. Therefore, ECG monitoring during the following 24-48 hours should be applied for patients found to have a prolongation to this extent. These patients should not receive another dose of Eurartesim and alternative antimalarial therapy should be instituted." (Appendix E, page 5)

The current protocol is thus designed to include these assessments and safety monitoring procedures as recommended.

3.4 Primaquine

The rapid identification and treatment of malaria patients with drugs such as DHA-piperaquine that effectively clear blood stage infection will be crucial in future malaria elimination/eradication efforts. The 8-aminoquinolone compound primaquine has

CONFIDENTIAL

limited use as a blood schizonticide but, importantly, it has unique effects on stages of the malaria parasite not demonstrated by other available licensed antimalarials. Primaquine has an effect on non-dormant liver stages (merozoites) of both *P. falciparum* and *P. vivax*, and thus is the only effective agent with causal (liver stage) activity. This activity in the liver extends to clearance of hypnozoites of *P. vivax* and *P. ovale.* Additionally, primaquine also has effects on the gametocyte stage as well. This is particularly pertinent for the success of control programs in which the elimination of circulating gametocytes in asymptomatic or recently treated persons will prevent the transmission and spread of malaria in the community. The current WHO guidelines for treatment of malaria infection include a recommendation for a single dose of primaquine at conclusion of treatment for clinical infection with *P. falciparum* (WHO Guidelines for the treatment of malaria, 2nd ed. 2010). This recommendation is pertinent at this time only for low transmission areas where gametocyte carriers are responsible for maintenance of transmission of the disease. The treatment dose recommended is 0.75 mg/kg to be given orally with a maximum dose of 45 mg.

Although this recommendation exists, transmission blocking strategies have not been widely or comprehensively pursued to date, and clinical evidence for effectiveness is limited. A significant purpose of this study is to develop good clinical evidence for this strategy, and determine its appropriateness. While a single primaquine dose is recommended, gametocytes have been observed to circulate for up to several weeks following resolution of an asexual blood stage infection. Further, the biology of asymptomatic sexual stage malaria is not as well understood as that of the asexual stage.

Appearance of *Plasmodium* sexual stages, i.e., male and female gametocytes, in the peripheral blood that are transmissible to female *Anopheles* mosquitoes is estimated to occur 7-14 days after emergence and replication of asexual stages in the bloodstream (Bousema, 2011). The triggers for development of an asexual blood stage merozoite into the sexual stages are unknown. Upon invasion of a red blood cell, a merozoite committed to differentiation into a gametocyte must progress through five stages (I-V) of maturation. The initial immature stages of gametocytes (Stages I-IV) are absent from the peripheral circulation and are thought to be sequestered in small blood vessels and perhaps in the bone marrow and spleen. Mature gametocytes (Stage V) are then released into the peripheral circulation, although it takes 2-3 days to become infectious for feeding mosquitoes. The sequestration period can be as long as 12 days, followed by an indeterminate period of circulation in the blood stream. Thus gametocytes could appear weeks after successful treatment of a clinical episode, leaving recently treated patients to serve as a reservoir for transmission of malaria in their communities.

To complicate the matter of sequestration, mature gametocytes comprise <5% of circulating parasites and thus can be difficult to detect by light microscopy; however, despite circulating at such low densities, mosquitoes are able to take up gametocytes efficiently, resulting in transmission of infection (Coleman 2004,

CONFIDENTIAL

Schneider 2007). Molecular techniques such as PCR can aid in detecting low level gametocytemia (Bousema 2006, Shekalaghe 2007) and a recent meta-analysis estimated that gametocytemia is detected on average 50% less by light microscopy compared to PCR methods (Okell 2009).

Unfortunately, antimalarial drugs used to treat a clinical infection may not also eliminate gametocytes from the bloodstream (Bousema, 2011). Drugs such as quinine, chloroquine and sulfadoxine-pyremethamine have limited effects on gametocytes and some studies have reported these drugs can increase the number of gametocytes in the peripheral blood, although this may be due to immature gametocytes being flushed from sequestration (Targett, 2001, Robert 2000, Dunyo 2006). Artemisinins rapidly clear asexual parasites in the bloodstream and are thought to affect numbers of immature gametocytes as well (Pukrittayakamee, 2004, Chotivanich 2006). Thus, this class of drugs can affect gametocytemia both directly by clearing immature gametocytes, as well as indirectly by killing circulating trophozoites and schizonts so the numbers available to later differentiate into gametocytes is effectively decreased. Primaquine, which is only minimally effective against the asexual blood stages of P. falciparum, is the only licensed antimalarial demonstrated to be effective in killing mature gametocytes.

A study done in Tanzanian children with *P. falciparum* infection illustrates the benefit of the WHO policy of a single dose of primaquine (Shekalaghe, 2007). Children aged 3-15 years were randomized to SP and AS (single dose SP with three days of artesunate) plus a onetime dose of primaquine (0.75 mg/kg) or placebo at the conclusion of treatment for malaria infection. The prevalence of volunteers with gametocytes detectable by microscopy at baseline ranged from 19-25%, but when evaluated by PCR, the prevalence was much higher at 88-91%. On day 14 post-treatment there was a large difference in prevalence of volunteers with PCR-detected gametocytemia: 4% in SP+AS+PQ group and 63% in SP+AS+placebo group. In addition, both the density of gametocytes and duration of carriage were statistically significantly lower in the group treated with PQ. Such results were replicated in a study done by Smithuis et al (2010) in Myanmar. In this study five groups of approximately 160 volunteers each all with uncomplicated *P. falciparum* malaria received various ACT treatments. In each group, half the volunteers received a onetime dose of primaquine of 0.75mg/kg. All volunteers receiving primaquine has approximately a 12-fold reduction in gametocyte carriage by light microscopy (rate ratio 11.9, 95% CI 7.4-20.5, p<0.0001). In Sudan however, there was no benefit in adding a onetime dose of primaquine to SP +AS therapy in asymptomatic adults with submicroscopic *P. falciparum* parasitemia (El-Sayed, 2007) In this study baseline gametocyte prevalence by RT-PCR was only 12%. The evidence, taken as a whole, suggests that artemisinin treatment lowers gametocytemia, with a onetime primaquine dose appearing to further this effect in most studies. However, this evidence does not confirm a reduction in transmissibility since the transmissibility of gametocytes cannot be determined by light microscopy alone. None of these studies included confirmatory evidence of patient to vector transmission.

CONFIDENTIAL

Besides the beneficial effects of primaquine on gametocyte carriage, another study done in South Africa examined the relationship between drug resistance and gametocyte carriage (Barnes, 2008). Over a 5 year period, in an area of low transmission where SP was used as first line therapy, as the amount of resistance to SP increased (as manifested by genetic mutations in dhfr and dhsp genes), so did the density and duration of peripheral gametocytemia. The geometric mean density for parasites with genetic mutations conferring resistance to SP was 1212 gametocytes/mcL/week while for parasites without these mutations, the geomean was 60.8 gametocytes/mcL/week (p=0.014). The duration of gametocytemia in the two groups was 45.4 weeks versus 7 weeks respectively (p=0.016). Despite the increasing number of genetic mutations conferring resistance to SP over time, the drug remained effective in treating acute infections, suggesting the mechanism of increased gametocytemia was not due to increased numbers of asexual parasites circulating due to primary drug failure. Although SP is not used for treatment of malaria in Cambodia, the lessons learned from this study regarding the relationship between drug resistance and increased density and duration of gametocyte carriage are important to note. This study will monitor for the development of resistance to DP over a three-year period; if evidence for resistance is seen, there may be a resulting effect on gametocytemia (increase in density or polyclonality for example), underscoring the need for an effective transmission blocking medication such as primaquine to reduce gametocytemia to be incorporated as part of the overall control strategy.

3.4.1 Primaquine in G6PD deficiency

Glucose-6-phosphate dehydrogenase is an enzyme which is crucial in controlling cellular oxidative stress, and patients who are deficient in this enzyme can undergo hemolysis when given primaquine. There are gradations in the degree of G6PD deficiency, thus volunteers have different tolerances for primaquine. Testing at enrollment in this study will identify any volunteers who are G6PD deficient. Under an AFRIMS malaria drug resistance surveillance protocol (WR 1576), nearly 10.5% of all malaria patients were G6PD deficient by fluorescence testing. However, it is unclear whether the rates in malaria patients reflect that in the general population. Because of the potential for hemolytic anemia induced by primaquine in G6PD-deficient patients, current national guidelines in Cambodia provide for the use of primaquine only where laboratory screening tests for G6PD are available (see Appendix A). However, in most locations in Cambodia, G6PD testing is not available, resulting in the de facto absence of primaquine use.

There have been limited studies of G6PD deficiency in Cambodia, including unpublished data collected over the past 3 years by AFRIMS which revealed a prevalence of approximately 10-15% in the populations to be studied. This number has remained relatively constant. Previous reports reveal the common genotypic variants encountered in Cambodia to be Viangchan, Mahidol, Union and Coimbra. These are mild to moderate variants (WHO Class II and III) although phenotypic

CONFIDENTIAL

variation among genotypic variants can be significant and has not been well studied. In one previous study in Cambodian males, 15 mg of primaquine for 14 days resulted in a mean 21% drop in hematocrits (Everett *et al*., 1977). A single dose of 45 mg is unlikely to lead to clinically significant hemolysis, even in G6PD deficient individuals, although evidence is limited.

Safe doses of primaquine in G6PD deficient volunteers have been determined and are generally accepted. Primaquine for radical cure of the hypnozoite stages of *P. vivax* or *P. ovale* requires 14 days of therapy. A recent review by Myint et al. (unpublished) from 1948-2009 found 33 clinical studies using primaquine which included more than 500 G6PD-deficient patients. There were roughly 300 patients who presented primaquine-induced hemolysis in 20 studies, with a reduction in hematocrit ranging from 4% - 23%, all of whom were G6PD deficient. This included 40 patients in 7 studies who were reported to have their primaquine stopped because of clinical concerns, and only 25 cases from 5 studies who required blood transfusion. Changing from a daily dose for 14 days to a weekly dose of 45 mg of primaquine for eight weeks did not cause significant hemolysis in G6PD deficient African-Americans (Alving et al., 1960). This 8 week treatment dose of 45 mg per week is still considered safe in G6PD deficient patients, and recommended by US CDC.

A study in Myanmar (n=22), found that primaquine 45 mg single dose to treat *P. falciparum* gametocytes and 45 mg weekly x 8 weeks for radical cure of *P.vivax* malaria was safe and effective in G6PD-deficient volunteers (Kyaw et al., 1994). Additional studies on the use of primaquine in malaria-infected patients with G6PD deficiency in Thailand and Japan showed that the drug was safe and effective without evidence of hemolytic anemia (Charoenlarp et al., 1972). Despite this, other studies have reported that serious hemolytic reactions can occur with small doses and also with even single doses of primaquine 45 mg (Ziai et al., 1967; Reeve et al., 1992). In WR 1737, there were 72 *P. vivax* patients from the study who were treated with PQ at discharge, including 13 G6PD deficiency cases (18%) who received 45mg single dose/week for 8 weeks. Only 2 of 13 had a hematocrit drop > 10% (less than 15%), and both were transient and resolved within 1-2 weeks. Overall PQ was well tolerated, and there were few reported side effects associated with PQ, most commonly muscle pain and abdominal discomfort.

In this study, all volunteers will be tested for G6PD deficiency although randomization to single dose of primaquine will not be based on G6PD status. All volunteers will have a CBC done the day before and the day after primaquine treatment to assess for any hemolysis. The hematocrit will be rechecked on days 7 and 14 for those volunteers who are found to be G6PD deficient to ensure that no significant hemolysis occurs. Although it would be easy to advocate for exclusion of G6PD-deficient volunteers, the principle of ‘justice’ suggest that G6PD deficient volunteers should be included - they are at equal risk for malaria and would benefit from the research.

CONFIDENTIAL

3.5 Mosquito infection by membrane feeding

Assessment of the efficacy of DP and DP/PQ on peripheral gametocytemia and potential transmissibility of infection will be done by multiple methods. First, the prevalence of gametocytemia at baseline and before and after treatment will be determined by light microscopy as well as PCR. As outlined above, both DP and PQ are thought to have gametocidal action although affecting different stages of the gametocyte life cycle; therefore the use of DP alone as well as DP/PQ will be important to evaluate the potential effect of a onetime dose of PQ on transmission of infection.

In addition to the prevalence of gametocytemia, it will be beneficial to assess whether DP or DP/PQ affects the actual transmission of gametocytes to female *Anopheles* mosquitoes, indicating risk reduction for ongoing malaria transmission in the community. This is particularly pertinent since gametocyte densities are often submicroscopic yet capable to transmitting infection. The Department of Entomology at AFRIMS has developed an *Anopheles* mosquito membrane feeding assay used in several previous studies. This assay is mainly used to produce infectious sporozoites to be used for various laboratory assays at AFRIMS, although most recently this assay has been successfully used to conduct *P. vivax* human malaria challenges at WRAIR using mosquitoes transported from Thailand to the United States.

In human challenge studies conducted under WRAIR #1308 (Principal Investigator: Dr. Ratawan Ubalee), *P. vivax* infected volunteers are recruited and enrolled at the Thai Ministry of Public Health (MOPH) Malaria Clinics in Mae Sod, Thailand. Samples of *P. vivax* infected blood are collected and fed via membrane feeding apparatus to colony-reared *Anopheles dirus* mosquitoes from the AFRIMS Entomology Lab. Membrane feeding has been determined to be highly efficient with over 80% mosquito infection rates typically observed (Prachumshri, unpublished). After screening donor blood for potential co-infections (including HIV, HBV, HCV, Syphilis, JE virus, CK virus, and mircofilariae), infected mosquitoes are transported by commercial airliner to the US with the remainder of sporozoite development occurring at the WRAIR Division of Entomology insectary. Approximately 16-24 days after feeds, salivary gland sporozoite development is complete, and the mosquitoes are used to feed on human volunteers. These studies are FDA-regulated and conducted under IND and have been indispensable in evaluation of *P. vivax* vaccine candidates.

This same concept of infecting mosquitoes with a membrane feeding assay will be used in this study; although with a goal of determining if an antimalarial regimen (DP or DP/PQ) is effective in killing all stages of gametocytes and preventing malaria transmission to *Anopheles* mosquitoes, and thus the population at large. Mosquitoes will feed via membrane feeding apparatus on blood drawn from volunteers, and then the fed mosquitoes selected and incubated in appropriate environmental conditions according to Entomology SOPs at the insectary located at Anlong Veng MTF. At

CONFIDENTIAL

nine days post-feed, half of the mosquitoes will be dissected by a trained technician and any midgut oocysts, which indicates transmissible gametocytes were indeed present in the blood, enumerated. For the remaining half of the mosquitoes, half will be dissected and oocysts/midguts preserved for future molecular analysis and half will remain in the incubators for another approximately seven days (16 days post feeding), or the length of time it takes to develop salivary gland sporozoites. At this stage, these mosquitoes will then be preserved for molecular analysis.

While the membrane feeding assays is well developed and a reliable assay for producing *Plasmodium*-infected mosquitoes with infectious salivary gland sporozoites, the use of this membrane feeding assay to look for midgut oocysts and sporozoite development in patients with clinical malaria infection is exploratory. An important secondary objective of this protocol is to attempt to standardize this assay for efficient application in the field (i.e., it would not be used a supportive data in a 501K application to the FDA). A well-validated membrane feeding assay will be an important tool for developing and ensuring the feasibility of malaria elimination. AFRIMS in partnership with CNM is well positioned to develop a definitive and easily applicable assay in the field.

This effort represents an opportunity for technology transfer and capacity building in the development and implementation of the membrane feeding assay. Identified personnel from the community, RCAF and/or CNM will train at AFRIMS prior to commencement of this study in maintenance of colony-reared *Anopheles* mosquitoes, midgut dissection, oocyst enumeration and preservation, and sporozoites for molecular analysis and membrane feeding techniques. Female *Anopheles* mosquitoes will be raised at AFRIMS labs in Bangkok will be transported to study sites weekly for use. AFRIMS will support CNM and local entomologists to maintain the appropriate standard operating procedures necessary to conduct the assay in a reproducible fashion. Mosquitoes will be stored in secure containment facilities during transport and while on site.

4 STUDY OBJECTIVES

4.1 Primary Objectives

1. To monitor therapeutic efficacy (based on rates of recurrence at 42 days) and search for evidence of drug resistance of a fixed-dose 3 day regimen of DHA-piperaquine (DP), with and without a dose of primaquine, in volunteers with uncomplicated *P. falciparum* infection in Cambodia over a 3-year observation period.

2. To establish the transmission blocking (sexual stage) efficacy of the prescribed drug regimen with or without a single oral 45 mg dose of primaquine.

CONFIDENTIAL

4.2 Secondary Objectives:

1. To document the safety and tolerability of DHA-piperaquine, including the effect on the electrocardiogram (EKG), particularly the QTc interval, in patients taking 3 day treatment courses of DHA-piperaquine.

2. Assess the degree of antimalarial drug resistance in the parasite populations in Cambodia by correlating 42 day rates of malaria recurrence clinical and pharmacodynamic outcomes (parasite clearance) with pharmacokinetic drug levels, *in vitro* drug susceptibility testing, genomic studies and molecular markers of drug resistance.

3. To quantify the reduction in sexual stage parasites (gametocytes) of the two treatment regimens assessed by 3 methods on volunteers'' samples – light microscopy, PCR and a mosquito membrane-feeding assay.

4. Assess the relative contributions of clinical history, baseline immunity levels and parasitologic parameters associated with prior infection on clinical outcome and parasitological responses *in vivo*.

5. To build host nation capacity, with emphasis on training laboratory, clinical and entomological scientists to conduct antimalarial therapeutic efficacy and drug resistance studies.

6. To cryopreserve parasite isolates to standardize antimalarial resistance surveillance monitoring methods *in vitro*.

7. To provide up-to-date antimalarial efficacy data to the Cambodian government, including CNM and Ministry of National Defense, to help determine the appropriate regimens of DHA-piperaquine and primaquine for the treatment of uncomplicated malaria.

8. To harmonize clinical and laboratory approaches to characterizing antimalarial drug resistance between DoD-GEIS overseas labs including, AFRIMS, USAMRU-K, and others.

9. To characterize falciparum population genetic structure and study the transmissibility of genetic variants to mosquitoes by membrane feeding.

CONFIDENTIAL

5 STUDY DESIGN

5.1 Overview

This is an active open label **Treatment Study** evaluating the efficacy, safety, tolerability and pharmacokinetics of a standard three day course of Dihydroartemisinin-Piperaquine (DP) in uncomplicated P. falciparum malaria. This study will continue over an estimated 3-year period to observe and document any changes in resistance patterns to this first-line ACT treatment regimen. Cardiac safety monitoring will be conducted with focus on peak QTc values on day 3 during convalescence to avoid the confounding effects of fever and tachycardia.

On day 3 of DP therapy, volunteers will be randomized to receive a one-time dose of 45 mg of primaquine or no treatment. For volunteers receiving PQ, the onetime dose will be administered on the same day, at least 2 hours after ingestion of DP. Investigators will be able to detect any difference in therapeutic efficacy of DP versus DP-PQ as well any effects of primaquine on persistence of gametocytemia and, using membrane feeding assays, on the transmissibility of *P. falciparum* infection.

Follow-up for all volunteers will occur over approximately 42 days with weekly peripheral blood smears with PCR correction to detect any malaria infection occurring during this time period. Malaria smears will be performed if malaria symptoms are experienced outside the scheduled visits. If the volunteer develops a malaria recurrence, blood will be drawn for resistance markers and piperaquine levels, and the volunteer treated with an alternative regimen based on national policy guidelines for either *P. falciparum* or *P. vivax*.

5.2 Endpoints

The main objectives of this study are as stated in Section 4: to measure the 42-day clinical efficacy of the antimalarial drug regimen of DHA-piperaquine, with and without a 45 mg dose of primaquine, over a 3 year period in selected areas of Cambodia where malaria transmission is actively occurring. Secondly, the study will aim to detect any beneficial effects of a onetime dose of primaquine after completion of therapy for blood stage infection on gametocytemia that may persist after DP treatment. To achieve these objectives, the following endpoints will be executed.

CONFIDENTIAL

Primary Endpoints:

1. Efficacy rates at 42 days (with 95% confidence intervals) for DP with and without single dose primaquine for uncomplicated *P. falciparum* diagnosed by positive PCR-corrected malaria microscopy.

2. Comparative rates of sexual stage infections at days 1, 4, 7 and 14 between patients dosed with and without primaquine based on a combined endpoint of light microscopy, PCR analysis for detection of gamteocytes and mosquito membrane feeding assay.

Secondary Endpoints

1. Efficacy rate at 28 days (with 95% confidence intervals) for DHA-piperaquine for uncomplicated *P. falciparum* diagnosed by positive PCR-corrected malaria microscopy.

2. 28- and 42-day comparative asexual and sexual efficacy rates of DHA-piperaquine with and without single dose primaquine for uncomplicated *P. falciparum*

3. Kaplan-Meier survival analysis of asexual and sexual blood stage efficacy at days 7 and 14, and analysis of asexual stage only at days 21, 28, 35 and 42.

4. Comparative reduction in mosquito oocyst prevalence at days 4, 7 and 14 post-treatment for DP and DP-PQ.

5. Comparative rates, duration and intensity of treatment-related adverse drug events, and total adverse events in each treatment group, including rates of QTcF interval prolongation on EKG during the convalescent phase of disease.

6. Pharmacokinetic drug levels of piperaquine at select time points (including day of failure if a recrudescence), and primaquine on the day following treatment.

7. Drug resistance against locally available antimalarial drugs based on patterns of *in vitro* parasite growth inhibition (IC_{50}).

8. Estimate of apparent rates of preexisting immunity to malaria based on medical history, days of fever prior to presentation, antibody levels, and presenting parasitological parameters (eg. gametocytemia, low asexual stage parasitemias) and the relative contribution of these parameters to clinical and parasitological outcomes.

CONFIDENTIAL

9. Incidence of qualitative and quantitative G6PD deficiency in the study population

10. Rates of relapse with *P. vivax* malaria during the study.

11. Using genomic tools, evaluate the complexity of infection and genetic diversity of malaria parasites in the major life cycle stages- asexual, gametocytes, oocysts and sporozoites.

5.3 Sample Size

The primary end-point for sample size purposes will be 42 day efficacy of DP for uncomplicated *P. falciparum*. Estimates of treatment cure rates and 95% CIs (exact) will be reported on at least an annual basis until the surveillance activity ceases or changes substantially (eg, a new first-line ACT is introduced, or there is no longer an interest by Cambodian authorities in monitoring DHA-piperaquine efficacy). The intended sample size will be 150 evaluable subjects. Using a point estimate for 42 day efficacy of 94%, the 95% confidence interval for the estimate of true efficacy will be approximately 89-97% (n = 150).

Volunteers developing malaria will randomized to either 45mg single dose primaquine or no primaquine treatment on day 3 of DP treatment and the effects of PQ on the sexual stage gametocytes will be explored. There are no statistical assumptions or power calculations for this analysis as it remains exploratory – little data is available on which to develop assumptions. The purpose of this effort is to gather preliminary data on gametocyte carriage rates, and gather quantitative evidence on the effects of single dose primaquine on the sexual parasite stage.

5.4 Duration of Volunteer Participation

Volunteers who enroll in the study will be treated and followed for a minimum of 42 days with discharge from the study at that time if demonstrated cure of malaria infection. In prior published studies using DP in Southeast Asia, efficacy is estimated to be 98% for a 3-day regimen (Krudsood, 2007).The exact length of follow-up of the cohort will be determined by the number of volunteers developing recurrent malaria during the 42- day follow-up period. Volunteers that have a malaria recurrence during the 42 day treatment follow-up period will be re-treated for blood stage malaria under national guidelines and continue follow-up for the remainder of the 42-day period. If blood stage malaria recurs in week 5 or 6 of the study, volunteers may have follow-up extended until they have clinical resolution of symptoms and two negative blood smears at least one week apart.

CONFIDENTIAL

5.5 Study Group Descriptions

Patients assessed as having uncomplicated malaria will be enrolled in open label fashion to a 3-day treatment course of DHA-piperaquine (DP) by directly observed therapy (DOT) at the MTF. All patients will receive a total of 9 tablets containing 40mg DHA and 320mg of piperaquine in divided doses at 0, 24 and 48 hours (3 tablets once per day) for the 3 day course. Medication compliance for malaria treatment will be assured by directly observed therapy by study personnel during dosing. At completion of DP treatment volunteers will be randomized in an open label fashion to receive a single 45 mg dose of primaquine or no therapy, and this will also be administered under directly observed therapy at the MTF. All volunteers will then be followed approximately 42 days to evaluate study objectives and endpoints.

5.6 Population to be Studied

The study population will include adult (age 18 - 65 years) civilian and military volunteers living in areas determined to have high incidence rates of malaria based on current estimates by AFRIMS, CNM and the RCAF health services. Active duty military personnel will be required to obtain permission from their Commanders prior to enrolling. Pregnant volunteers will be excluded from participation in the study, due to risk of teratogenicity from artemisinin derivatives. Children and adolescents will not be enrolled in this study.

5.7 Study Sites and Selection of the Study Population

5.7.1 Population Characteristics

General characteristics of the intended study populations in Cambodia are listed below. See Background Section 3.2 for full details.

- Ethnic composition: Khmer 98-99% and Vietnamese 1-2 %
- Typical living condition: relatively poor, majority farmers (corn, bean or peanut plantations) and loggers.
- There are two different population groups living in the area:
    - Long term residents, living in the area for more than 5 years, most of whom own the land they are working on.
    - New residents who remain a minority but make up a fast-growing segment of the population in many border areas. The majority have moved to border areas in the past few years, coming from other Eastern provinces in Cambodia, (such as Kampong Cham, Takeo, and Kandal Province). New residents mostly make a living in forestry, hunting or as laborers, and in these trades are thought to be the group most affected by malaria. They

CONFIDENTIAL

also have more limited access the health care system and develop severe malaria more frequently than long term residents.

- The average annual income for an individual in Cambodia is approximately 2,100 USD (CIA, 2010).
- Level of education: Mostly primary and secondary school only, but according to official statistics most of the population over 18 years is literate (95%).

5.8 Description of Test Article

The test article during the Treatment Study will be the commercially available product Duo-Cotecxin, manufactured by Zhejiang Holley Nanhu Pharmaceutical Co., Ltd (see Appendix B for Package insert). This is the current first-line ACT recommended for in WHO containment Zone 1 in Cambodia (along the western border with Thailand). The test article will be procured through the CNM, who will use the current government-approved manufacturer. The IRBs will be provided with a package insert, should CNM switch procurement policy to import DHA-piperaquine from an alternative manufacturer. This is a combination tablet containing 40 mg of dihydroartemisinin and 320mg of piperaquine phosphate in each tablet. Certificates of analysis from the manufacturer will be provided to the IRBs, along with independent analysis reports from AFRIMS Pharmacology lab for DHA-piperaquine prior to study start.

Primaquine phosphate will be obtained from The Government Pharmaceutical Organization (GPO), Bangkok, Thailand. Medication will be supplied in 15 mg tablets to ensure the correct dose is administered (see Appendix C for Package insert).

5.8.1 Packaging and Labeling of the Test Articles

The test articles will be used in the original commercial packaging, but administered by study personnel as described in section 6.7.

5.8.2 Storage of Test Articles

The test articles will be stored in a cool, dry place below 30° C in a light-proof container. The test articles will remain under secure custody of the study team at all times.

CONFIDENTIAL

5.9 Monitoring of Clinical Subject Safety

See Section 6.9 for full details regarding clinical assessments of Volunteer safety. Briefly, volunteers will be monitored during all phases of the study for adverse events. The most important component of monitoring will include the active malaria case detection and treatment which is the focus of the study. Treatment related adverse events are relatively rare at therapeutic doses with DP but include disturbances in cardiac potassium channel conductance which can prolong the QT interval on the EKG although to date there is not good evidence of clinically significant QT prolongation at therapeutic doses to be used in this study (see Section 3.3). EKGs will be monitored for QTc interval prolongation with focus on the period of peak drug concentration (day 3 post-dose), while the patient is in the convalescent phase of illness to avoid confounding by fever and tachycardia. Oversight and expert review of EKGs will be provided by a Cardiac Data Safety Monitoring Board (see Sections 6.15 and Appendix F). Neurological toxicity is rare at therapeutic dihydroartemisinin doses but has been reported and will be monitored as part of routine clinical assessments which include a directed physical exam to further investigate neurological complaints as appropriate.

Primaquine in G6PD deficient patients can potentially cause hemolysis although most often seen in prolonged 14-day therapy for radical cure. A one-time dose of 45 mg will be given in this study with a CBC performed in all volunteers on the day prior and day post-therapy, as well as on Day 7 and 14 for G6PD deficient volunteers as outlined in Table 1. Although there is little or no risk in G6PD-normal patients, primaquine has the potential to induce hemolytic anemia in G6PD-deficient patients. G6PD-deficient subjects with anemia at enrollment will be carefully evaluated by the investigator and excluded if there is evidence of clinically significant anemia. A CBC will be obtained at enrollment with repeat CBC on day 3 following the primaquine dose. Additional CBC monitoring will be performed if the hematocrit drops more than 10% on the day following the primaquine dose compared to the previous day, with CBCs repeated on days 7 and 14 after enrollment.

Subjects with signs of severe malaria at presentation may require treatment for severe malaria with parenteral therapy according to the Cambodian national guidelines (Appendix A). Severe malaria by WHO criteria is defined as coma or seizures, pulmonary edema, shock, renal failure, jaundice, severe anemia, spontaneous bleeding, hyperparasitemia (>5% RBCs infected), or prostration. While uncomplicated malaria often presents with mild hepatic and/or renal insufficiency, the criteria defining severe malaria with regard to renal and hepatic insufficiency are based on clinical evidence of organ dysfunction (oliguria and/or jaundice). Therefore, subjects with mild subclinical renal and/or hepatic insufficiency as evidenced by clinical lab value abnormalities alone do not require parenteral therapy under the national treatment guidelines. To date there is no evidence in the literature that the pharmacokinetics of either drug is altered substantially in subclinical hepatic or renal insufficiency, and this study may in

CONFIDENTIAL

fact add evidence in this regard with careful measurement of both pharmacokinetics and renal and hepatic laboratory monitoring as outlined in the protocol.

CONFIDENTIAL

6 METHODS

6.1 Recruitment of Study Volunteers

Potential volunteers will be identified by the study team and/or local medical providers when they present with uncomplicated *P. falciparum* or *P. falciparum/vivax* malaria. Volunteers who present with danger signs indicating severe or complicated malaria infection will not be enrolled, but will be treated under current national guidelines for treatment of severe malaria. If the potential volunteer agrees to consider enrolling, the local staff will contact the study team immediately for enrollment. The study team will be based at two medical treatment facilities (MTF) in Battambang Referral Hospital, Battambang Province, and Along Veng Referral Hospital, Anlong Veng District.

A one-page information sheet in Khmer will be provided to local health care providers in the areas. This information will be verbally presented to potential volunteers and any questions answered. If the volunteer wishes to participate in the study, local health care providers will contact study staff who will then initiate informed consent procedures. Volunteers who have already enrolled in this study, received DP and successfully completed a 42-day follow-up period of the study or have received an alternative curative treatment course of antimalarials by the study investigators, can elect to screen for the study again should he/she develop uncomplicated malaria infection.

It is estimated that there will be up to 300 volunteers screened to obtain 150 evaluable subjects.

6.2 Informed Consent Process

Informed consent is a process that is initiated prior to the individual's agreeing to participate in the study and continuing throughout the individual's study participation. Extensive discussion of risks and possible benefits of participation in this study will be provided to the volunteers and their families. Following the study briefing, volunteers will be given the opportunity to discuss questions of a personal nature privately with the investigators and/or the ombudsman if desired following the briefing. An ombudsman is an independent individual with knowledge of the study not involved in study procedures who will meet with prospective active duty military volunteers to answer questions, and counsel them that they are not required to participate in the study, and may leave at any time without penalty or fear of reprisal (see also Section 10.10).

Consent forms in the local language (Khmer) describing in detail the study procedures and risks will be given to the volunteer and written documentation of

CONFIDENTIAL

informed consent will be obtained prior to enrollment in the study. Consent forms will be IRB approved and the volunteer will be asked to read and review the document. If the volunteer cannot read the content of the consent form, it will be read and explained to him/her in Khmer by the study investigator obtaining the informed consent with the presence of a witness or ombudsman; thus any potential volunteer must be able to speak and understand Khmer. Upon reviewing the document, the study personnel will explain the research study to the volunteer and answer any questions that may arise. The volunteer will be asked to sign and date the informed consent document prior to being enrolled in the study.

Special consideration will be given to the recruitment process for military personnel. The Chain of Command will not be involved in the recruitment of military personnel and will not encourage or order soldiers to participate in a research study. Per DOD Directive 3216.2, an ombudsman will be employed when conducting group briefings with active duty personnel to ensure that volunteers have been told that participation is voluntary. The ombudsman will be present in other situations as appropriate, and will be available to volunteers to answer questions. Volunteers will have the opportunity to discuss the study with the assigned unit ombudsmen if military personnel or think about it prior to agreeing to participate.

One witness will sign and date the consent form in the presence of the participant attesting that the requirements for informed consent have been satisfied and that consent is voluntary and freely given by the volunteer without any element of force, fraud, deceit, duress, coercion, or undue influence. Participation in the study will be voluntary and volunteers will be informed that they may withdraw consent at any time throughout the course of the study. Following ICH guidelines a signed copy of the informed consent document will be given to the volunteers for their records. The rights and welfare of the volunteers will be protected by emphasizing to them that the quality of their medical care will not be adversely affected if they decline to participate in this study.

CONFIDENTIAL

6.3 Determination of Eligibility

Volunteers not meeting all study inclusion and/or exclusion criteria will not be enrolled into the study. A screening log will be kept of all who were evaluated for participation to document who was and was not enrolled and reason for not enrolling in the study.

6.3.1 Inclusion Criteria

Volunteers meeting all of the following criteria will be considered eligible for enrollment in the study:

1. Volunteer with uncomplicated *P. falciparum* malaria (volunteers with mixed *P. falciparum* and *P. vivax* infections may be enrolled), 18-65 years of age

2. Baseline asexual parasite density between 1,000-200,000 parasites/μL

3. Able to provide informed consent

4. Available and agree to follow-up for anticipated study duration including 3 day treatment course at the MTF and weekly follow-up for the 42-day period

5. Authorized by local commander to participate if active duty military

6.3.2 Exclusion Criteria

Volunteers meeting any of the following criteria will be excluded from the study**:**

1. Allergic reaction or contraindication to DHA, piperaquine or primaquine

2. Significant acute comorbidity requiring urgent medical intervention

3. Signs/symptoms and parasitological confirmation of severe malaria

4. Use of any anti-malarial within the past 14 days.

5. Class I or II G6PD deficiency (defined as severe) as determined at screening

6. Pregnant or lactating female, or female of childbearing age, up to 50 years of age, who does not agree to use an acceptable form of contraception during the study

7. Clinically significant abnormal EKG, including a QTcF interval > 500 ms at enrollment.

8. Known or suspected concomitant use of QTc prolonging medications.

9. Judged by the investigator to be otherwise unsuitable for study participation

WHO guidelines state that in uncomplicated malaria in pregnant women, artemisinin combination treatment should only be used starting in the second trimester, with use in the first trimester only if no other effective treatment is available. Therefore, avoidance of conception during the potential period of treatment in the study is warranted. The guidelines on contraceptive use are

CONFIDENTIAL

based on FDA guidance M3 “Nonclinical Safety Studies for the Conduct of Human Clinical Trials for Pharmaceuticals” which describes safety considerations for the inclusion of women of childbearing age in studies of healthy volunteers. This guidance is nearly identical to that of ICH M3. The guidance requires that a highly effective method of birth control be used by women of childbearing age in healthy volunteer studies. According to the published guidance, “A highly effective method of birth control is defined as one that results in a low failure rate (i.e., less than 1 percent per year) when used consistently and correctly, such as implants, injectables, combined oral contraceptives, some intrauterine contraceptive devices (IUDs), sexual abstinence, or a vasectomized partner. For volunteers using a hormonal contraceptive method, information regarding the product under evaluation and its potential effect on the contraceptive should be addressed.” The guidelines are noteworthy for omitting mention of women who have undergone surgical sterilization (these individuals would be included in the study) in addition to vasectomized partners. Clinically significant drug-drug interaction with hormonal contraceptives appears unlikely. Piperaquine undergoes very little metabolic transformation in humans and as a result is unlikely to affect the level of hormonal contraceptives (Liu et al, 2007). Artesunate and dihydroartemisinin are not extensively metabolized in liver, and there is no significant effect on the cytochrome P450 enzyme system (in vitro data) (Bangchang et al, 1992; Barradell & Fitton, 1995a).

At study entry, females will be counseled to agree to avoid becoming pregnant during their entire participation in the study, and for at least one month after the last dose of study medication. Female volunteers who suspect that they may be pregnant will be instructed to inform study personnel as soon as possible.

All females between the age of 18 and 50 will be screened with a urine pregnancy test at baseline. Pregnant or lactating females, and females of childbearing age who do not agree to use a highly effective method of birth control will be excluded from participation in the study. Females found to be pregnant at screening will be treated according to current Cambodian national malaria treatment guidelines for the treatment of malaria in pregnancy published by the Ministry of Health.

In the highly unlikely event that a female volunteer becomes pregnant during the 3 days of malaria treatment following an initial negative urine pregnancy test at the initiation of treatment, she will be discontinued from study medication and will be treated according to current Cambodian national malaria treatment guidelines for the treatment of malaria in pregnancy published by the Ministry of Health. If a woman is found to be pregnant less than 1 month after completing a course of study medication, she will be followed for safety at 3, 6 and 9 months at which time the health and birth weight of the child will be assessed. All such pregnancies temporally associated with study drug administration will be reported to the IRBs urgently.

CONFIDENTIAL

6.4 Screening Procedures

Following documentation of informed consent and determination of eligibility, study volunteers will have

- Initial targeted medical history and physical examination performed by a study physician, including a directed prior clinical history of malaria, and an electrocardiogram (EKG).
- Volunteers will have blood drawn for
    - malaria smear/PCR correction (including malaria parasite densities, both asexual and sexual stages)
    - Malaria antibody titer(s) to one or more antigens,
    - CBC
        - Remaining blood from CBC sample will be frozen at -20 ° C for hemoglobin typing assay
    - G6PD activity and G6PD genotyping
    - Baseline renal function and liver function testing
    - Parasite drug resistance characterization *in vitro*
    - Analysis of molecular markers of infection to include PCR genotyping
    - Baseline pharmacokinetic drug level (including *ex vivo* P. falciparum bioassay)
    - *ex vivo* mosquito membrane feeding assay
    - Gametocyte PCR
    - Electrolytes to include serum calcium, potassium and magnesium
- Volunteers with significant electrolyte deficiencies will be given oral supplementation.
- Counseling will be provided to volunteers who are found to be G6PD deficient, and the study team will explain ramifications for future drug treatment. A G6PD deficiency alert card will also be provided for subject safety which may be presented to the subject's primary care givers.

6.5 Randomization and Volunteer Assignment

Volunteers who enroll will be administered the first dose of DHA-piperaquine (DP) by DOT at the medical treatment facility if they meet all eligibility criteria. Volunteers with uncomplicated *P. falciparum* malaria who meet inclusion and exclusion criteria and complete a 3-day course of DHA-piperaquine will be randomized to either 45mg single dose primaquine or no primaquine treatment on day 3 of DP therapy. Treatment will be directly observed in volunteer observation at the MTF.

CONFIDENTIAL

6.6 Blinding

Although this is an open label (unblinded) study, microscopists are blinded to each others' readings and to study drug regimen. There is otherwise no blinding during the study.

6.7 Administration of Test Article

Volunteers will receive a total of 9 tablets of DP for the 3 day course. Each tablet contains 40mg DHA and 320mg of piperaquine. Volunteers will receive 3 days of treatment: 3 tablets on day 1 (at the time of diagnosis), and at 24 and 48 hours later (+/- 1 hour). Medication compliance for all malaria treatment will be assured by directly observed therapy by study personnel during dosing. Only those study personnel designated by the Principal Investigators will be authorized to administer the test article.

Study drug will be administered following at least 3 hours of fasting where possible. At enrollment, volunteers will be queried regarding last meal consumption and the time noted. However, the first dose of study drug administration will not be delayed unnecessarily if the volunteer has consumed food within the past 3 hours. On subsequent doses, study drug will be administered following at least a 3 hour fast.

On Day 3 of DP administration, primaquine (45 mg) will be given to the volunteers who were randomized to receive primaquine treatment. Primaquine will be given by DOT, three tablets of 15 mg each, as described above following the DHA-piperaquine post-dose EKG (scheduled at approximately 52 hours).

Volunteers already treated for malaria initially under the protocol who subsequently develop primary blood stage *P. vivax* or who have an apparent relapse from latent liver-stage disease will be treated according to current Cambodian national malaria treatment guidelines, which includes a three day course of an ACT to clear blood stage infection. Because DHA-piperaquine is the study drug, an alternative 1st or 2nd line agent will be used for blood stage P. vivax treatment. Currently given the high prevalence of *P. vivax* in Cambodia and chance for re-infection, radical cure with 14 days of primaquine is recommended only in settings able to screen for G6PD deficiency and provide primaquine and appropriate follow-up. Subjects may be treated by the study team or referred as appropriate to prevent relapse. Any other blood stage antimalarial medications used during the study, to include Rescue Therapy for recrudescent *P. falciparum* malaria will be supplied by the CNM and administered by the Study Team (which includes CNM Physicians) according to current national guidelines for antimalarial treatment (Appendix A).

CONFIDENTIAL

The test articles will be obtained from a commercial supplier through the CNM and the GPO, Thailand. Any unused medications remaining at the end of the study will be provided to the Battambang Referral Hospital, Battambang Province, and Along Veng Referral Hospital, Anlong Veng District for clinical use. All other antimalarial medications used during the study will be supplied through the CNM and administered by the Study Team according to current national guidelines for antimalarial treatment.

6.8 Concomitant Medications

Use of concomitant medications will be evaluated by the investigator at each clinical encounter with the volunteer. Use of antimalarials or drugs with known antimalarial activity other than those prescribed by an investigator during the study will not be permitted. While drugs that interact with or otherwise have a known unfavorable impact on the outcomes of interest in the study will be avoided by investigators during the malaria treatment phase, there are no other explicitly restricted concomitant medications during this study.

6.9 Clinical Assessments

During treatment, volunteers will have

- Vital signs including temperature, blood pressure, pulse and respirations evaluated at 4 and 8 hours after the first dose of medication, then every 8 hours until discharged
- An electrocardiogram (EKG study) will be performed at 4 hours following the first treatment dose, and at predose and 4 hours after the third dose. The average QTcF interval from 3 consecutive evaluable 10 second tracings will be measured. If the QTcF interval has both increased from screening, and is prolonged more than 480 ms (grade 2), additional EKGs will be performed before and 4 hours after the second dose. Sustained study drug-related QTcF prolongations greater than 500 ms (grade 3) on two separate EKG studies at least 15 minutes apart will be followed to resolution to predose values at 2-4 hour intervals as determined by the investigator. The DSMB will be notified of all prolongations greater than 500ms, and be provided with EKGs and a written report for review (see DSMB charter).
- Volunteers with significant electrolyte deficiencies at screening who are given supplementation may have serum electrolyte levels repeated as appropriate to determine whether supplementation was effective.
- Blood drawn for malaria smears and PCR correction at 4 and 8 hours after the first dose of medication, then every 8 hours until 2 consecutive negative smears are obtained. Malaria smears will be evaluated for both asexual and sexual stage (gametocyte) density.

CONFIDENTIAL

- Blood will be collected again for CBC, piperaquine drug level (including *ex vivo* P. falciparum bioassay), analysis of molecular markers of resistance (with PCR genotyping), and gametocyte PCR at 24, and 48 hours after the initiation of treatment. PK drug levels will also be collected at 4 and 52 hours (peak drug concentration).
- At 72 hours, the volunteer will have blood collected for malaria smear/PCR correction, PK (primaquine and piperaquine) drug levels, CBC, analysis of molecular markers (with PCR genotyping), gametocyte PCR and mosquito membrane feeding.
- The volunteer will be then discharged at the 72 hour visit after completing all procedures for out-patient volunteer follow-up if there is documentation of 2 consecutive negative blood smears. If infection persists, blood smears will continue to be prepared and read every 8 hours until 2 negative smears are obtained. If volunteer meets criteria for early treatment failure (see Section 8.2), the volunteer will be treated according to the Cambodian National Malaria Program Treatment Guidelines.

Volunteers will be followed weekly thereafter on days 7, 14, 21, 28, and 35 (-2 to +3 days):

Days 7,14

- All volunteers will have a brief clinical evaluation
- Blood will be drawn for
  - Malaria smear/PCR correction
  - piperaquine drug level (with *P.f.* bioassay)
  - mosquito membrane feeding
  - Gametocyte PCR
  - Dried filter paper spot(s) will be made from available blood for molecular marker analyses to be done at UNC
  - CBC for G6PD-defiencient volunteers ONLY
- EKG Study

Days 21, 28, 35 (-2 to +3 days)

- All volunteers will have a brief clinical evaluation,
- Blood will be drawn for
  - Malaria smear/PCR correction
  - Optional CBC for all volunteers who are G6PD deficient if needed
  - Piperaquine drug level (with *P.f.* bioassay)
- Optional EKG study if persistent QTcF prolongations

At the conclusion of the 42 day (-2 to +3 days) follow-up period, volunteers will have blood drawn for

- Malaria blood smear/PCR correction (with follow-up as per recrudescence if positive)
- Piperaquine drug level

CONFIDENTIAL

- Malaria antibody levels
- Urine pregnancy test for all females
- Optional EKG study if persistent QTcF prolongations

All volunteers with suspected recurrent malaria symptoms during the follow-up period will be re-evaluated by microscopy, and if positive for malaria will be

- Urine pregnancy test for all females (prior to administration of medication)
- Have blood drawn (prior to administration of medication) for
    - Malaria blood smear/PCR correction
    - *In vitro* drug susceptibility characterization
    - Molecular markers of resistance (with PCR genotyping)
    - Drug level for piperaquine
    - Malaria antibody levels, CBC, electrolytes, renal and liver function testing
    - Gametocyte PCR
- EKG
- Treated under directly observed therapy based on current national malaria treatment guidelines for Cambodia.

If negative for malaria, the volunteer will be referred for evaluation and treatment of alternative diagnoses to the appropriate healthcare service providers.

Volunteers found to have recurrent malaria after initial re-treatment with first line ACT therapy will be treated with rescue therapy following current national guidelines. At the time of writing, this includes treatment with artesunate and mefloquine (see Appendix A). Blood smears following rescue therapy will be collected daily until resolution by 2 negative smears, at least 6 hours apart. Any volunteer who requires alternative anti-malarial treatments because of treatment failure/recrudescence will still be followed for a 42-day period. Volunteer participation may be extended to allow for completion of malaria treatment with documentation of two negative blood smears, and a final follow-up visit with smear one week later.

CONFIDENTIAL

CONFIDENTIAL

Table 1. Table of times and events

Event	Malaria Diagnosis	4 hr	24 hr	48 hr	72 hr	MTF Dis[5]	Week 01	Wk 02	Wk 03	Wk 04	Wk 05	Wk 06	Malaria Recur
a. Informed Consent	x												
b. Medical History	x												
c. Physical Exam	x												x
d. Brief clinical evaluation[1]			x	x	x	x	x	x	x	x	x	x	x
e. Malaria smear with PCR correction and vital signs[2]	x	x[2]	x[2]	x[2]	x[2]	x[2]	x	x	x[10]	x[10]	x[10]	3.5 mL[7]	x
f. Piperaquine/primaquine drug level[9]	3 mL	2mL	2 mL	5 mL	3 mL		2 mL	2 mL	2mL	2mL	2mL	2 mL	2mL
g. Malaria antibody levels	5 mL											5 mL	5 mL
h. Renal, Liver Function, Electrolytes	2 mL												2mL
i. CBC	3 mL[8]		2 mL	2 mL	2 mL		2 mL[3]	2 mL[3]					2mL
j. Parasite culture *in vitro* resistance	8 mL												8mL
k. Molecular resistance markers[10] (+ PCR genotyping[11])	6 mL		6mL	6mL	6mL								6mL
l. G6PD	0.5 mL												
m. Gametocyte PCR[10]	2.5 mL		2.5 mL	2.5 mL	2.5 mL		3 mL	3 mL					2.5mL
n. Urine pregnancy test (all female volunteers)	x											x	x
o. Malaria treatment[4]	x		x	x									x[6]
p. Mosquito membrane feeding	2 mL				2mL		2 mL	2mL					
q. EKG study[12]	x	x	[12]	x, x			x	x	[12]	[12]	[12]	[12]	x
Daily phlebotomy in mL	32	2	12.5	15.5	15.5		7	7	2	2	2	10.5	27.5
Cumulative phlebotomy (approx mL)	32	34	46.5	62	77.5	77.5	84.5	93.5	95.5	97.5	99.5	109.5	(137.0)

1. Brief clinical evaluation includes an interval medical history, vital signs and a directed physical exam daily as clinically indicated,
2. Malaria smears/PCR correction and Vital signs to include temperature, blood pressure, pulse and respiratory rate will be done at 0, 4 and 8 hours, and then every 8 hours thereafter until the subject has had 2 negative blood smears at least 6 hours apart. Vital signs will be taken daily after two negative smears.
3. Only drawn for G6PD deficient volunteers
4. Patients will receive fixed dose 3 day course of DHA-piperaquine in equally divided doses at 0, 24 and 48 hours. On day 3 of therapy, after the post-dose EKG study has been obtained, volunteers will be randomized open label to receive either 45 mg of primaquine or no primaquine treatment. Medication compliance for all malaria treatment will be assured by directly observed therapy.
5. Volunteers will be discharged from the MTF once they are afebrile and have had 2 consecutive negative malaria smears at least 6 hours apart.
6. For Volunteers that have recurrent malaria following treatment with DHA-piperaquine, treatment will be according to current national treatment guidelines for Cambodia.
7. Estimated 3.5 mL for total blood drawn by fingerstick over entire study period. For volunteers with recrudescence at week 5 or 6, additional fingersticks beyond Day 42 may be done to document parasite clearance.

8. CBC sample at baseline will include hemoglobin typing performed at a commercial lab
9. Piperaquine level drawn at 0, 4, 24, 48, 52, 72 hours and Days 7,14, 21, 28, 35 and 42. Ex vivo activity of patient plasma against P. falciparum (bioassay) in culture will also be determined at these time points. Primaquine levels will be drawn at 52 and 72 hours. Note that on day 3 (48 hours) a total of 5 mL will be drawn at 48 (2mL) and 52 hours (3mL).
10. Molecular resistance markers will include a filter paper blood spot(s) prepared from the 6 mL draw at 24,48 and 72 hours. On Day 7, 14, and recurrence 500 microliters are added to gametocyte PCR blood draw for filter paper spot(s) for molecular marker analyses. Filter paper blood spots will be prepared on days 21, 28 and 35 from the fingerstick malaria smear.
11. PCR genotyping: 1 mL will be aliquoted from the 6 mL draw for PCR genotyping
12. An EKG study to assess the average QTcF on 3 consecutive evaluable 10 second tracings will be performed at screening, 4 hours post dose, 48 and 52 hours post dose, and week 1 and 2, and recurrence day. Additional EKGs may be performed to monitor QTc prolongations and adverse events beyond the scheduled time points as determined by the investigator – see Section 6.9.

CONFIDENTIAL

6.10 Specimen (or Data) Collection and Testing

6.10.1 Specimens to be Collected

The following specimens will be collected as outlined in the schedule in Tables 1

- Fingerstick capillary or venous blood will be collected for **blood smears by light microscopy** to determine parasite species and to quantify asexual and sexual parasitemia (approximately 200-250μL of blood per sample). PCR correction assay, to confirm parasite speciation, will be done on Day 1, if malaria recurs and other specified time points indicated by study investigators.

- **Hematology** to include hemoglobin, hematocrit, WBC count and differential, platelet count, and cell indices. Approximately 2 ml per blood draw will be collected in an EDTA (anticoagulant) tube. At baseline screening only, 3 mL of blood will be drawn and the blood remaining from CBC will be saved to perform **hemoglobin typing.**

- **Renal Function** (creatinine, urea), **Liver Function Tests** (aspartate aminotransferase, alanine aminotransferase, alkaline phosphatase, total bilirubin) and Electrolytes (potassium, magnesium and calcium). Approximately 2 ml per blood draw will be collected in a serum separator blood tube.

- **Glucose-6-phosphatase deficiency** Approximately 0.5 mL per blood draw will be collected in an EDTA (anticoagulant) tube will be evaluated by fluorescence (qualitative) tetsing, quantitative testing and with single nucleotide polymorphism (SNP) analysis. Approximately 0.5 mL per blood draw will be collected in an EDTA (anticoagulant) tube.

- **Malaria antibody titers** to malaria antigens (approximately 5 ml) in a serum separator tube.

- Volunteers will have 8 mL blood in sodium heparin tube drawn for malaria parasite culture and **in vitro drug resistance** testing before medication dosing and will be repeated at the time of diagnosis for any malaria recurrence.

- Volunteers will have 6 mL of blood drawn in EDTA tubes for analysis of **molecular markers** of malaria parasite drug resistance before medication dosing, and at 24, 48 and 72 hours after the first dose and the time of diagnosis for any malaria recurrence. From this 6ml of blood, 200-400 µl of blood will be spotted on filter paper and dried for stabilization of DNA and RNA, and **1 mL** will be aliquoted to the AFRMS laboratory for **PCR genotyping**. Filter paper spots will also be prepared during follow-up visits scheduled for days 7 through 42 from either the 3 mL of blood drawn for gametocyte PCR, or blood smear PCR correction on those days.

CONFIDENTIAL

- **Drug levels** of piperaquine and primaquine will be drawn (totaling 2 ml of blood for each drug) for pharmacokinetic profiles and *ex vivo* Plasmodium falciparum bioassay. Piperaquine levels will be drawn pre-dose and each dosing day of DP: 4, 24, 48, 52 and 72 hours. Given the long half-life, levels will also be drawn on Day 7, 14, 21, 28, 35, 42 and at any recrudescence. Primaquine levels will be drawn at 52 and 72 hours (after PQ dosing).

- **Membrane Feeding Assay** will be performed under SOP in concert with personnel from AFRIMS Department of Entomology. Two mLs in heparinized tubes will be drawn on Days 1, 4, 7, and 14, and within six hours, the assay will be performed at the MTFs.

- **Gametocyte PCR** will be conducted on 2.5 mL of blood drawn into Paxgene tubes or other appropriate tubes for RNA isolation on Days 1, 2, 3, 4, 7, 14 and at the time of any malaria recurrence. This PCR assay is a multiplex assay designed to detect presence of early or late stage gametocyte genes for both for *P. falciparum* and *P. vivax*. On Day 7, 14 an extra 0.5 mL of blood will be drawn with the 2.5 mL but this will be used for filter paper spots for molecular markers of resistance.

- **Urine pregnancy test**: Urine beta-HCG test. All female volunteers age 18-50 will undergo a pregnancy test on screening day and 42 day post-treatment follow-up. Pregnant women will not be eligible for entry into the study.

6.10.2 Specimen Preparation, Processing, Handling, and Storage

A. Malaria Microscopy - Stained thick and thin blood smears will be examined by two microscopists who are blinded to each other's results and to the treatment status of the study volunteer. Two blood smears will be made for every enrolled volunteer. Slide 1 will be stained immediately and examination of giemsa stained thick and thin smears. This slide will then be stored in a different box from slide 2, which will only be read there is a problem with the first slide.

Parasite densities will be calculated based on a count of parasites per 200 WBCs (thick film) or for low parasitemias (eg., 10 parasites/microliter), per 500 WBC or 5000 RBCs (thin film). Both asexual and sexual stages will be enumerated. A total of 200 oil immersion fields will be examined on the thick film before a blood smear is considered negative. The final count will be determined by taking the geometric mean of the two counts. In case of a difference in results (positive/negative; species diagnosis) between the two microscopists, the blood smear will be re-examined by a third microscopist blinded to the results of the first two readers and the treatment regimen, and the third reading will be accepted as the final result.

Malaria microscopy results will be confirmed using real-time PCR correction for the detection of *P. falciparum* and *P. vivax* using 18S ribosomal RNA (*18S rRNA*)

CONFIDENTIAL

genes unique to each species. Parasite DNA will be isolated from approximately 200-250 μL of venous or capillary blood collected in an EDTA microtube.

B. PCR Genotyping One mL of blood will be drawn on Days 1,2,3,4 and at recurrence. PCR genotyping of msp1, msp2 and GLURP genes will be performed to identify the unique fingerprint of the infecting parasite and any subsequent development of malaria after DP therapy in order to determine if it is a recrudescent infection or new infecting genotype.

C. Hematology for safety assessment will include the following:

- hemoglobin
- hematocrit
- red blood cell (RBC) count
- cell indices
- platelet count
- white blood cell (WBC) count and differential count
- polymorphonuclear leukocytes (neutrophils)
- lymphocytes
- eosinophils
- monocytes

D. Hemoglobin Typing: Blood will be drawn for baseline CBC in EDTA tube. 1.5 mL of whole blood will be washed with normal saline, pelleted and frozen at -20° C. The samples will batched and transported to a predesignated commercial lab, Thalassemia Research Center, Institute of Molecular Biosciences, Mahidol University, for hemoglobin typing analysis within 1 month using HPLC or other appropriate commercially available method. Molecular analysis will be performed at the commercial lab and/or AFRIMS in case the initial qualitative result is inconclusive.

E. Liver and Renal Function Tests for safety assessment will include the following:

- blood urea nitrogen
- creatinine
- total bilirubin
- aspartate aminotransferase
- alanine aminotransferase
- alkaline phosphatase
- potassium
- magnesium
- calcium

CONFIDENTIAL

F. G6PD Deficiency Testing

Volunteers will be assessed with both fluorescence (qualitative) and quantitative testing, with single nucleotide polymorphism analysis on enrollment. . Approximately 0.5 mL per blood draw will be collected in an EDTA (anticoagulant) tube.

Venous blood will be tested for qualitative G6PD activity using the fluorescent spot test method, as is recommended by the International Committee for Standardization in Hematology using commercially available kits (R&D Diagnostics Ltd, Greece). This method detects fluorescence of NADPH under long-wave (365 nm) UV light. Reduction of NADP to NADPH occurs in the presence of G6PD. The rate and extent of NADPH formation is proportional to G6PD activity. Normal samples fluoresce brightly, whereas deficient samples show little or no fluorescence.

Quantitative testing will be performed using an FDA-approved test kit (Trinity Biotech, Ireland) and results will be calculated based on same-day hemoglobin values from the complete blood count. Severe deficiency (WHO Class I or II) will be defined as 10% or less of the lower limit of normal activity (in G6PD activity units per gram of hemoglobin) established for the quantitative assay system. Subjects with severe deficiency will not be enrolled in the study as this is an exclusion criteria; Class III, IV and V deficiencies are permissible for enrolment.

For single nucleotide polymorphism (SNP) analysis, DNA will be extracted from ~ 0.5 ml of blood collected in EDTA, and the G6PD gene will be genotyped according to established methods (Fujii et al., 1984). The five SNPs to be evaluated at AFRIMS are: Mahidol (G487A), Viangchan (G871A), Chinese-5 (C1024T), Union (G1360T), Canton/Kaiping (G1376T/G1388T). Genotype data may be compared against existing databases, such as sequence data from other samples located in publicly accessible database(s) for example GENBANK. **No human genetic studies will be performed other than to assess G6PD genotypes and hemoglobin typing.**

G. Malaria Antibody Analysis. This will include but not be limited to testing for P. falciparum and vivax antibodies such as to MSP-1 (Merozoite Surface Protein 1) and/or MSP-3a antigens. Analysis will be conducted at baseline, at study end, and at any time the volunteer develops blood stage malaria (first infection or a recurrence). Antimalarial *P. falciparum* and *P. vivax* antibody levels will be measured in order to assess pre-existing and/or exposure-related antimalarial immunity. Samples will be analyzed by Enzyme-Linked ImmunoSorbent Assay (ELISA) and/or a chemiluminescence-based assay(s). Note that 'MSP-1' is used as a reference antigen; however, antibodies to multiple malaria antigens may be assessed under this protocol.

H. In vitro drug sensitivity. For *P. falciparum* monoinfection and mixed *P. falciparum* and other parasite species infections, approximately eight mL of heparinized blood will undergo in vitro drug sensitivity testing at AFRIMS using

CONFIDENTIAL

established methods (Noedl et al., 2004 and Noedl, 2005) with both fresh and cryopreserved cultures incubated against commonly used antimalarials in the region. Results using AFRIMS primary method (HRP-2 ELISA) vs. USAMRU-K and WRAIR's SYBR-Green assay will be compared. A portion of the specimen will be cryopreserved according to established procedures. Culture adaptation will be performed either at AFRIMS or other collaborating laboratories according to established methods (Trager and Jensen, 1997).

I. Molecular Marker analysis. To study genetic markers of resistance and population genetics of malaria in Cambodia, parasite DNA will be extracted from ~6 mL of WBC-depleted blood collected in EDTA and stored at -20ºC or below using a an appropriate DNA extraction kit. On Days 1, 2, 3, 4 filter paper blood spots will be prepared from the original 6mL sample, dried and stored at room temperature. Additionally, filter paper blood spots will prepared from venous blood drawn on Days 7 and 14 of follow up (from gametocyte PCR samples). DNA and RNA will be extracted from filter paper blood spots using an appropriate method. Samples will be analyzed by the University of North Carolina under Cooperative Research and Development Agreement with WRAIR (on file).

J. Membrane Feeding Assay. A laboratory colony of *An. dirus* established and maintained at the Department of Entomology, Armed Forces Research Institute of Medical Sciences (AFRIMS), in Bangkok, Thailand for more than 25 years will be used. This mosquito species is reared under laboratory conditions at ca. 26°C ± 2 and at a relative humidity of about 75% under a photo regime of 12:12 h (L:D). Fish food (C.P. Hi Pro®, Bangkok, Thailand) will be used to feed larvae on a regular basis. Mosquitoes will be provided cotton soaked with 10% multivitamin for an energy source until used in the experiments. The membrane feeding assay will be performed according to Department of Entomology SOPs. Briefly, two mL of blood will be drawn for mosquito membrane feeding on 1 (pre-dose), 4 (day 1 post primaquine), and days 7 and 14. Two hundred mosquitoes will be fed using a membrane feeding apparatus on fresh volunteer blood no more than 6 hours after blood draw. Of the mosquitoes determined to have taken a blood meal, half will be separated for oocyst evaluation by phase-contrast microscopy; or engorged mosquitoes will be separated from un-engorged mosquitoes and then incubated under appropriate environmental conditions. Half of them will be dissected after 9 days for oocyst evaluation and count the number with >/=1 midgut oocyst(s) by phase-contrast microscopy. Fifty percent of the remaining will be separated for preservation for molecular analysis. The remaining 50% of mosquitoes will continue to be incubated for approximately seven more days (day 16 post feeding), until sporozoite development in the salivary glands is complete. The mosquitoes will then be preserved in 95% Ethanol to be sent to UNC for parasite detection and genomic analysis.

K. **Gametocyte PCR.** A PCR assay for detection of *P. falciparum* and *P. vivax* sexual stage gametocytes will be performed at AFRIMS to evaluate both presence and stage of gametocyte development. For this assay 2.5 mL of blood will be

CONFIDENTIAL

collected in PAXgene Blood RNA Tubes and the RNA processed using PAXgene Blood RNA Kits (or similar collection tubes as appropriate). Filter paper blood spots may also be prepared for limited analysis. RNA templates from each extraction will be used for each reverse transcriptase PCR reaction to detect the early and late stage gametocyte genes.

L. Pharmacokinetics (PK) - Plasma samples for determining antimalarial drug levels of piperaquine and primaquine (2 ml of whole blood per blood draw) will be collected from all volunteers at the specified time points for analysis by high performance liquid chromatography with mass spectrometry (LC-MS) using departmental SOPs for bioanalytical chemistry analysis by LC-MS. A small aliquot of the plasma sample will be used to determine ex vivo antimalarial activity of the subjects blood using an established assay against *P. falciparum* (Noedl *et al*, 2004).

6.10.3 Specimen Labeling and Shipment

All specimens collected during the study will be labeled with the participants study ID number, date, and time collected. Clinical testing including diagnostics and volunteer safety labs will be performed at the study site (G6PD, hematology, renal, liver function, electrolytes and microscopy testing). Hemoglobin typing will be contracted to a commercial laboratory. Specimens that are stored prior to testing will be labeled to indicate the type of test(s) to be performed. Parasitology testing including parasite culture, *in vitro* resistance, and/or molecular characterization will be performed at AFRIMS reference lab in Cambodia or Thailand (parasite DNA and additional testing for volunteer safety assessment). Parasite genetic analysis on coded, de-identified samples will be performed at the University of North Carolina under an approved Cooperative Research and Development Agreement with AFRIMS and the WRAIR. The documents are currently on file with the WRAIR Office of Research and Technology Administration Office. A permit to ship samples outside Cambodia under this protocol will be obtained from the National Institute of Public Health, Cambodia (the responsible authority for shipping permits).

Specimens that cannot be analyzed on-site will regularly be shipped from the field to AFRIMS in Bangkok, and/or the University of North Carolina laboratories for further processing and analysis. In the event of remaining specimens, the University of North Carolina laboratories will return or destroy all specimens after all analytic methods have been performed and / or no more than three years after data analysis is complete.

6.10.4 Specimen Storage and Donation for Future Use

Following the completion of laboratory analyses as described in this protocol, any remaining specimens will be stored in a secure AFRIMS, AFRIMS contract facility or partner lab. During the course of the study, they will be regularly transferred to a secure facility. Specimen accountability will be maintained by the laboratory

CONFIDENTIAL

managers, and only study investigators and those named on the Delegation of Authority log will have access to the specimens. Samples that are unstable may be disposed of with permission of the principal investigators. The remaining specimens will be stored for approximately 20 years at the Armed Forces Research Institute of Medical Sciences in Bangkok. After the study is completed, residual specimens will only used for purposes outlined in the consent form unless permission for other analyses are granted by the respective IRBs. Volunteers will indicate on the consent form whether or not their samples may be stored and permission granted for future use.

6.11 Data Management

Clinical and laboratory data pertaining to drug efficacy will be collected and managed by AFRIMS Immunology and Medicine in collaboration with AFRIMS Epidemiology and Disease Surveillance, using guidelines developed by the World Wide Antimalarial Research Network, WHO and/or DoD GEIS (see Appendix D). Parasitological data may also be contributed to a central database at WRAIR and shared with partner labs. All deidentified data shared with WWARN or DoD GEIS will first be published as primary data by the Investigators, and public dissemination authorized by the appropriate host country officials before access to other research organizations to analyze, publish or disseminate data is granted.

Source data are all information, original records of clinical findings, observations, or other activities in a study necessary for the reconstruction and evaluation of the trial. Examples of these original documents and data records include, but are not limited to, hospital records, clinical and office charts, laboratory notes, memoranda, or evaluation checklists, pharmacy dispensing records, recorded data from automated instruments, copies or transcriptions certified after verification as being accurate and complete, microfiches, photographic negatives, microfilm or magnetic media, x-rays, and volunteer files and records kept at the pharmacy, at the laboratories, and medico-technical departments involved in the clinical study. The study site will maintain appropriate medical and research records for this trial until completion of the study, in compliance with Section 4.9 of ICH E6 GCP, and regulatory and institutional requirements for the protection of confidentiality of volunteers. Source data will be maintained under supervision of the principal investigator for at least 5 years after publication of data in per-reviewed journals. Source documents will be stored securely at AFRIMS Office in Cambodia under custody of the PI.

All data and medical information obtained about screened study volunteers will be considered privileged and confidential. Volunteers enrolling in the study will be issued a unique identification code (UIC), which will be used on all study files and clinical sample labels. Individually identifiable volunteer information other than the UIC will not be transcribed on other study documents to include laboratory sample labels, CRFs, nor will it be included in the presentation of study results.

CONFIDENTIAL

Screened volunteers will be assigned UIC consisting of SN (screening number) followed by four digit WRAIR IRB number assigned and a 3-digit number between 001 and 999 (e.g.1st volunteer: SN1877- 001, 2nd volunteer: SN1877-002, etc). The entry code for enrolled volunteers is the study name (TB), followed by four digit WRAIR IRB number assigned, with a 3-digit number from 001-150 (e.g. 1st enrolled volunteer: TB1877-001, 2nd enrolled volunteer: TB1877-002, etc.).

The key to the code and documents containing personal information will be kept in a secure location with access restricted to named AFRIMS and CNM study personnel under control of the Principal Investigator. All personal study volunteer data collected and processed for the purposes of this study will be managed by the investigators and those listed on the delegation of authority log with adequate precautions to ensure the confidentiality of those data, and in accordance with US law and/or applicable local laws and regulations where the requirements exceed those of US law. The study database will be maintained indefinitely by the Principal Investigators with password-protected access limited to listed investigators.

Monitors, auditors and other authorized agents, the United States Army Medical Research and Materiel Command, and the ethics committees approving this research will be granted direct access to the study volunteers' original medical records for verification of clinical trial procedures and/or data, without violating the confidentiality of the volunteers, to the extent permitted by the law and regulations. In any presentations of the results of this study at meetings or in publications, the volunteers' identity will not be revealed.

6.11.1 Source Documents

See section 6.11 above. Source documentation supporting the CRF will indicate the volunteer's participation in the study and will document the dates and details of study procedures, adverse events and volunteer status. Volunteer pre-existing conditions will be recorded in the appropriate sections of the source documentation if they are reported by the volunteer after participation has begun, and the Investigator notified immediately. Pre-existing conditions not reported by the volunteer at the time of enrollment may be grounds for terminating further study participation by the Investigator.

6.11.2 Overview of Case Report Forms

Appropriate data will be extracted from the source documents in this study onto case report forms. The AFRIMS study team will be responsible for completing the CRFs as data is collected. The investigator will ensure the accuracy, completeness, and timeliness of the data reported in the volunteer's CRF. CRFs will be submitted and approved by the IRBs of Record prior to initiation of the research.

All research data will be collected by the investigator or designee on source documents specifically designed for the purposes of conducting the study. Volunteer

CONFIDENTIAL

clinical and laboratory data for the purpose of providing medical care will be recorded in the appropriate clinic or hospital record using existing forms. Volunteer data necessary for analysis and reporting will be extracted on Case Report Forms specifically designed for that purpose.

6.11.3 Data Compilation

All data to be analyzed will be entered from the source documents as electronic Case Report Forms into a secure, access controlled database created and managed by the Investigative team at AFRIMS. Copies of the completed case report forms will be printed and retained by the study team. Data will be entered by trained study staff with 100% verification against the source documents by the study monitor. Any inconsistencies between the data sets will be corrected by the study team with a record kept of the corrections made. Edit checks will be implemented in the data entry panel to ensure data quality and accuracy. Responses to requests for further clarification of data recorded on the CRF will be answered, dated, and signed by the investigator and/or designee. Changes will be implemented in the database and the data review and validation procedures will be repeated as needed. All medication and adverse event information and textual comments will be proofread for consistency between the database and the source documents; the database will be corrected appropriately. The study database will be maintained at AFRIMS by the Investigative team with password-protected access limited to authorized study team members.

6.11.4 Disposition of Data

The case report forms, study documentation and a copy of the final report will be stored in an access-controlled place in the contracted archives of AFRIMS. All data will be retained according to ICH guidelines by the Investigators for 5 years at AFRIMS office in Cambodia. After 5 years CNM and RCAF will be consulted regarding data disposition or continued storage of raw data, which will be at additional cost to these entities.

6.12 Adverse Events

An adverse event is any untoward medical occurrence in a volunteer or clinical investigation volunteer administered a pharmaceutical product and which does not necessarily have a causal relationship with this treatment. An AE can therefore be any unfavorable and unintended sign (including an abnormal laboratory finding), symptom, or disease temporally associated with the use of a medicinal (investigational) product, whether or not related to the medicinal (investigational) product.

CONFIDENTIAL

6.12.1 Collecting Adverse Events

Volunteers treated for malaria with study drug will be carefully monitored for the development of adverse events. For the purposes of this study, adverse events will be assessed and documented from the beginning of study drug administration until discharged from the study, and relationship to study drug assessed. Any evidence of adverse event, syndrome or diagnosis occurring post-consent but before drug administration will be assessed and documented as “Preexisting”. This information will be obtained in the form of open-ended (non-leading) inquiries and from signs and symptoms noted during clinical encounters, observations by study staff, spontaneous reports from volunteers and other sources as appropriate. Specific adverse events will not be solicited in this study. Volunteers will be able to contact study staff through assigned unit liaisons in the event of an emergency.

Study investigators will attempt to establish a diagnosis of the event based on signs, symptoms, and/or other clinical information. Where possible, the clinical diagnosis will be documented as the AE/SAE rather than the individual signs/symptoms. Each adverse event will also be described by its duration (start date, time and duration), an assessment of its cause (e.g. coexisting disease, concomitant medication, or others), its relationship to investigational product (not related, unlikely, possibly, probably, definitely), and whether it required specific therapy.

The investigator will also make an assessment of severity for each AE reported during the study. The assessment will be based on the investigator’s clinical judgment. An AE that is graded as severe should not be confused with a serious adverse event (SAE). The severity of each adverse event must be recorded as 1 of the choices on the following scale:

- <u>Mild</u> - No limitation of usual activities
- <u>Moderate</u> - Some limitation of usual activities
- <u>Severe</u> - Inability to carry out usual activities

An adverse event (AE) temporally related to participation in the study will be documented whether or not considered to be related to the test article. This definition includes intercurrent illnesses and injuries and exacerbations of preexisting conditions. For each adverse event, the relationship to the study drug must be recorded as 1 of the choices on the following scale:

- <u>Definite</u> - Causal relationship is certain (ie, the temporal relationship between drug exposure and the adverse event onset/course is reasonable, there is a clinically compatible response to dechallenge [a rechallenge procedure may be used, if necessary], other causes have been eliminated, and the event is definitive pharmacologically or phenomenologically)

CONFIDENTIAL

- Probable - High degree of certainty for causal relationship (ie, the temporal relationship between drug exposure and the adverse event onset/course is reasonable, there is a clinically compatible response to dechallenge [rechallenge is not required], and other causes have been eliminated or are unlikely)

- Possible - Causal relationship is uncertain (ie, the temporal relationship between drug exposure and the adverse event onset/course is reasonable or unknown, dechallenge/rechallenge information is either unknown or equivocal, and while other potential causes may or may not exist, a causal relationship to the study drug does not appear probable)

- Unlikely - Not reasonably related (ie, while the temporal relationship between drug exposure and the adverse event onset/course does not preclude causality, there is a clear alternate cause that is more likely than the study drug to have caused the adverse event)

- Not Related - No possible relationship (ie, the temporal relationship between drug exposure and the adverse event onset/course is unreasonable or incompatible, or a causal relationship to study drug is implausible)

6.12.2 Documenting Adverse Events

All adverse events will be recorded based on their frequency, severity, and relationship to study medication in accordance with current AFRIMS/WRAIR SOP. These indices of safety and tolerability among treatment groups will be compared using each volunteer as the unit of analysis. Adverse events will be documented in the volunteer source documents and case report forms. Adverse events will be assessed and recorded by study investigators or their designees. An Investigator will review all causality and severity assessments, with final review and determination by the Principal Investigator if uncertainty remains.

6.12.3 Expected Adverse Events

All AEs occurring during the course of the clinical trial, defined as from the moment of first antimalarial treatment administration until discharge from the study, will be collected, documented, and graded by study investigators. Symptoms present at enrollment will not be classified as AEs, but any new symptoms or signs occurring after this time would constitute adverse events.

For this study, AEs will include events reported by the volunteer, as well as clinically significant abnormal findings on physical examination or laboratory evaluation. A new illness, symptom, sign or clinically significant clinical laboratory abnormality or worsening of a pre-existing condition or abnormality is considered an AE. Stable chronic conditions, such as arthritis, which are present prior to clinical trial entry and do not worsen are not considered AEs.

CONFIDENTIAL

Adverse events that occur will be treated as clinically indicated where appropriate. The most likely adverse event that will occur during the study is malaria infection. Malaria infection or reoccurrence will be actively sought by the study team, and all suspected cases will be referred immediately for further evaluation and treatment as described in the protocol. It is expected that active detection and treatment by a dedicated team will lead to earlier diagnosis and initiation of appropriate therapy, potentially reducing the rate of more severe illness.

6.12.4 Serious Adverse Events and Unanticipated Problems

A serious adverse event (SAE) is defined as any adverse experience occurring during study participation that results in any of the following outcomes: death, a life-threatening adverse drug experience, inpatient hospitalization or prolongation of existing hospitalization, a persistent or significant disability/incapacity, or a congenital anomaly/birth defect. Important medical events that may not result in death, be life-threatening, or require hospitalization may be considered a serious adverse drug experience when, based upon appropriate medical judgment, they may jeopardize the patient or volunteer and may require medical or surgical intervention to prevent one of the outcomes listed in this definition.

An SAE will be defined in this study as any untoward medical occurrence regardless of cause or relationship to study drug that:

- Results in death.
- Is life-threatening. Any adverse experience that places the volunteer, in the view of the investigator, at immediate risk of death from the reaction as it occurred (i.e., it does not include a reaction that, had it occurred in a more serious form, might have caused death).
- Requires in-patient hospitalization or prolongation of existing hospitalization (excluding any hospitalization or inpatient observation period required by the study for the period required to treat malaria and any associated co-morbidities).
- Results in persistent or significant disability or incapacity.
- Is a congenital anomaly/birth defect.
- An event that requires urgent medical intervention to prevent permanent impairment or damage.
- Important medical events that do not result in death, are not life-threatening, or do not require hospitalization may be considered serious adverse events when, based upon appropriate medical judgment, they might jeopardize the volunteer and might require medical or surgical intervention to prevent one of the outcomes listed above.

CONFIDENTIAL

Unanticipated Problems Involving Risks to Subjects or Others: Based on Federal regulations 45 CFR 46.103(b)(5)(i) and 21 CFR 56.108(b)(1)

DEFINITION: “An unanticipated problem is defined as any incident, experience, or outcome that meets all of the following criteria:

1. Unexpected (in terms or nature, severity, or frequency) given the approved research procedures and the subject population studied;
2. Related or possibly related to a subject’s participation in research; and
3. Suggests that the research places subjects or others at greater risk of harm (physical, psychological, economic, or social harm) than was previously known or recognized.

Examples of unanticipated problems include (but are not exclusive to) exposure to HIV or other infectious disease due to an unintentional needle stick, disclosure of protected health information, occurrences of breaches of confidentiality, destruction of study records, unaccounted for study drug, etc.”

An unexpected or unanticipated event involving risks to volunteers or others is one that is not described as a risk with respect to nature, severity, or frequency in the protocol and/or informed consent form. An unexpected adverse event is further defined as any adverse drug effect, the specificity or severity of which is not consistent with that which has been previously reported in the current published literature, or described in the study documents.

6.12.5 Adverse Event Reporting

Expected adverse events will be reported on a routine basis to the responsible IRBs by the investigator as part of scheduled Continuing Review Reports as stipulated by the IRB.

Serious Adverse Events should be immediately reported (within 48 hours) to the NEHCR, Cambodia National Ethics Committee for Health Research (Tel.: 855 23 880-345, Fax: 855 23 880-346, E-mail: research03@nchads.org) as well as by telephone (301-319-9940), fax (301-319-9961) or email (wrairhspb@amedd.army.mil) to the WRAIR IRB, thru the WRAIR HSPB, that meet the following criteria as soon as the principal investigator becomes aware of the event, and then must be followed-up in writing within 10 working days from knowledge of the event:

i. **SERIOUS** (i.e., death, a life-threatening adverse experience, inpatient hospitalization or prolongation of existing hospitalization, a persistent or significant disability or incapacity, or a congenital anomaly or birth defect [21 CFR 312.32(a)]), **and**

ii. **UNANTICIPATED** (An unanticipated event is any adverse experience where the nature, severity or frequency is not identified in the investigator brochure or described in the protocol. Events which are

CONFIDENTIAL

already cited in the investigator brochure or protocol are not unanticipated and do not have to be reported to the WRAIR IRB, except in the continuing review report), **and**

iii. **RELATED** to the study design, procedures, or drug/device (possibly, probably or definitely related, or undetermined/unknown). If the adverse experience/event is clearly not related to the study drug, device, procedures, or washout process, it would not represent a risk to other subjects in the research and, therefore, does not have to be reported to the WRAIR IRB

Unanticipated problems involving risks to subjects or others should be promptly report (within 48 hours of the PI becoming aware of the problem) by telephone (301-319-9940), fax (301-319-9961) or email (wrairhspb@amedd.army.mil) to the WRAIR IRB, thru the WRAIR HSPB, and then must be followed-up in writing within 10 working days from awareness of the problem.

All safety reports for events that are both serious and unexpected at a minimum will include Volunteer identification number and initials, volunteer's ages, gender and ethnicity, test article and dates of administration, signs/symptoms and severity, date of onset, date of resolution or death, relationship to the study drug, action taken, concomitant medication(s) including dose, route and duration of treatment, and date of last dose.

Research monitors are required to review all unanticipated problems involving risks to subjects or others, serious adverse event reports, unanticipated adverse device effects, and all subject deaths, and provide an unbiased written report of the event promptly to the NEHCR, Cambodia National Ethics Committee for Health Research (Tel.: 855 23 880-345, Fax: 855 23 880-346, E-mail: research03@nchads.org), and as well as by telephone (301-319-9940), fax (301-319-9961) or email (wrairhspb@amedd.army.mil) to the WRAIR IRB, thru the WRAIR HSPB. The Research monitor will then submit written reports within 10 working days to the WRAIR IRB and to the National Ethical Committee for Health Research, Cambodia.

6.12.6 Follow-up of Adverse Events

All AEs regardless of severity will be followed by study investigators until satisfactory resolution. Resolution could include a classification of ongoing if the event is stabilized with no further change expected. The investigator will ensure that follow-up includes any supplemental investigations as may be indicated to elucidate the nature and/or causality of the AE or SAE. This may include additional laboratory tests or investigations, histopathological examinations, or consultation with other health care professionals.

CONFIDENTIAL

Dropout rates and reasons for dropping out will be reported. If a study volunteer withdraws from the study or if an investigator decides to discontinue the volunteer from the study because of a SAE, effort will be made to ensure the volunteer has appropriate medical follow-up. Monitoring will continue where possible and appropriate in order to determine whether the problem prompting hospitalization has resolved or stabilized with no further change expected, or is discovered to be clearly unrelated to study drug, or progresses to death. The Investigator/clinical staff will report the follow-up for serious adverse events as noted above.

After discharge from the study, any treatment-related adverse events classified as “probable” or “definite” in relation the study drug, will be followed to resolution where possible. All SAEs will be followed until satisfactory resolution or until the Principal Investigator (with agreement of the research monitor) deems the event to be chronic or the volunteer to be stable.

A post-study AE/SAE is defined as any event that occurs after the volunteer has been discharged from the study. Investigators are not obligated to actively seek AEs or SAEs in former study participants. However, if the investigator learns of any SAE, including a death, at any time after a volunteer has been discharged from the study, and he/she considers the event reasonably related to the study, the investigator will promptly notify any IRB.

6.13 Criteria for Discontinuation or Withdrawal of a Subject

Any volunteer may be discontinued from the study at any time at the discretion of the Principal Investigator or designee, research monitor, a consulting clinical physician, or a responsible IRB if he/she feels it is in the best interest of the volunteer or if in the judgment of the investigator continuing in the study would be harmful and/or inappropriate for the volunteer (e.g. volunteers not tolerating the study treatment, development of SAEs or if a volunteer cannot be followed thereby not permitting adequate safety assessment). Any volunteer who is terminated due to an SAE or determined to have an unexpected AE will be reported to the research monitor for review. See section 6.13.1 below for pre-specified halting criteria. Any volunteer who is discontinued or who withdraws from the study will be asked to come in for clinical and laboratory assessments required to ensure volunteers safety and to complete discharge procedures.

The NEHCR and the WRAIR IRB will be notified when a volunteer is withdrawn from the study as part of the continuing review report, unless withdrawal is the result of an SAE.

CONFIDENTIAL

6.13.1 Halting Rules Criteria

Since primaquine administration is a onetime (45 mg) dose only, any volunteer with mild to moderate G6PD deficiency who experiences grade 3 hemolysis after this dose of primaquine will be monitored closely according to protocol procedures and any treated with any necessary interventions by the study investigators and/or the research monitor for the volunteer's safety.

If more than 2 subjects with mild to moderate G6PD deficiency are found to have grade 3 hemolysis following treatment with primaquine for antirelapse therapy, further treatment with primaquine will be suspended for all G6PD deficient subjects enrolled in the study.

Volunteers observed to have sustained QTcF prolongations greater than 500ms (grade 3) on more than one EKG study at least 15 minutes apart where this represents a significant increase compared to screening QTcF will be evaluated by the Principal Investigator. If it is determined that the QTcF prolongation is study-drug related, and not due to confounding factors to include fever, tachycardia, concomitant ingestion of other QT prolonging medications, or electrolyte deficiencies, any remaining DHA-piperaquine treatment will be halted, and the patient switched to an alternative drug to complete an adequate course of therapy as described under Section 6.14, Rescue Treatment. All such cases, regardless of relatedness, will be reported to the DSMB as described under the DSMB Charter, including those who may have completed therapy prior to the observation of grade 3 QT interval prolongation (see Appendix F). All volunteers with grade 3 QT interval prolongation will continue safety follow-up for the duration of the study.

6.14 Rescue Treatment for Malaria Infection

The requirement for rescue treatment will be based on investigator clinical judgment. However, failure to respond adequately to DP will include the following: development of danger signs (e.g. impaired consciousness, convulsions, respiratory distress) or severe malaria in the presence of parasitemia; and parasitemia on Day 2 higher than Day 1. The presence of both asexual parasitemia and fever (tympanic temperature > 38˚C) on Day 3 is not necessarily an indication for rescue treatment as long as parasite counts continue to trend downward, and the volunteer is without danger signs as outlined in the National Guidelines. Per protocol, volunteers will remain under direct observation until both parasitemia and fever have cleared.

Recent AFRIMS clinical studies in this region have demonstrated that the majority of malaria volunteers with both fever and parasitemia at 72 hours who continue their antimalarial treatment will go on to clear parasites and fever and remain free from recurrence up to 42 days (Noedl, 2010; and Bethell, 2011), and it is important to capture this outcome. Treatment of volunteers who fail the primary treatment

CONFIDENTIAL

regimen with worsening symptoms, fever, and/or parasitemia after day 4 will be given malaria rescue therapy in accordance with current National Treatment Guidelines. Volunteers who develop worsening or unexplained symptoms not otherwise attributable to malaria at any time will be evaluated for alternative diagnoses.

Subjects with signs of severe malaria will be excluded and referred for immediate treatment. Stable subjects with high parasitemias up to 200,000 may be treated under this protocol as with similar approved protocols in the past including WR 1396 (ARC2) and WR 1737 (Prophylaxis Pilot Study). Patients presenting for treatment with parasitemias between 1,000-200,000 who are clinically stable and can take oral medications will not be excluded.

6.15 Criteria for Study Termination

See section 6.13 above for individual participation and/or study drug halting and termination. If more than 3 volunteers are determined to have sustained grade 3 QTcF interval prolongation attributable to study drug by the DSMB, the DSMB may recommend that the study be halted. The Investigators may terminate the study at any time if it is determined that continuing the study would pose an undue risk to the safety of volunteers.

If more than 2 subjects with mild to moderate G6PD deficiency are found to have grade 3 hemolysis following treatment with primaquine for antirelapse therapy, further treatment with primaquine will be suspended for all G6PD deficient subjects enrolled in the study. However, the overall study will not be halted, and study visits for all volunteers other than primaquine dosing for those with G6PD-deficiency will continue for all volunteers as scheduled.

6.16 Quality Control and Quality Assurance

AFRIMS maintains approved SOPs/SSPs that govern QC/QA procedures that will be followed during the course of this study.

CONFIDENTIAL

7 STATISTICAL METHODS

7.1 Statistical Procedures

Because this is a surveillance study, data will be analyzed on a continuous basis with generation of an annual report to the National Malaria Control Program of results. This study aims to monitor efficacy of a 3-day fixed-dose course of DHA-piperaquine for uncomplicated P. falciparum malaria over a three year period along the Thai-Cambodia border. Any loss of efficacy, clinical or parasitologic, or increase in molecular determinants of drug resistance, will be crucial for updating or adjusting national malaria treatment guidelines by the CNM. A sub-analysis for overall 42-day efficacy of DP as compared to DP/PQ will also be performed. The potential reduction in gametocytes by a onetime dose of primaquine is exploratory in nature and statistics will be descriptive in nature. See Section 8 for detailed data analysis procedures.

Volunteers developing malaria will randomized to either 45mg single dose primaquine or no primaquine treatment on day 3. Effects on the sexual stage gametocytes will be explored using a combination of light microscopy, PCR genotyping to distinguish early and late stage gametocytes, and a mosquito membrane feeding assay to determine malaria oocyst prevalence in the mosquito. There are no statistical assumptions or power calculations for this analysis as it remains exploratory – little data is available on which to develop assumptions.

7.1.1 Sample Size Estimation

The statistical analysis of 42-efficacy of the 3-day course of DP will be based around a 1-sample proportion (proportion that fail treatment). The primary end-point for sample size purposes will be 42 day efficacy of DP for uncomplicated *P. falciparum*. Each year, approximately 50 subjects will be enrolled over the 3 year period (total n estimated at 150 evaluable subjects). If the point estimate for 42 day efficacy is 94%, the 95% confidence interval for the annual estimate of true efficacy will be approximately 89-97% (n = 150).

Approximately 150 evaluable volunteers, 50 during each year of the study, will be enrolled. This is felt to be an appropriate target enrollment based on early epidemiologic data gathered from existing passively collected government sources, and small active case detection exercises conducted during site assessment activities indicate that the malaria attack rate will average 5-10% per month. Estimates of treatment cure rates and 95% CIs (exact) will be reported on at least an annual basis until the surveillance activity ceases or changes substantially (eg, a

CONFIDENTIAL

new first-line ACT is introduced, and there is no longer an interest by Cambodian authorities in monitoring DHA-piperaquine efficacy).

7.1.2 Randomization and Stratification

All volunteers enrolling with *P. falciparum* or mixed infection malaria will receive 3 days of DP therapy but then will be randomized into the two open label treatment arms using block randomization with a block size of two. Data may be stratified post-hoc based on demographic and/or other variables, but stratification is not part of the primary analysis.

7.1.3 Populations for Analysis

All volunteers with a diagnosis of uncomplicated malaria who receive at least one dose of test article during the Treatment Study will be included in the efficacy database for Primary Endpoint analysis (Intention to Treat). The per protocol analysis population will include all those volunteers who completed the full prescribed treatment course of DHA-piperaquine as well as 42 day follow-up. However, volunteers lost to follow-up that do not complete 42 days worth of assessments will be excluded from the per protocol efficacy analysis, but included in a modified intention to treat analysis. All volunteers with at least one follow-up assessment will be included in the safety analysis, pharmacokinetic analysis, and MSP-1 antibody titer analysis. All parasitologic data will be included in the parasitologic analysis. The safety analysis database will include those volunteers in the set of randomized volunteers who receive at least one dose of study drug.

7.1.4 Deviations from the Statistical Plan

Major deviations from the statistical plan such as changes in treatment regimens or number of enrollees along with the reasons for the deviations, will be described in protocol amendments, the complete statistical plan, the clinical study report, and/or any combination of these, as appropriate.

CONFIDENTIAL

8 DATA ANALYSIS

8.1 General Considerations – Data Analysis

Planned data analyses are as follows.

(1) Assess/test the efficacy (recurrence rates at 42 days) of a 3-day course of DP as compared to DP followed by one time dose of PQ

(2) Determine the effect of onetime dose of primaquine on presence of gametocytemia and transmissibility of infection to Anopheles mosquitoes

Primary endpoint treatment efficacy is defined as PCR-corrected parasitological cure of malaria at 42 days after starting therapy. Efficacy against all blood stage malaria infection will also be classified according to WHO malaria treatment outcome classifications adapted for the purposes of clinical research (WHO 2009) as a secondary endpoint. Note however that clinical management of these individuals will be based on the best clinical judgment of the investigators and informed by current National Treatment Guidelines in Cambodia (see Appendix A).

8.2 Efficacy Study Analysis

Treatment Outcome	Symptoms and Signs
Early treatment failure	- Development of danger signs or severe malaria on days 1-3 in the presence of parasitemia.<br>- Parasitemia on day 2 higher than the day 0 count irrespective of temperature.<br>- Parasitemia on day 3 with tympanic temperature $\geq$38.0^{o}C.<br>- Parasitemia on day 3 that is $\geq$25% of the count on day 1.
Late treatment failure:	
- Late clinical failure	- Development of danger signs or severe malaria after day 3 in the presence of parasitemia, without previously meeting any of the criteria of ETF.<br>- Presence of parasitemia and tympanic temperature $\geq$38.0^{o}C (or history of fever) on any day from days 4-42, without previously meeting any of the criteria of ETF.
Late parasitological failure	- Presence of parasitemia on any day from days 7-42 and tympanic temperature <38.0^{o}C, without previously meeting any of the criteria of ETF or LCF.
Adequate clinical and parasitological response	- Absence of parasitemia on day 42 irrespective of tympanic temperature without previously meeting any of the criteria of ETF, LCF or LPF.

CONFIDENTIAL

Efficacy at Day 42 will be based on microscopy of thick/thin blood films by expert microscopists blinded as to treatment allocation and clinical data of each volunteer following AFRIMS SOP. The primary endpoint (cure) for the treatment portion of the study is defined as non-recurrence of malaria (within 42 days) following treatment as detected by PCR-corrected microscopy. Cure rates (exact 95% CIs) will be calculated. Fisher's exact test (two-sided) will be used to test the null hypothesis of no difference in cure (fail) rates. A p-value < 0.05 (two-sided) will be considered statistically significant. As supporting analysis, a log- rank test will be performed to compare time to treatment failure in the two treatment regimens. The efficacy analyses will be done for the intent to treat and per protocol population.

Demographic, epidemiological, and laboratory data will be summarized at baseline and for any data collected at follow-up. Appropriate univariate statistics will be calculated to summarize and compare the range, location (means, medians, etc), and variability of numerical data. Geometric means will be calculated for antibody titer data, and all non-normally distributed data. Graphical summaries (e.g. box plots, histograms) will be used to describe and compare distributions of numeric variables. Frequency tables will be used to summarize distributions for discrete data. Confidence limits (95%) for means, geometric means and proportions will be calculated.

All clinical and laboratory data related to secondary endpoints (Section 5.2) will be summarized and compared in the two regimens. T-tests and chi-square tests will be used to assess the statistical significance of differences in two means (possibly log-transformed) or proportions. Confidence limits (95%) for means, geometric means and proportions will be calculated. Time to event data (e.g. fever and parasite clearance time) will be summarized using Kaplan-Meier plots. The log-rank test will be used to assess the statistical significance of difference between treatment groups. The time required to achieve a reduction in parasite density of 50%, 90%, and to undetectable levels will be analyzed using Cox proportional hazard modeling. Curve fitting will be used to interpolate results for calculation of parasite clearance and parasite density reductions

8.3 Pharmacokinetic Analysis

Drug levels (piperaquine) at each of the time points will be expressed as means and 95% confidence intervals if the data are normally distributed, and compared using parametric tests. If not normally distributed they will be expressed as medians and the range and interquartile range given. Comparison will be by non-parametric tests. Standard pharmacokinetic parameters including C_{max}, T_{max}, $T_{1/2}$ and AUC will be calculated using WinNonLin and/or other appropriate microcomputer software packages. Exploratory analyses will be undertaken to describe the relationship between plasma concentration and the effect of DP treatment on pharmacodynamic variables over time. If the data allow, an attempt will be made to characterize the relationship using modeling.

CONFIDENTIAL

8.4 Parasite drug resistance in vitro

For in vitro data from patients with *P. falciparum* infections, inhibitory concentrations at 50% (IC50) and 90% (IC90), the principal measures of drug sensitivity, will be estimated by non-linear regression analysis of the raw data obtained from the ELISA plate reader or the liquid scintillation counter (Noedl 2002). ICs and other continuous variables will be summarized using geometric means with 95% confidence intervals. Comparison of activity will be done by comparing individual ICs by Mann Whitney U-test analysis. Comparison of results obtained using different methods will be done by correlation analysis and Bland-Altman plots (Bland and Altman 1995).

8.5 Molecular Markers of Parasite Resistance

For studying genetic markers of resistance and population genetics of malaria in Cambodia, parasite DNA extracted from human white blood cell-depleted blood samples and filter paper blood spots will be subjected to genotyping using various platforms including but not limited to direct DNA sequencing, next-generation DNA sequencing, real-time PCR, other molecular biology tools and DNA and RNA chip technologies, to identify specific parasite variants or genetic loci associated with resistance. Parasite DNA will also be extracted from the mosquitoes used in the membrane feeding assay and will be subjected to similar genetic and molecular testing. RNA stabilized on blood spots will be used to assess stage-specific asexual and sexual parasite gene expression.

The exact methods of testing and assays used may vary from what is outlined here as technologies in genetics and genomics are rapidly advancing. Two primary analyses will be performed as part of this study; however, additional studies of parasite drug resistance and population genetics may be conducted using these samples and the techniques outlined above. The two primary analyses will include: 1) studying the transmissibility of drug resistant parasites from humans to mosquitoes and 2) evaluating for within-host selection of drug resistance.

1) Transmissibility of genetic variants will be assessed from all volunteers regardless of PQ or placebo receipt. Using Massively Parallel Pyrosequencing (MPP) the complete distribution of parasite variants (including their frequency) in samples can be determined. Thus the progress of specific variants through the transmission cycle to the mosquito can be followed to determine if any selection or genetic bottlenecking occurs. For each volunteer, parasite DNA extracted from blood samples, mosquito midguts and mosquito salivary glands collected at specified time points will be PCR-amplified using primers specific for the central variable region of merozoite surface protein 2 (*msp2*) and the drug resistance gene pfmdr1. As the starting material from the midguts and salivary glands will be limited, whole genome amplification (WGA) will be conducted prior to amplicon preparation. Fidelity of genome amplification will be verified using analysis of 20 microsatellite markers on

CONFIDENTIAL

the original genomic and amplified DNA. Depending on the yield of WGA, only amplicons from individual mosquitoes with high oocyst loads may be able to be generated. Initially, individual mosquitoes will be sampled, and the number of variants within each mosquito will be compared to the mosquito lot for each volunteer. After this type of sampling is done on a few prototype patients, all mosquitoes fed on each patient will likely be combined and the whole lot analyzed for better yield and cost-saving/efficient purposes. The amplicon libraries will be sequenced using the 454 sequencer at the Microbiome Core Facility located at the University of North Carolina (UNC). A goal of 2,000 reads per sample will allow detection of variants as low as 2.5% of the parasite population with high precision. Data analysis will be done using a new bioinformatic pipeline for haplotype building developed at UNC. Changes in parasite diversity may also be evaluated using microsatellite mapping, linkage disequilibrium calculation, phylogenetic characterization, and other molecular analyses as developed on isolated parasites from both human and mosquito.

2) Within-host selection for drug resistance will be evaluated using similar techniques as described above (MPP) to track genetic variants longitudinally during the course of treatment. An increase in the relative frequency of one variant compared to a second after one replicative cycle would suggest that the one over-represented was more fit in the presence of drug and therefore may be more drug tolerant. Additional analysis of these tolerant phenotype parasites by genomics tools (e.g. NGS, DNA and RNA chips) may help identify novel loci in the genome associated with drug resistance. In order to do this, selection coefficients for variants up-selected by DHA and/or piperaquine based on in vitro resistance profiles will be defined. Using data on parasite density and change in parasite frequency, selection coefficients will be determined. This will be the first time these will be determined in vivo. We will also model the in-host dynamics of selection of DHA resistant parasites in vitro using culture adapted parasites.

These samples may be used in the future to address other issues related to drug resistance and parasite genetic structure in Cambodia. For example, microsatellite analysis could help determine the genetic origin of any novel resistance loci (or known resistance loci) identified in the parasites during the trial. In this case, the heterozygosity and variance in allele size could be calculated, as well as the number and frequency of alleles at each microsatellite locus. He and Fst would be calculated between all pairs of clusters as previously described (Vinayak, 2010). Finally, genetic analysis may also be conducted at AFRIMS (Immunology) and/or WRAIR (Malaria Research Program) including, but not limited to, assessment of gene copy numbers of pfcrt (chloroquine resistance), pfdhfr (folate resistance), pfmdr1 (multi-drug resistance), and pfcytbc1 (atovaquone resistance), and other markers as appropriate.

CONFIDENTIAL

8.6 Safety Analysis

The overall safety and tolerability of DHA-piperaquine treatments will be assessed throughout the study by evaluating adverse events and the following additional safety variables:

- Clinical laboratory tests (liver function, renal function, and hematology)
- Vital signs
- Physical exam findings
- Cardiac safety as determined by electrocardiogram (EKG)

For continuous variables, descriptive statistics (n, mean, standard deviation, median, minimum and maximum) will be provided. For categorical variables, volunteer count and percentage will be provided. Descriptive summaries of serious adverse events, volunteer discontinuations due to adverse events, and potentially clinically significant abnormal values (clinical laboratory or vital signs) will also be provided. EKG findings will be analyzed, including corrected QTc intervals using Bazett's and Fridericia's formulae. Adverse events will be attributed to the treatment regimen corresponding to the last dose administered.

Adverse events (AE) will be expressed as percentages and compared with chi-square tests. AE rates are expected to be small and the study is not powered to detect differences in AE rates. Because of the large number of statistical tests, p-values will not be used to assess "statistical significance", but to flag differences in AE rates. Differences in AE rates will be flagged if $p < 0.05$ and identified as possibly clinically important if $p < 0.01$. Correlations between clinical and laboratory parameters will be explored and represented graphically. Other exploratory analyses may be carried out.

8.7 Interim Analysis

Results will be analyzed on a continuous basis.

CONFIDENTIAL

9 ETHICAL CONSIDERATIONS

The investigator will ensure that this study is conducted in full conformity with the International Conference for Harmonization Good Clinical Practice (ICH-GCP) regulations and guidelines, whichever affords the greater protection to the volunteer.

9.1 Informed Consent

Freely given informed consent will be obtained from every volunteer prior to study participation. Informed consent will take place before any study specific procedure, prior to the initiation of non-routine study-related tests, and prior to administration of study drug. Signed and dated, informed consent will be obtained from each volunteer in accordance with GCP and with local regulatory and legal requirements. The completed informed consent form must be retained by the investigator as part of the study records and a copy will be provided to study volunteers. The investigators, or a person designated by the investigators, will fully inform the volunteer of all pertinent aspects of the study including the written information giving approval by the IRB/IEC. Neither the investigator, nor the trial staff, will coerce or unduly influence a volunteer to participate or to continue to participate in the study.

In obtaining and documenting informed consent, the investigators will comply with the applicable regulatory requirement(s), and will adhere to GCP. Prior to the beginning of the study, the investigators will have the IRB/IEC's written approval/favorable opinion of the written informed consent form and any other written information to be provided to volunteers.

The written informed consent form and any other written information to be provided to volunteers will be revised whenever important new information becomes available that may be relevant to the volunteer's consent. Any revised written informed consent form, and written information will receive the IRB/IEC's approval/favorable opinion in advance of use. The volunteer will be informed in a timely manner if new information becomes available that may be relevant to the volunteer's willingness to continue participation in the study. The communication of this information will be documented. This may be accomplished by repeating the consent process with the revised consent form with attention given to the changes, or it may be done using an addendum consent that states the revision or new information. The new document will be signed, placed in the study record, and a copy given to the volunteer. New volunteers enrolled in the study will be consented with the most recent approved consent form.

9.2 Volunteer Identification and Confidentiality

All personal study volunteer data collected and processed for the purposes of this study will be managed by the investigators and his/her staff with adequate

CONFIDENTIAL

precautions to ensure the confidentiality of those data, and in accordance with applicable national and/or local laws and regulations on personal data protection. Volunteers will not be identified in any presentation of the results.

This study will not involve the collection of data on sensitive matters such as sexual behavior or criminal activities. No HIV or human genetic testing will be performed on any samples collected during this study other than to assess G6PD genotypes and hemoglobin typing. This protocol does not involve audio or videotaping of research volunteers. All volunteer records and CRFs will be carefully designed to limit the personal information to be acquired to that which is essential. Data that could reveal a volunteer's identity will be stored in files accessible only to authorized staff. As early as feasible, the data will be coded to remove identifying information.

9.3 Data Management

The database generated by this study will contain information collected through CRFs and laboratory data It will be created in collaboration with the AFRIMS Department of Epidemiology and Surveillance (EDS). Clinical and laboratory data pertaining to drug efficacy will be managed by AFRIMS Immunology and Medicine using guidelines and data management tools developed by the World Wide Antimalarial Research Network (WWARN). WWARN was constructed based on the need for a comprehensive, global surveillance system "to identify new foci of [artemisinin] resistance, develop tools to track its spread and provide to the malaria community the information needed to contain resistance....WWARN has built a secure web-based platform that allows researchers to share data on the drug responses of individual patients or parasites, which are transformed into comparable standard formats" (www.wwarn.org). See Appendix D for the WWARN database recommendations and analyses. WWARN also uses the WHO Guidelines for drug efficacy analysis listed in Section 8.1. Data management (source, CRFs etc) for elements information not collected according to WWARN guidelines will be developed by AFRIMS Immunology and Medicine. Parasitological data will also be contributed to a central database at WRAIR and shared with partner labs. No individually identifiable information will be included in the database, and the database will be password protected to limit access to the data.

The database will be created and managed at AFRIMS by EDS and stored on a limited access server. To ensure consistency across antimalarial drug efficacy studies, WWARN has listed common variables to be captured for all studies. Such data should include: unique identifier code (UIC), pertinent demographic data, treatment group assignment, parasitologic data such as but not limited to parasitemia, gametocytemia, speciation etc, volunteer clinical data and safety laboratory test results. Additional data collected for study completeness will also be included in this database (i.e., past medical history, vital signs etc).

CONFIDENTIAL

Volunteer names will also be added to the Volunteer Registry Database as required by the US Army Medical Research and Materiel Command (USAMRMC) whenever human volunteers are used in research studies. This database is maintained only for volunteer safety and will be kept in a secure location at USAMRMC in Fort Detrick, MD. The purpose of the database is to allow the investigators and/or MRMC regulatory officials to contact volunteers who have participated in US Army biomedical research studies in the event that new information becomes available that could potentially affect volunteer health and/or safety. It is the policy of USAMRMC that data sheets are to be completed on all volunteers participating in research for entry into the U.S. Army Medical Research and Materiel Command Volunteer Registry Database. The information to be entered into this confidential database includes name, address, adverse events which may occur during study participation, study name, and dates. The intent of the database is twofold: first, to readily answer questions concerning an individual's participation in research sponsored by the USAMRMC; and second, to ensure that the USAMRMC can exercise its obligation to ensure research volunteers are adequately warned (duty to warn) of risks and to provide new information as it becomes available. The information will be stored at the USAMRMC for a minimum of 75 years. The only other documents to include individually identifiable information will be the identification log, a source document that will remain securely stored with other source documents at CNM. No individually identifiable volunteer information will otherwise be transported, transmitted or otherwise removed from Cambodia with the exception of information required for the VRD which will be stored at USAMRMC HQ as outlined above.

9.4 Risk to Volunteers and Precautions to Minimize Risk

The main risks to individual volunteers as a result of study participation beyond those related to the clinical diagnosis and treatment of malaria include:

- Adverse effects from treatment with antimalarials. DHA-piperaquine has been adopted as the first line antimalarial agent by the National Malaria Control Program in Cambodian (CNM). The study is designed in part to evaluate the safety and tolerability of a standard 3-day regimen of DHA-piperaquine Piperaquine can cause prolonged QT interval; however, other large regulated clinical trials submitted to the European Medicines Agengy (see Section 3.3) showed that DP at the doses used in this study did not cause a clinically significant prolongation of the QT interval. In AFRIMS trial WR 1849, it was found that a 2 day compressed course of therapy caused significant QTcF prolongation, and the study was halted with recommendations to use the standard 3 day course, and to avoid giving piperaquine to patients within 3 hours of a meal (see Section 3.3). In this study, EKG monitoring will take place with oversight from a Cardiac Data Safety Monitoring Board (see Appendix F, DSMB Charter). Protocol safety criteria have been modified in accordance with those used in protocol WR 1737 (AFRIMS Malaria Cohort and Treatment Study, conducted 2010-2011) to account for and control the potential confounding

CONFIDENTIAL

effects of malaria on the electrocardiogram due to fever and tachycardia. The other potential antimalarials used in this study will all be prescribed in accordance with the study protocol and current national treatment guidelines. In general, potential medications used to treat malaria are well tolerated. Volunteers will be followed by a trained team of clinical malaria researchers with particular attention to potential side effects, and study treatment will be directly observed. Volunteers in this study will be followed up more closely and for longer duration by a dedicated study team, and will have an enhanced level of care compared to malaria volunteers receiving standard of care in Cambodia (non-DOT and more limited follow-up visits).

- Phlebotomy can cause discomfort and pain at venipuncture sites. Volunteers will be counseled to return to the clinic if local infection is suspected. The total volume of blood drawn in this study for volunteers will be up to approximately ~111 ml over 42 days. The maximum draw on any day will be on Days 0 of treatment when ~36 ml will be drawn. Subjects treated for recurrences of malaria, or treated for severe malaria will have additional blood drawn for study-mandated laboratory procedures and for appropriate clinical management if warranted. Volunteers with G6PD deficiency who are referred for primaquine therapy will have an additional ~12-16 mL drawn during treatment with primaquine therapy to monitor for potential hemolysis.

Risks associated with confidentiality: There is also the risk of a breach in confidentiality; however precautions will be taken to minimize this risk. All study communications, lab samples, and documents will be identified by a study code. These documents will not contain any study subject names or identifiable information. The volunteers who agree to be screened will be first assigned a unique screening code. If the volunteer is found to be eligible, they will be assigned a unique subject identification code upon enrollment. The lab samples sent to reference labs as described will contain only subject code. Study information and records will be maintained in a secure storage facility in Phnom Penh, Cambodia.

Although there is little or no risk in G6PD-normal patients, primaquine has the potential to induce hemolytic anemia in G6PD-deficient patients, particularly when given as a anti-relapse course (14 days). Both qualitative and quantitative testing for G6PD deficiency using FDA-approved test kits will be performed on all subjects to reduce the chances of misdiagnosis. Quantitative testing will be performed, and results will be calculated based on same-day hemoglobin values from the complete blood count. Severe deficiency (WHO Class I or II) will be defined as 10% or less of the lower limit of normal activity (in G6PD activity units per gram of hemoglobin) established for the quantitative assay system. Subjects with severe deficiency will not be enrolled. Subjects with mild to moderate (Class III, IV or V) deficiency will be enrolled and may be randomized to receive the onetime dose of primaquine. While only a single dose of 45mg primaquine will be administered to volunteers for this study, careful monitoring of blood counts will occur. A CBC will be obtained at enrollment with repeat CBC on day 3 following the primaquine dose. Additional CBC

CONFIDENTIAL

monitoring will be performed if the hematocrit drops more than 10% post-dose with repeat CBCs on day 7 and day 14 after enrollment. The experience from the previous study (WRAIR 1737) conducted in Cambodia by AFRIMS in collaboration with RCAF demonstrated that there were no severe adverse events due to G6PD deficiency seen in any volunteer who received 45mg of primaquine for 8 weeks. As described in Section 3.5, only 2 volunteers had drops in the hematocrit of greater than 10% which resolved without incident.

Risks associated with pregnancy: Both malaria and artemisinin antimalarials given during the first trimester can have deleterious effects on the developing fetus; therefore, pregnant women are excluded from the study. Female volunteers will have urinary pregnancy tests performed at screening, at any time of recrudescence and at the last follow-up visit (day 42 or alternate day determined by the investigator if participation is extended for receipt of antimalarials).

9.5 Alternatives to Test Article (or Research Treatment)

Volunteers may elect not to participate in the study, and receive standard medical care for malaria which currently includes 2-3 days of non-DOT therapy with an artemisinin-piperaquine or an artesunate-mefloquine combination.

9.6 Benefits to Volunteers

Volunteers will benefit from the increased vigilance provided by the study team and will also benefit from directly observed malaria treatment and careful follow-up by a trained study team. There are no other direct benefits to volunteers from participating in this study. The study will benefit the community as a whole by providing up-to-date information on drug resistance and treatment regimen efficacy which will be provided to Cambodia National Malaria Control Program (CNM).

9.7 Risks to Study Personnel and Precautions to Minimize Risk

There are no additional anticipated risks to study personnel as a result of study participation. AFRIMS SOPs on occupational health and safety will be adhered to at all times, and all staff certified at the appropriate level. Universal precautions will be observed at all times when handling biological specimens.

9.8 Risks to the Environment

None.

CONFIDENTIAL

9.9 Financial Incentives to Volunteers

Compensation will be provided throughout the study, and volunteers will be compensated for all study visits completed if they leave the study prior to completion. The estimated compensation for completion of the trial will be approximately 20,000 Cambodian Riel (approximately US $5 depending on current exchange rates) per follow up visit including screening and enrollment, and unscheduled visits. Volunteers will also receive this same amount of compensation on a daily basis while hospitalized. This compensation takes into consideration lost earnings (for civilian dependent beneficiaries), meals and incidentals arising from participation, and discomfort from phlebotomy. Compensation provided in the study will be outlined in the Informed Consent Document which will be the definitive document detailing volunteer compensation throughout the study. Any future changes in compensation made to IRB-approved Informed Consent Document will supersede the details provided in this section.

9.10 Medical Care for Injury or Illness

In accordance with DoDI 3216.02, appropriate language has been included within the informed consent addressing Research Related Injury. Medical care in case of research-related injury on either an emergency or routine basis will be provided free of charge according to local standard of care by qualified medical personnel at the appropriate facility. Volunteers will not receive additional compensation for injury beyond medical care. Volunteers will be encouraged to discuss this issue with the principal investigator before they enroll in this study. This medical care provision does not constitute a waiver or release of volunteer's legal rights. Adequate provision for RRI will be included as part of the contractual agreement with the National Center for Parasitology, Entomology and Malaria Control.

CONFIDENTIAL

10 ADMINISTRATIVE PROCEDURES

10.1 Institutional Review Board

The protocol and informed consent documents will be provided for the review and approval to all IRBs having jurisdiction over the study prior to implementation. The protocol will require scientific review and approval by the committee at AFRIMS. The protocol will be sent to the University of North Carolina IRB for non-human subjects research determination. The protocol will undergo ethical review and require approval by the U.S. Army Medical Research and Materiel Command Office of Research Protection Human Research Protection Office (USAMRMC ORP HRPO), WRAIR IRB, the National Ethics Committee for Health Research IRB# 1 (NECHR) (FWA# 00010451, IRB # 00003143). All amendments to IRB approved documents must be submitted for review and approval by all applicable institutional review boards prior to implementation. The WRAIR HSPB will report protocol actions to the USAMRMC ORP HRPO as per their current SOP (i.e. UWZC-636 or equivalent), as appropriate.

10.2 Protocol Amendments

Any change or amendment to the protocol affecting study volunteers, study objectives, study design, study procedures, or significant administrative aspects will require a formal amendment to the protocol. Such amendment will be submitted to the WRAIR IRB and NECHR for review and approval prior to implementation.

Administrative changes to the protocol are corrections and/or clarifications that have no effect on the way the study is to be conducted. Such administrative changes will be submitted to the WRAIR IRB and NECHR for review and approval prior to implementation.

The Informed Consent Form and protocol related documents will be revised to reflex the changes of the amendment as appropriate, and will also be reviewed and approved with the amendment.

Continuing Review:

Continuing review reports will be submitted at intervals designated by the WRAIR IRB (or IRB of record) and a final and/or closeout report in accordance with 32 CFR 219 and Army regulations. The continuing review and final and/or closeout reports should be submitted to the NECHR and to the WRAIR IRB, thru the WRAIR HSPB (*wrairhspb@amedd.army.mil*).

If the continuing review is not approved by the NECHR and WRAIR IRB by the anniversary date, all protocol activities must stop at that site until such time as the approval is obtained. A copy of the approved continuing review report and supporting documentation, along with IRB approvals will be submitted to the WRAIR

CONFIDENTIAL

HSPB for processing before review by the WRAIR IRB as soon as these documents become available. Review and approvals by the NECHR, are subject to their policies but must be performed at least annually.

10.3 Study Medication Accountability

Test article will be purchased commercially and will be maintained securely in a locked cabinet by the study team at all times until administered. The study team will maintain a log documenting test article administration.

10.4 Disposition of Data

All data will be retained according to ICH guidelines by the Investigators for 5 years at AFRIMS office in Cambodia. The case report forms and a copy of the final report will be stored in an access-controlled place in the contracted archives of AFRIMS.

10.5 Access to Source Data/Documents

The investigators, research monitor, and other study personnel assigned from National Center for Parasitology, Entomology and Malaria Control (CNM) and their respective representatives are authorized access to the study data as part of their duties and part of their responsibility to protect human volunteers in research. The investigators, research monitor, members of the WRAIR IRB, representatives of the U.S. Army Medical Research and Materiel Command (USAMRMC), representatives of regulatory agencies, and other government agencies are authorized access to the study data as part of their duties and part of their responsibility to protect human volunteers in research.

10.6 Certification of Translation (where applicable)

Investigators will provide documentation that the foreign language version of the consent form is an accurate translation. Documentation of translation will be provided along with the English and foreign language version of the consent forms. Translations of study documents must be approved by the appropriate approving authority for accuracy and completeness of translation. Use the CMD-QP-003-F1, Translation Verification Form or a similar document (e.g. memorandum).

10.7 Protocol Deviations

A significant deviation occurs when there is non-adherence to the IRB approved protocol that has the potential to effect the rights and welfare of the research participant, to increase the risk to the research participant, to change the willingness of the research participant to continue participation, or to compromise the integrity of the study data in such a way that the study objectives cannot be achieved. Significant deviations must be reported promptly to the WRAIR IRB,

CONFIDENTIAL

within 48 hours of becoming aware of the event, and recorded in the study deviation log.

Significant deviations should be promptly report (within 48 hours of the PI becoming aware of the deviation) by telephone (301-319-9940), fax (301-319-9961) or email (wrairhspb@amedd.army.mil) to the WRAIR IRB, thru the WRAIR HSPB, and then must be followed-up in writing within 10 working days from awareness of the deviation.

All other deviations (minor) will be recorded in the study deviation log and provided as part of the continuing review report.

10.8 Compliance Inspections

For reporting pending compliance inspections: Notice of compliance inspections will be immediately reported to the WRAIR Division of Human Subjects Protection by telephone (301-319-9940), fax (301-319-9961) or email (wrairhspb@amedd.army.mil), the local IRB, and the USAMRMC Office of Research Protections upon knowledge of a pending compliance inspection by any governmental agency concerning clinical investigation or research.

10.9 Publication Policy

Results of this study will be presented in scientific forums orally and in written publications in scientific journals. No identifying information for any of the volunteers in the study will be included in any presentation of data or photographs. Publications will be submitted as per Command review policy.

10.10 Responsibilities of Study Personnel

All named personnel are fully qualified to perform the following assigned roles. The Principal Investigators will ensure that all assigned personnel maintain required trainings, licensures and certifications throughout the study. All duties will be performed in accordance with GCP Guidelines.

The Principal Investigators will be responsible for all aspects of the study to include: Protocol design to include all related documents (such as the consent form, case report form, standard operating procedures, etc); supervision and monitoring of research staff; protocol compliance and QA/QC plan execution; timely and accurate reporting of AEs (including SAEs) to IRBs and management of the respective organizations as outlined in the protocol. PIs will also be responsible for clinical and scientific aspects of the study to include volunteer care, data analysis, interpretation and manuscript preparation; continuing review and final study reports and publication. PIs will liaise with study personnel from the different organizations listed

CONFIDENTIAL

as well as local authorities. All duties will be performed in accordance with GCP Guidelines.

The Associate Clinical Investigators will be responsible for multiple aspects of the study to include: Protocol design to include all related documents (such as the consent form, case report form, standard operating procedures, etc); supervision and monitoring of research staff; protocol compliance and QA/QC plan execution; timely and accurate reporting of AEs (including SAEs) to IRBs and management of the respective organizations as outlined in the protocol. AIs will also be responsible for clinical and scientific aspects of the study to include volunteer care, data analysis, interpretation and manuscript preparation; continuing review and final study reports and publication. AIs will liaise with study personnel from the different organizations listed as well as local authorities.

The Associate Laboratory Investigators will be responsible for multiple aspects of laboratory analysis during or arising from the study to include depending on their respective disciplines: method development; assay design; development of standard operating procedures; storage and shipment of samples (where required); data analysis and interpretation; manuscript preparation; supervision and monitoring technical staff in the conduct of procedures based on levels of established training and expertise.

Clinical and Laboratory Research Coordinators will be responsible for coordinating procedures in the field and laboratory to include informed consent, screening and enrollment; study procedures including SOP/SSP instruction and adherence; coordinating the conduct of laboratory procedures; in addition to other duties as assigned by the investigators for which they are qualified.

If military volunteers are screened for the study, an ombudsman independent of the study team will serve as independent advocates for subject welfare, and be present during informed consent sessions. Ombudsmen will also serve as witnesses during the informed consent process. They will also be available to subjects by telephone and/or on request to communicate questions or concerns to the investigative team. One or more ombudsmen will be selected from the civilian community or from the RCAF as long as they are outside the chain of command of subjects being recruited. In cases of military ombudsmen, they will have sufficient rank and authority to permit an independent unbiased determination of subject welfare. For issues that cannot be resolved by the investigators, the ombudsmen will report the matter to the research monitor.

The research monitor will be responsible to ensure that the monitoring of study volunteers from a medical perspective has been done appropriately, to review and report all serious and unexpected adverse events, and to verify that medical care is provided for any such events should they occur and the events is reported to the IRBs.

CONFIDENTIAL

The Clinical Study Monitor will be responsible for regular monitoring of data collection and procedures to ensure that the human volunteer protections, study procedures, laboratory, and data collection processes are of high quality and meet GCP/ICH and regulatory guidelines; and correspond with IRBs as required.

Consultants may assist the study team with protocol design, data analysis, interpretation and manuscript review and preparation. Consultants will not have contact with volunteers or their individually identifiable information. Consultant laboratory investigators from outside institutions may analyze de-identified study specimens which are labeled with subject ID; no subject identifiable information will be provided. In each case, performing laboratory consultants will obtain permission from their respective IRBs and provide this to the IRBs of record.

10.11 Responsibilities of the Research Monitor

In accordance with the DoD Directive (DoDD) 3216.02, all studies determined to be greater than minimal risk [as defined by 32 CFR 219.102(i)] require an independent DoD research monitor. The name of the research monitor is included in the protocol and the curriculum vita has been provided. Note that the DOD definition of a research monitor differs from the industry definition.

The research monitor for this study is a qualified physician, other than the Principal Investigator, not associated with the protocol, who is able to provide medical care to research volunteers for conditions that may arise during the conduct of the study, and who will monitor the volunteers during the conduct of the study. Research monitors shall promptly report discrepancies or problems to the IRB. They shall have the authority to stop a research study in progress, remove individual subjects from a study, and take whatever steps are necessary to protect the safety and well-being of research subjects until the IRB can assess the research monitor's report. The WRAIR IRB is responsible for ensuring that the individual is appropriately qualified to serve in this role.

The research monitor is required to review all unanticipated problems involving risk to subjects or others, serious adverse events and all subject deaths associated with the protocol and provide an unbiased written report of the event. At a minimum, the research monitor must comment on the outcomes of the event or problem and in case of a serious adverse event or death, comment on the relationship to participation in the study. The research monitor must also indicate whether he/she concurs with the details of the report provided by the principal investigator. Reports for events determined by either the investigator or research monitor to be possibly or definitely related to participation and reports of events resulting in death must be promptly forwarded to the USAMRMC ORP HRPO.

CONFIDENTIAL

References (listed in order of citation)

1. Wongsrichanala C, Meshnick SR, Declining Artesunate-Mefloquine Efficacy against Falciparum Malaria on the Cambodia–Thailand Border. *Emerg Infect Dis.* 2008; 14(5): 716–719.

2. Noedl H, Se Y, Schaecher K, et al. Artemisinin Resistance in Cambodia 1 (ARC1) Study Consortium, Evidence of artemisinin-resistant malaria in western Cambodia. *N Engl J Med.* 2008;359(24): 2619-20.

3. Wongsrichanalai, C, Sirichaisinthop, J, Karwacki, JJ, Congpuong, K, Miller, et al, Drug resistant malaria on the Thai-Myanmar and Thai-Cambodian borders. *S Asian J Trop Med Pub Heal.* 2001;(32): 41-49.

4. Wongsrichanalai C, Pickard AL, Wernsdorfer WH, and Meshnick SR, Epidemiology of drug-resistant malaria. *Lancet Infect Dis.* 2002;(2): 209-218.

5. Hyde JE, Mechanisms of resistance of Plasmodium falciparum to antimalarial drugs. *Microbes Infect.* 2002;(4): 165-174.

6. Price RN, and Nosten F, Drug resistant falciparum malaria: clinical consequences and strategies for prevention*. Drug Resist Update.* 2001;(4): 187-196.

7. Bloland PB, Lackritz EM, Kazembe PN, Were JB, Steketee R, and Campbell CC Beyond chloroquine: implications of drug resistance for evaluating malaria therapy efficacy and treatment policy in Africa. *J Infect Dis.* 1993;(167): 932-937.

8. Shretta R, Omumbo, J, Rapuoda B, and Snow RW, Using evidence to change antimalarial drug policy in Kenya. *Trop Med Int Health.* 2000;(5): 755-764.

9. Omar SA, Adagu, IS, Gump, DW, Ndaru NP, and Warhurst DC, *Plasmodium falciparum* in Kenya: high prevalence of drug-resistance-associated polymorphisms in hospital admissions with severe malaria in an epidemic area. *Ann Trop Med Parasitol.* 2001;(95): 661-669.

10. Mberu EK, Mosobo MK, Nzila AM, Kokwaro GO, Sibley CH, and Watkins WM, The changing in vitro susceptibility pattern to pyrimethamine/sulfadoxine in Plasmodium falciparum field isolates from Kilifi, Kenya. *Am J Trop Med Hyg.* 2000;(62): 396-401.

11. Khan B, Omar S, Kanyara JN, Warren-Perry M, Nyalwidhe J, Peterson DS, et al, Antifolate drug resistance and point mutations in *Plasmodium falciparum* in Kenya. *Trans R Soc Trop Med Hyg.* 1997;(91): 456-460.

CONFIDENTIAL

12. Vijaykadga S, Rojanawatsirivej C, Cholpol S, Phoungmanee D, Nakavej A,et al, In vivo sensitivity monitoring of mefloquine monotherapy and artesunate-mefloquine combinations for the treatment of uncomplicated falciparum malaria in Thailand in 2003. *Trop Med Int Health*. 2006;11(2):211-9.

13. Bethell D, Se Y, Lon C, Tyner S, Saunders S, Sriwichai S, Darapiseth S, et al, Artesunate Dose Escalation for the Treatment of Uncomplicated Malaria in a Region of Reported Artemisinin Resistance: A Randomized Clinic Trial. *PLoS One.* 2011; 6(5): e19283.

14. Hung TY, Davis TM, Ilett KF, Karunajeewa H, Hewitt S, et al, Population pharmacokinetics of piperaquine in adults and children with uncomplicated falciparum or vivax malaria. *Br J Clin Pharmacol.* 2004;57:253–262.

15. Tarning J, Ashley EA, Lindegardh N, Stepniewska K, Phaiphun L, et al, Population pharmacokinetics of piperaquine after two different treatment regimens of dihydroartemisinin-piperaquine in patients with acute uncomplicated *Plasmodium falciparum* malaria in Thailand. *Antimicrob Agents Chemother.* 2008;52:1052–61.

16. Zwang J et al. Safety and Efficacy of Dihydroartemisinin-Piperaquine in Falciparum Malaria: A Prospective Multi-Centre Individual Patient Data Analysis. *PLoS One.* 2009; 4(7): e6358.

17. Janssens B , van Herp M et al., A randomized open study to assess the efficacy and tolerability of dihydroartemisinin–piperaquine for the treatment of uncomplicated falciparum malaria in Cambodia. *Trop Med & Intnl Health.* 2007; 12 (2): 251 – 259.

18. MMV Press Release 2009 - http://www.asia-lifesciences.com/press-174-sigmatau-industriefarmaceuticheriunite-emea-novelantimalarial-press6.html

19. Mytton OT, Ashley EA, et al. Short Report: Electrocardiographic Safety Evaluation of Dihydroartemisinin–Piperaquine in the Treatment of Uncomplicated falciparum Malaria. *Am J Trop Med Hyg*. 2007; 77(3): 447–450

20. World Health Organization (WHO), Guidelines for the Treatment of Malaria 2nd ed., 2010.

21. Bousema T and Drakeley C, Epidemiology and Infectivity of *Plasmodium falciparum* and *Plasmodium vivax* Gametocytes in relation to Malaria Control and Elimination. *Clin Micro Rev*. 2011; 24(2): 377-410.

CONFIDENTIAL

22. Coleman RE, Kumpitak C, Ponlawat A, Maneechai N, Phunkitchar V, et al, Infectivity of Asymtpomatic *Plasmodium*-Infected Human Populations to *Anopheles dirus* Mosquitoes in Western Thailand, *J Med Ento*. 2004. 41(2):201-208.

23. Schneider P, Bousema JT, Gouagna LC, Otieno S, Van de Vegte-Bolmer M, et al, Submicroscopic *Plasmodium falciparum* gametocyte densities frequently result in mosquito infection. *Am J Trop Med Hyg*. 2007; 76(3): 470-474.

24. Bousema T, Schneider P, Gouagna LC, Drakeley CJ, Tostmann A, Houben R, et al, Moderate Effect of Artemisinin-Based Combination Therapy on Transmission of *Plasmodium falciparum*. *J Infec Dis*. 2006;193: 1151-1159.

25. Shekalaghe SA, Bousema JT, Kunei KK, Lushino P, Masokoto A, et al, Submicroscopic *Plasmodium falciparum* gametocyte carriage is common in an area of low and seasonal transmission in Tanzania. *Trop Med Intl Heal*. 2007; 12(4): 547-553.

26. Okell LC, Ghani AC, Lyons E, Drakeley C, Submicroscopic Infection in *Plasmodium falciparum*-Endemic Populations: A Systematic Review and Meta-Analysis. *J Infec Dis*. 2009;200: 1509-1517.

27. Targett G, Drakeley C, Jawara M, von Seidlein L, Coleman R, Deen J et al, Artesunate Reduces but Dose Not Prevent Posttreatment Transmission of *Plasmodium falciparum* to *Anopheles gambiae*. *J Infec Dis*. 2001; 183:1254-1259.

28. Robert V, Awono-Ambene HP, Le Hesran JY, Trape JF, Gametocytemia and infectivity to mosquitoes of patients with uncomplicated Plasmodium falciparum malaria attacks treated with chloroquine or sulfadoxine plus pyrimethamine. *Am J Trop Med Hyg*. 2000; 62(2): 210-216

29. Dunyo S, Milligan P, Edwards T, Sutherland C, Targett G, Pinder M, Gametocytemia after Drug Treatment of Asymptomatic *Plasmodium falciparum*. *PLoS Clin Trials*. 2006; 1(4): e20.

30. Pukrittayakamee S, Chotivanich K, Chantra A, Clemens R, Looareesuwan S, et al, Activities of Artesunate and primaquine against Asexual- and Sexual-Stage Parasites in Falciparum Malaria. Anti Agen Chemo. 2004; 48(4): 1329-1334.

31. Chotivanich K, Sattabongkot J, Udomsangpetch R, Looareesuwan S, et al, Transmission-Blocking Activities of Quinine, Primaquine, and Artesunate. *Antimicro Agen Chemo*. 2006; 50(6): 1927-1930.

CONFIDENTIAL

32. Shekalaghe S, Drakeley C, Gosling R, Ndaro A, van Meegeren M, et al, Primaquine Clears Submicroscopic *Plasmodium falciparum* Gametocytes that Persist after Treatment with Sulphadoxine-Pyrimethamine and Artesunate. *PLoS One* 2006; 2(10). e1023.

33. Smithius F, Kyaw MK, Phe O, Win T, Aung PP, Oo APP, Naing AL, Nya MY et al, Effectiveness of five artemisinin combination regimens with or without primaquine in uncomplicated falciparum malaria: an open-label randomised trial. *Lancet*. 2010; 10: 673-681.

34. El-Sayed B, El-Zaki, SE, Babiker H, Gadalla N, Ageep T, Mansour F, et al, A Randomized Open-Label Trial of Artesunate-Sulfadoxine-Pyrimethamine with or without Primaquine for Elimination of Sub-Microscopic *P. falciparum* Parasitemia and Gametocyte Carriage in Eastern Sudan. *PLoS One*. 2007; 2(12): e1311.

35. Barnes KI, Little F, Mabuza A, Mngomezulu N, Govere J, Durrheim D et al, Increased Gametocytemia after Treatment: An Early Parasitological Indicator of Emerging Sulfadoxine-Pyrimethamine Resistance in Falciparum Malaria. *J Infec Dis*. 2008; 197: 1607-1613.

36. Louicharoen C, Nuchprayoon I, G6PD Viangchan (871G>A) is the most common G6PD-deficient variant in the Cambodian population. J Hum Genetics, 2005. 50 (9): 448-52.

37. Everett WD, Yoshida A, Pearlman E. Hemoglobin E and glucose-6-phosphatase deficiency in the Khmer Air Force (Cambodia). *Am J Trop Med Hyg*, 1977. 26 (4): 597-601.

38. Alving AS, Johnson CF, et al, Mitigation of the haemolytic effect of primaquine and enhancement of its action against exoerythrocytic forms of the Chesson strain of *Plasmodium vivax* by intermittent regimens of drug administration: a preliminary report. *Bull World Health Organ*. 1960; 22: 621-31.

39. Kyaw MP, Myint O, et al, The use of primaquine in malaria infected patients with red cell glucose 6-phosphate dehydrogenase (G6PD) deficiency in Myanmar. *S Asian J Trop Med Pub Heal* 1994; 25(4): 710-3.

40. Charoenlarp P, Areekul S, et al, The haemolytic effect of a single dose of 45 mg of primaquine in G-6-PD deficient Thais. *J Med Assoc Thai*. 1972; 55(11): 631-8.

41. Ziai M, Amirhakimi GH, et al, Malaria prophylaxis and treatment in G-6-PD deficiency. An observation on the toxicity of primaquine and chloroquine. *Clin Pediatr (Phila)*. 1967; 6(4): 242-3.

CONFIDENTIAL

42. Reeve PA, Toaliu H, et al, Acute intravascular haemolysis in Vanuatu following a single dose of primaquine in individuals with glucose-6-phosphate dehydrogenase deficiency. *J Trop Med Hyg*. 1992;95(5): 349-51.

43. Krudsood S et al, Dose ranging studies of new artemisinin-piperaquine fixed combinations compared to standard regimens of artemisinin combination therapies for acute, uncomplicated falciparum malaria. 2007; 38 (6): 971-978

44. CIA The World Factbook 2010, https://www.cia.gov/library/publications/the-world-factbook/geos/cb.html

45. Liu C et al. Pharmacokintetics of Piperaquine after single and multiple oral Administrations in Healthy Volunteers. *Yakugaku Zasshi*. 2007;127(10): 1709-1714

46. Bangchang KN, Karbwang J, & Back DJ, Primaquine metabolism by human liver microsomes: effect of other antimalarial drugs. *Biochem Pharmacol*. 1992; 44:587-590.

47. Barradell LB and Fitton A, Artesunate: a review of its pharmacology and therapeutic efficacy in the treatment of malaria. *Drugs*. 1995a; 50:714-771

48. Trager W, Jensen JB, Continuous culture of *Plasmodium falciparum*: its impact on malaria research. *Int J Parasitol. 1997;* **27** (9): 989–1006.

49. Anderson TJC, Su XZ, Bockarie M, Lagoh M, Day KP, Twelve microsatellite markers for characterization of *Plasmodium falciparum* from finger-prick blood samples. *Parasit.* 1999;119: 113-125

50. Su XZ, Ferdig MT, Huang Y, Huynh CQ, Liu A, You J, Wootton JC, Wellems TE, A Genetic Map and Recombination Parameters of the Human Malaria Parasite *Plasmodium falciparum. Science*. 1999;286(5443): 1351-1353

51. Vinayak S, Alam MT, Mixson-Hayden T, McCollum AM, Sem R, et al, Origin and Evolution of Sulfadoxine Resistant *Plasmodium falciparum*. *PLoS Pathog*. 2010; 6(3): e1000830.

52. Noedl H, Se Y, Sriwichai S, Schaecher K, Teja-isavadharm P, Smith B, et al, Artemisinin Resistance in Cambodia: A Clinical Trial Designed to Address an Emerging Problem in Southeast Asia. *Clin Inf Dis*. 2010; 51(11): e82-89.

53. Noedl H, Wernsdorfer WH, Miller RS and Wongsrichanalai C, Histidine rich protein II, a novel approach to anti-malarial drug susceptibility testing. *Antimicrob Agent Chemo*. 2002; 46(6): 1658-64.

CONFIDENTIAL

54. Noedl, H., Teja-Isavadharm, P. and Miller R.S. Nonisotopic, semi-automated Plasmodium falciparum bioassay for measurement of antimalarial drug levels in serum or plasma. *Antimicrob Agents Chemother*, 2004. 48: p. 4485-4487.

55. Bland JM, Altman DG, Comparing methods of measurement: why plotting difference against standard method is misleading. *Lancet*. 1995;346(8982):1085-7.

Appendices

Appendix A: Cambodia National Malaria Program Treatment Guidelines (attachment)

Appendix B: Dihydroartemisinin-piperaquine package insert (attachment)

Appendix C: Primaquine package insert (attachment)

Appendix D: Statistical Analysis Plan (World-wide Antimalarial Research Network attachment)

Appendix E. Recent publicly available information from Sigma-tau pharmaceuticals – package insert and labeling information describing cardiac safety issues with DHA-piperaquine (Eurartesim) (attachment)

Appendix F. DSMB Charter

CONFIDENTIAL
